# The global, regional, and national burden of pancreatic cancer and its attributable risk factors in 195 countries and territories, 1990–2017: a systematic analysis for the Global Burden of Disease Study 2017

**DOI:** 10.1016/S2468-1253(19)30347-4

**Published:** 2019-10-21

**Authors:** GBD 2017 Pancreatic Cancer Collaborators, Akram Pourshams, Sadaf G Sepanlou, Kevin S Ikuta, Catherine Bisignano, Saeid Safiri, Gholamreza Roshandel, Mehdi Sharif, Morteza Khatibian, Christina Fitzmaurice, Molly R Nixon, Nooshin Abbasi, Mohsen Afarideh, Elham Ahmadian, Tomi Akinyemiju, Fares Alahdab, Tahiya Alam, Vahid Alipour, Christine A Allen, Nahla Hamed Anber, Alireza Ansari-Moghaddam, Jalal Arabloo, Alaa Badawi, Mojtaba Bagherzadeh, Yaschilal Muche Belayneh, Belete Biadgo, Ali Bijani, Antonio Biondi, Tone Bjørge, Antonio M Borzì, Cristina Bosetti, Andrey Nikolaevich Briko, Nikolay Ivanovich Briko, Giulia Carreras, Félix Carvalho, Jee-Young J Choi, Dinh-Toi Chu, Anh Kim Dang, Ahmad Daryani, Dragos Virgil Davitoiu, Gebre Teklemariam Demoz, Rupak Desai, Subhojit Dey, Hoa Thi Do, Huyen Phuc Do, Aziz Eftekhari, Alireza Esteghamati, Farshad Farzadfar, Eduarda Fernandes, Irina Filip, Florian Fischer, Masoud Foroutan, Mohamed M Gad, Silvano Gallus, Birhanu Geta, Giuseppe Gorini, Nima Hafezi-Nejad, James D Harvey, Milad Hasankhani, Amir Hasanzadeh, Soheil Hassanipour, Simon I Hay, Hagos D Hidru, Chi Linh Hoang, Sorin Hostiuc, Mowafa Househ, Olayinka Stephen Ilesanmi, Milena D Ilic, Seyed Sina Naghibi Irvani, Nader Jafari Balalami, Spencer L James, Farahnaz Joukar, Amir Kasaeian, Tesfaye Dessale Kassa, Andre Pascal Kengne, Rovshan Khalilov, Ejaz Ahmad Khan, Amir Khater, Fatemeh Khosravi Shadmani, Jonathan M Kocarnik, Hamidreza Komaki, Ai Koyanagi, Vivek Kumar, Carlo La Vecchia, Platon D Lopukhov, Farzad Manafi, Navid Manafi, Ana-Laura Manda, Fariborz Mansour-Ghanaei, Dhruv Mehta, Varshil Mehta, Toni Meier, Hagazi Gebre Meles, Getnet Mengistu, Tomasz Miazgowski, Mehdi Mohamadnejad, Abdollah Mohammadian-Hafshejani, Milad Mohammadoo-Khorasani, Shafiu Mohammed, Farnam Mohebi, Ali H Mokdad, Lorenzo Monasta, Maryam Moossavi, Rahmatollah Moradzadeh, Gurudatta Naik, Ionut Negoi, Cuong Tat Nguyen, Long Hoang Nguyen, Trang Huyen Nguyen, Andrew T Olagunju, Tinuke O Olagunju, Alyssa Pennini, Mohammad Rabiee, Navid Rabiee, Amir Radfar, Mahdi Rahimi, Goura Kishor Rath, David Laith Rawaf, Salman Rawaf, Robert C Reiner, Nima Rezaei, Aziz Rezapour, Anas M Saad, Seyedmohammad Saadatagah, Amirhossein Sahebkar, Hamideh Salimzadeh, Abdallah M Samy, Juan Sanabria, Arash Sarveazad, Monika Sawhney, Mario Sekerija, Pavel Shabalkin, Masood Ali Shaikh, Rajesh Sharma, Sara Sheikhbahaei, Reza Shirkoohi, Sudeep K Siddappa Malleshappa, Mekonnen Sisay, Kjetil Soreide, Sergey Soshnikov, Rasoul Sotoudehmanesh, Vladimir I Starodubov, Michelle L Subart, Rafael Tabarés-Seisdedos, Degena Bahray Bahrey Tadesse, Eugenio Traini, Bach Xuan Tran, Khanh Bao Tran, Irfan Ullah, Marco Vacante, Amir Vahedian-Azimi, Elena Varavikova, Ronny Westerman, Dawit Dawit Zewdu Wondafrash, Rixing Xu, Naohiro Yonemoto, Vesna Zadnik, Zhi-Jiang Zhang, Reza Malekzadeh, Mohsen Naghavi

## Abstract

**Background:**

Worldwide, both the incidence and death rates of pancreatic cancer are increasing. Evaluation of pancreatic cancer burden and its global, regional, and national patterns is crucial to policy making and better resource allocation for controlling pancreatic cancer risk factors, developing early detection methods, and providing faster and more effective treatments.

**Methods:**

Vital registration, vital registration sample, and cancer registry data were used to generate mortality, incidence, and disability-adjusted life-years (DALYs) estimates. We used the comparative risk assessment framework to estimate the proportion of deaths attributable to risk factors for pancreatic cancer: smoking, high fasting plasma glucose, and high body-mass index. All of the estimates were reported as counts and age-standardised rates per 100 000 person-years. 95% uncertainty intervals (UIs) were reported for all estimates.

**Findings:**

In 2017, there were 448 000 (95% UI 439 000–456 000) incident cases of pancreatic cancer globally, of which 232 000 (210 000–221 000; 51·9%) were in males. The age-standardised incidence rate was 5·0 (4·9–5·1) per 100 000 person-years in 1990 and increased to 5·7 (5·6–5·8) per 100 000 person-years in 2017. There was a 2·3 times increase in number of deaths for both sexes from 196 000 (193 000–200 000) in 1990 to 441 000 (433 000–449 000) in 2017. There was a 2·1 times increase in DALYs due to pancreatic cancer, increasing from 4·4 million (4·3–4·5) in 1990 to 9·1 million (8·9–9·3) in 2017. The age-standardised death rate of pancreatic cancer was highest in the high-income super-region across all years from 1990 to 2017. In 2017, the highest age-standardised death rates were observed in Greenland (17·4 [15·8–19·0] per 100 000 person-years) and Uruguay (12·1 [10·9–13·5] per 100 000 person-years). These countries also had the highest age-standardised death rates in 1990. Bangladesh (1·9 [1·5–2·3] per 100 000 person-years) had the lowest rate in 2017, and São Tomé and Príncipe (1·3 [1·1–1·5] per 100 000 person-years) had the lowest rate in 1990. The numbers of incident cases and deaths peaked at the ages of 65–69 years for males and at 75–79 years for females. Age-standardised pancreatic cancer deaths worldwide were primarily attributable to smoking (21·1% [18·8–23·7]), high fasting plasma glucose (8·9% [2·1–19·4]), and high body-mass index (6·2% [2·5–11·4]) in 2017.

**Interpretation:**

Globally, the number of deaths, incident cases, and DALYs caused by pancreatic cancer has more than doubled from 1990 to 2017. The increase in incidence of pancreatic cancer is likely to continue as the population ages. Prevention strategies should focus on modifiable risk factors. Development of screening programmes for early detection and more effective treatment strategies for pancreatic cancer are needed.

**Funding:**

Bill & Melinda Gates Foundation.

## Introduction

Cancer incidence and mortality are rapidly increasing worldwide.^1^, ^2^ This increase is thought to be due to population growth and ageing, as well as changes in the prevalence of the main risk factors for cancer, several of which are associated with socioeconomic development.^1^, ^2^

Pancreatic cancer remains one of the cancers with the poorest prognosis, with an overall 5-year survival rate of about 5%, without much difference between high-income countries and low-income and middle-income countries.^3^ On the basis of the results of the previous iteration of the Global Burden of Diseases, Injuries, and Risk Factors Study (GBD), pancreatic cancer ranked eighth among cancers in mortality and 14th in incidence in 2016.^1^ Pancreatic cancer incidence and mortality vary considerably in the world.^1^ The highest incidence and mortality rates of pancreatic cancer are found in high-income countries.^2^ Although the causes of pancreatic cancer are still insufficiently understood, certain risk factors have been identified, such as smoking, obesity, and diabetes.^4^ These risk factors probably explain some of the national variation.

**Research in context**

**Evidence before this study**

Pancreatic cancer was estimated as the seventh leading cause of cancer death in both sexes worldwide in 2018, on the basis of the Global Cancer Incidence, Mortality and Prevalence 2018 estimates, from 185 countries, using subregional rather than national data. Because of the poor prognosis of pancreatic cancer, there were almost as many deaths (n=432 000) as there were cases (n=459 000). The rates reported were three times to four times higher in higher Human Development Index countries, with incidence rates being highest in Europe, North America, Australia, and New Zealand, and lowest in south central Asia. To our knowledge, there were no estimates of temporal patterns, trends, age patterns, years of life lost, disability-adjusted life-years, and associated risk factors of pancreatic cancer at national, regional, global, and socioeconomic levels before the Global Burden of Disease Study (GBD).

**Added value of this study**

We present estimates of the global burden of pancreatic cancer based on results from GBD 2017, which are reported by sex and age groups for 195 countries and territories from 1990 to 2017. We also investigated the association of socioeconomic development status with incidence and mortality caused by pancreatic cancer at the national level. We believe that this analysis provides the most comprehensive picture of the burden of pancreatic cancer to date. Examining trends of pancreatic cancer from 1990 to 2017 and comparisons across populations offers important information about the changing burden of pancreatic cancer to aid in the allocation of necessary resources at local levels to help control this lethal cancer.

**Implications of all the available evidence**

The incidence and mortality rates of pancreatic cancer increased in almost all countries and territories from 1990 to 2017. With population growth and increases in longevity, clinicians and policy makers might expect a further substantial rise in the absolute number of pancreatic cancer cases, particularly in low-income and middle-income nations. Existing data gaps are a major challenge for policy making at the regional and national scale. To our knowledge, this study is the first effort to provide comprehensive worldwide estimates of the burden, epidemiological features, and risk factors of pancreatic cancer. Future studies should explore the predictors of these epidemiological trends to help policy makers implement cost-effective interventions for prevention, early detection, and control of pancreatic cancer.

Data about incidence and trends of pancreatic cancer and its risk factors are scarce, specifically in nuanced time and location dimensions. GBD is the first comprehensive and systematic effort to report the incidence of and mortality and disability caused by pancreatic cancer and its risk factors, using an extensive set of data sources and novel statistical methods in seven super-regions, 21 regions, and 195 countries and territories, for both sexes and 20 age groups, from 1990 to 2017. To our knowledge, this study is the first to investigate the association between development status (measured by the Socio-demographic Index [SDI]) and pancreatic cancer incidence and mortality at the national level.

## Methods

### Overview

This study is part of GBD. In the latest iteration, GBD 2017, 359 diseases and injuries, 282 causes of death, and 84 risk factors were estimated. The rationale, methods, and summary results of GBD 2017 have been published previously.^5^, ^6^, ^7^, ^8^ Rates and numbers of deaths, incident cases, years of life lost (YLLs) as a result of premature death, years lived with disability (YLDs), and disability-adjusted life-years (DALYs) were reported for both males and females, 17 age groups, and 195 countries and territories.

The rates were age-standardised according to the world population estimated by the GBD study.^9^ 95% uncertainty intervals (UIs) were reported for all estimates, including all sources of uncertainty arising from measurement error, systematic biases, and modelling. This study is compliant with the Guidelines for Accurate and Transparent Health Estimates Reporting (GATHER).

### Data sources

We considered all cancers coded as C25–C25.9 in the 10th revision of the International Classification of Diseases to be pancreatic cancer and mapped them to the GBD cause list.^5^, ^7^ For this study, we used GBD 2017 vital registration and sample vital registration (19 321 site-years of data) and cancer registry (4472 site-years) data.^7^ Vital registration systems include vital event data from all residents in a population, including causes of death. Sample vital registration systems include nationally representative data from which birth rates, death rates, and causes of death can be estimated. Cancer registries include data on all cancer patients in a defined population, typically from a particular location. Detailed information on data sources used in this study can be found on the GBD 2017 Data Input Sources Tool website.

### Mortality estimates

Data coverage and quality were higher for mortality data than for other measures of pancreatic cancer burden. The cancer registry mortality estimates that were uploaded into the causes of death database were derived from cancer registry incidence data that had been transformed to mortality estimates through the use of mortality-to-incidence ratios (MIRs). We modelled MIRs using the locations that had both incidence and mortality data for the same year. The initial MIR model used a linear-step mixed-effects model with logit link functions. The resulting estimates were then smoothed over place and time, and adjusted using spatiotemporal Gaussian process regression (see appendix of reference 10).^10^ The vital registration mortality, as well as the cancer registry mortality estimates computed from MIRs, were used as inputs for a Cause of Death Ensemble model.^7^, ^11^

### Non-fatal estimates

Pancreatic cancer incidence was computed by dividing the final mortality estimates by the MIR. Four sequelae were defined for pancreatic cancer—diagnosis and primary therapy phase, controlled phase, metastatic phase, and terminal phase.^1^ The diagnosis and primary therapy phase was defined as 4·1 months, the disseminated and metastatic phase as 2·54 months, and terminal phase as 1·0 month.^12^, ^13^ The remaining time was assigned to the controlled phase. Following this process, to estimate the sequelae-specific YLDs, we multiplied each sequela-specific prevalence rate by a sequela-specific disability weight. Each of the four sequelae had defined disability weights that ranged from 0·049 to 0·540 (appendix p 8). DALYs were calculated as the sum of YLDs and YLLs.

### SDI

We used the SDI to determine the relationship between pancreatic cancer incidence and mortality rates with development status at national and regional levels. The SDI ranges from 0 (worst) to 1 (best) and is composed of the total fertility rate among women under the age of 25 years, mean education for individuals aged 15 years and older, and lag-distributed income per capita.^5^, ^6^, ^7^ Components were extracted using principal components analysis. Each component was given equal weight, and the final SDI score was computed as the geometric mean of each of the components.

### Risk factors

We used the comparative risk assessment framework to estimate the proportion of deaths and DALYs attributable to three recognised risk factors for pancreatic cancer: smoking, high fasting plasma glucose, and high body-mass index (BMI). We used the counterfactual scenario of theoretical minimum risk exposure level to model the population attributable fraction. The definitions of the framework have already been published.^8^

### Role of the funding source

The funder of the study had no role in study design, data collection, data analysis, data interpretation, or writing of the report. The corresponding author had full access to all the data in the study and the final responsibility for the decision to submit for publication.

## Results

The number of incident cases of pancreatic cancer in both sexes increased 2·3 times from 195 000 (95% UI 192 000–199 000) incident cases in 1990 to 448 000 (439 000–456 000) cases in 2017 globally (appendix p 19). In 2017, 51·9% (232 000 [210 000–221 000]) of the total incident cases occurred in males, compared with 52·1% (102 000 [99 000–106 000]) in 1990.

The global age-standardised incidence rate was 5·0 (95% UI 4·9–5·1) per 100 000 person-years in 1990, which increased to 5·7 (5·6–5·8) per 100 000 person-years in 2017 (appendix p 19). Globally, there were 9·1 million (8·9–9·3) DALYs due to pancreatic cancer in 2017. This was a 2·1 times increase from 4·4 million (4·3–4·5) DALYs in 1990 (appendix p 27). 99% of all DALYs in all years were due to YLLs (appendix p 7).

In 2017, pancreatic cancer caused 441 000 (95% UI 433 000–449 000) deaths globally, including 226 000 (51·3%; 219 000–233 000) deaths among males and 215 000 (48·7%; 211 000–220 000) deaths among females. There was a 2·3 times (125% [118–131]) increase in the number of deaths globally from 1990 to 2017, increasing from 196 000 (193 000–200 000) deaths for both sexes combined in 1990. The age-standardised death rate increased by 10·4% (7·0–13·0), from 5·1 (5·0–5·2) per 100 000 person-years in 1990 to 5·6 (5·5–5·7) per 100 000 person-years in 2017 (appendix p 11). The age-standardised death rate in males was 5·7 (5·6–5·9) per 100 000 person-years in 1990 and 6·3 (6·1–6·5) per 100 000 person-years in 2017. The equivalent findings for females were 4·5 (4·5–4·6) per 100 000 person-years in 1990 and 5·0 (4·9–5·1) per 100 000 person-years in 2017.

The age-standardised death rate was highest in the high-income super-region across all years from 1990 to 2017: 8·1 (8·1–8·2) per 100 000 person-years in 1990 and 8·6 (8·5–8·8) per 100 000 person-years in 2017 (appendix p 2). Central Europe, eastern Europe, and central Asia ranked second at 6·8 (6·5–7·0) per 100 000 person-years in 1990 and 7·6 (7·5–7·8) per 100 000 person-years in 2017. South Asia had the lowest rates: 1·6 (1·4–1·8) per 100 000 person-years in 1990 and 2·9 (2·7–3·0) per 100 000 person-years in 2017. The pattern of age-standardised incidence rates in super-regions was similar to the pattern we observed for age-standardised death rate (appendix p 2). The pattern of age-standardised incidence and death rates was also similar between sexes (data not shown).

**Figure 1:**
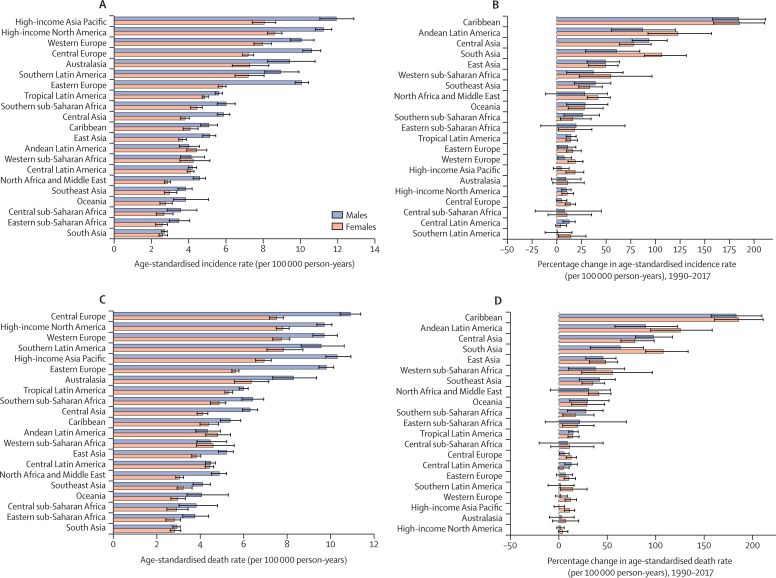
Levels and trends in age-standardised incidence and death rates of pancreatic cancer across 21 GBD regions by sex (A) The age-standardised incidence rates of pancreatic cancer in 2017. (B) The percentage change in age-standardised incidence rate of pancreatic cancer from 1990 to 2017. (C) The age-standardised death rates of pancreatic cancer in 2017. (D) The percentage change in age-standardised death rate of pancreatic cancer from 1990 to 2017. GBD=Global Burden of Diseases, Injuries, and Risk Factors Study.

Age-standardised incidence and death rates increased in all GBD regions from 1990 to 2017 (figure 1). High-income North America and western Europe were among the top three regions for highest age-standardised rates of both incidence and deaths in 2017, with high-income Asia Pacific and central Europe also in the top three for highest age-standardised rate of incidence and death, respectively (figure 1). These regions all had smaller increases in age-standardised rates of incidence and deaths from 1990 to 2017 than many other regions (figure 1B, D; appendix pp 11–26). The lowest age-standardised incidence and death rates in 2017 were observed in south Asia and eastern and central sub-Saharan Africa (figure 1A, C). The Caribbean, Andean Latin America, and central Asia had the highest percentage change in both incidence and death rates from 1990 to 2017 (figure 1B, D). The age-standardised rates for both incidence and death were higher among males than females in almost all regions and all years from 1990 to 2017, with the exception of Andean Latin America and western sub-Saharan Africa (figure 1A, C).

**Figure 2:**
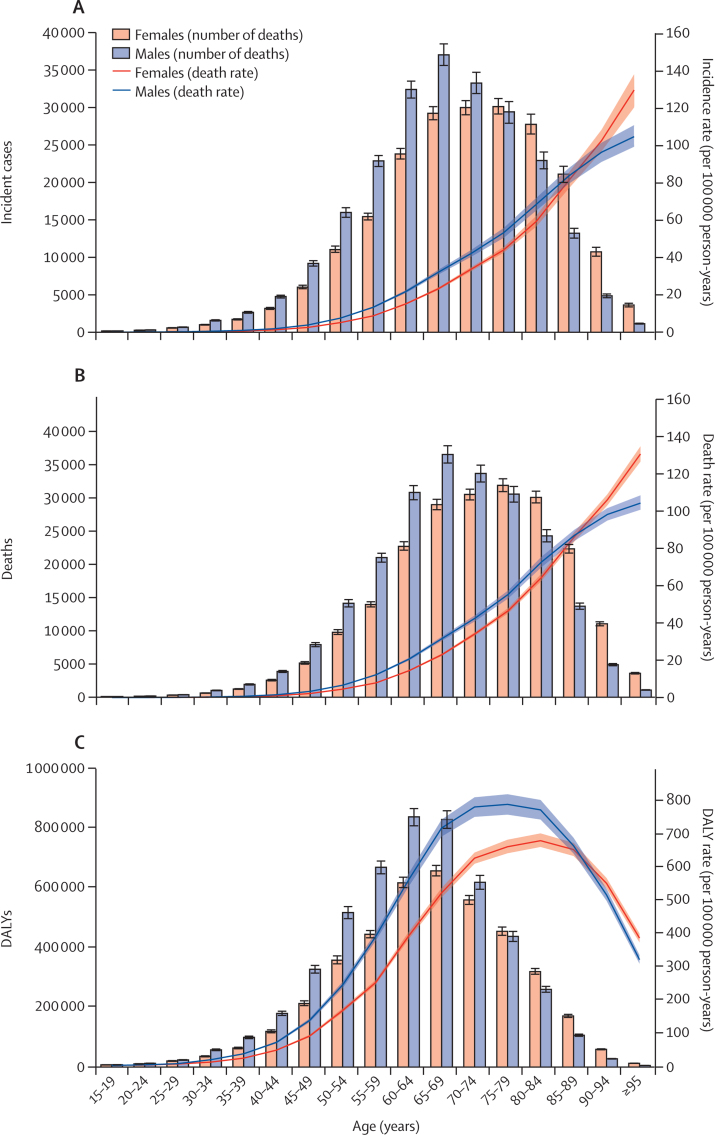
Age-specific counts and rates of incident cases (A), deaths (B), and DALYs (C) of pancreatic cancer by sex, 2017 DALYs=disability-adjusted life-years.

Age-specific rates for both incidence and deaths increased with increasing age; this trend was similar between males and females (figure 2A, B). The number of both deaths and incident cases peaked at the ages of 65–69 years in males, whereas the peak in females was observed at the ages of 75–79 years. Additionally, the numbers of deaths and incident cases were lower in females younger than 75 years than in males in the same age group, whereas the numbers were higher in females than in males in age groups of 75 years and older (figure 2A, B). Until the ages of 90–94 years, the incidence, death, and DALYs rates were higher in males than in females in the same age group (figure 2). The age pattern for number of DALYs showed a similar trend to number of deaths and incident cases for total counts, but the rates decreased in age groups older than 80 years. Similar to the number of incident cases and deaths, in 2017 the number of DALYs was much higher in males than in females in all age groups younger 75 years, after which female DALY numbers were higher (although with overlapping uncertainty in the age group of 75–79 years).

**Figure 3:**
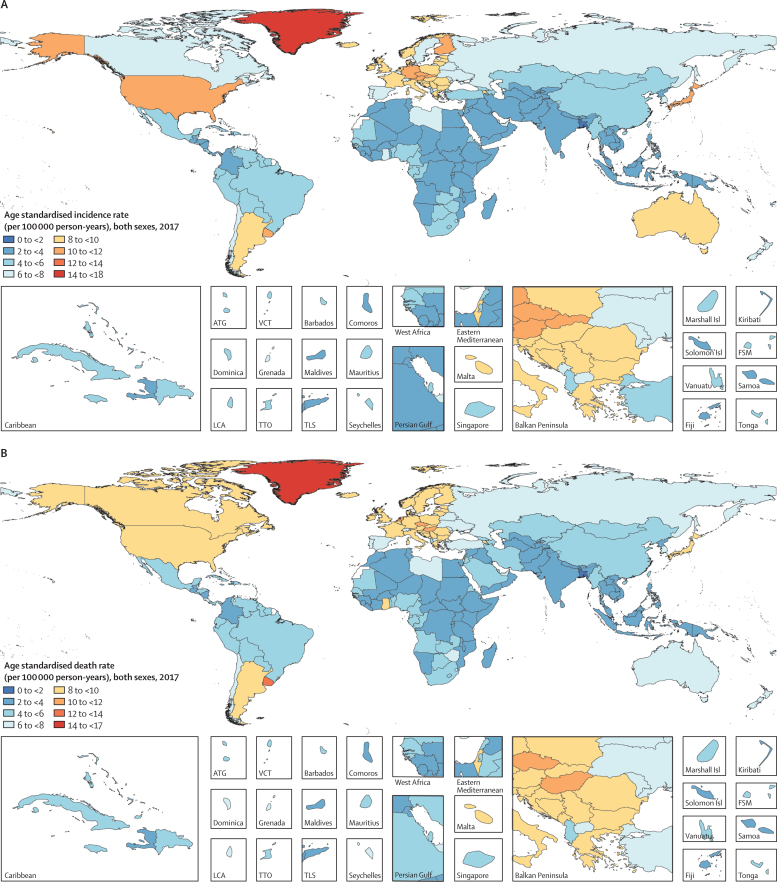
Age-standardised rates of incidence (A) and death (B) of pancreatic cancer across 195 countries and territories in both sexes, 2017

In both 1990 and 2017, the highest age-standardised death rates were observed in Greenland: 19·7 (95% UI 17·8–21·8) per 100 000 person-years in 1990 and 17·4 (15·8–19·0) per 100 000 person-years in 2017 (figure 3A; appendix p 11). Yet the number of deaths due to pancreatic cancer in Greenland was among the lowest in the world (11·1 [10·2–12·1] in 2017). Uruguay was the next leading country for highest age-standardised death rates from pancreatic cancer, although it was substantially behind Greenland, with an age-standardised death rate of 12·1 (10·9–13·5) per 100 000 person-years in 2017. Bangladesh (1·9 [1·5–2·3] per 100 000 person-years) had the lowest age-standardised rate in 2017, whereas São Tomé and Príncipe (1·3 [1·1–1·5] per 100 000 person-years) had the lowest rate in 1990. The incidence rates followed a very similar pattern: highest in Greenland in both 1990 and 2017, lowest in São Tomé and Príncipe in 1990, and lowest in Bangladesh in 2017 (figure 3B). All estimates were similar between males and females (data not shown). Specific country and territory data for incidence, deaths, and DALYs can be found in the appendix (pp 11–34).

The average annualised percentage change in both age-standardised incidence and death rates was highest in Grenada (5·5%) and lowest in Bahrain (−1·2%) from 1990 to 2017. For males, the percentage change in both age-standardised incidence and death rates was highest in Bermuda (6·0%) and lowest in Qatar (−1·5% for age-standardised incidence rate and −1·6% for age-standardised death rate). For females, the highest percentage change in both age-standardised incidence and death rates was in Grenada (5·4% for age-standardised incidence rate and 5·5% for age-standardised death rate) and the lowest in Bahrain (−1·2%).

**Figure 4:**
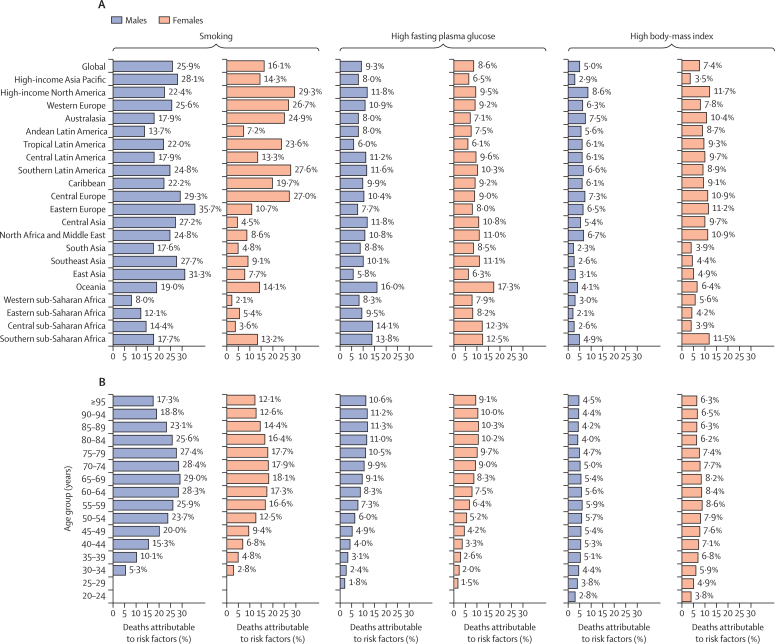
Fraction of pancreatic cancer age-standardised deaths attributable to smoking, high fasting plasma glucose, and high body-mass index by region (A) and fraction of pancreatic cancer age-specific deaths attributable to smoking, high fasting plasma glucose, and high body-mass index by age group (B) for males and females, 2017

93 600 (95% UI 82 500–108 000) pancreatic cancer deaths, equivalent to 21·1% (18·8–23·7) of all age-standardised deaths from pancreatic cancer, were attributable to smoking for both sexes combined in 2017. The age-standardised proportions of all pancreatic cancer deaths that were attributable to smoking in 2017 were 25·9% (22·2–29·6) for males and 16·1% (13·2–18·8) for females (figure 4A). 59 000 (63·1%; 50 000–68 000) of these deaths occurred in males and 33 500 (36·1%; 28 000–41 000) in females. In 1990, the proportion of pancreatic cancer age-standardised deaths attributable to smoking was 26·6% (23·8–29·5) for both sexes combined.

Globally, in 2017, 8·9% (2·1–19·4) of pancreatic cancer age-standardised deaths were attributable to high fasting plasma glucose, including 9·3% (1·7–21·3) in males and 8·6% (1·4–19·6) in females (figure 4A; appendix pp 8–9), compared with 7·7% (1·8–16·8) for both sexes combined in 1990. Likewise, 6·2% (2·5–11·4) of pancreatic cancer age-standardised deaths were attributable to high BMI, including 5·0% (0·0–12·1) in males and 7·4% (2·6–13·0) in females (figure 4A; appendix pp 8–9), compared with 5·0% (1·9–9·6) for both sexes combined in 1990.

In 2017, the proportion of age-standardised deaths attributable to smoking for males was highest in eastern Europe (35·7% of all pancreatic cancer deaths) and east Asia (31·3%); for females it was highest in high-income North America (29·3%) and southern Latin America (27·6%). The lowest age-standardised attributable proportion for smoking was observed in western sub-Saharan Africa for both males (8·0%) and females (2·1%). In 2017, the highest proportion of age-standardised deaths attributable to high fasting plasma glucose was observed in Oceania in both males (16·0%) and females (17·3%), and the highest fraction attributable to high BMI was observed in high-income North America for both males (8·6%) and females (11·7%). Additionally, in 2017, the lowest proportion of age-standardised deaths attributable to high fasting plasma glucose was observed in east Asia in males (5·8%) and in tropical Latin America in females (6·1%). As for proportion of deaths attributable to high BMI, the lowest proportion in males was observed in eastern sub-Saharan Africa (2·1%), and in females the lowest proportion was observed in high-income Asia Pacific (3·5%; figure 4A).

Across age groups, the proportion of age-standardised deaths attributable to smoking was higher than 25% in males aged between 55 and 84 years and higher than 16% in females in the same age group (figure 4B). The highest proportion attributable to high fasting plasma glucose in both sexes was observed in the 85–89 year age group. Although higher proportions of pancreatic cancer deaths attributable to high BMI were observed between the ages of 45 years and 79 years, the proportions were more similar between all age groups than for the leading risk factors, with attributable deaths starting to occur at the ages of 20–24 years (figure 4B).

From 1990 to 2017, the age-standardised rates of both deaths and incidence of pancreatic cancer increased, along with increases in SDI. That is, the lowest rates were observed in low SDI countries and higher rates were detected in countries with respectively higher SDI across all years from 1990 to 2017 (appendix p 3).

**Figure 5:**
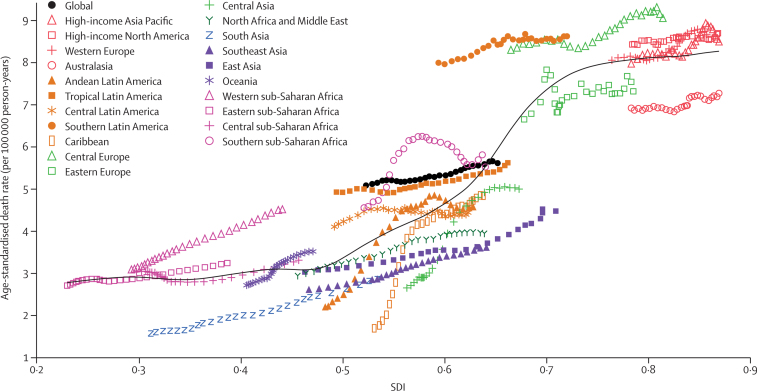
The trend in age-standardised death rates of pancreatic cancer across 21 GBD regions by SDI for both sexes combined, 1990–2017 For each region, points from left to right depict estimates from each year from 1990 to 2017. GBD=Global Burden of Diseases, Injuries, and Risk Factors Study. SDI=Socio-demographic Index.

**Figure 6:**
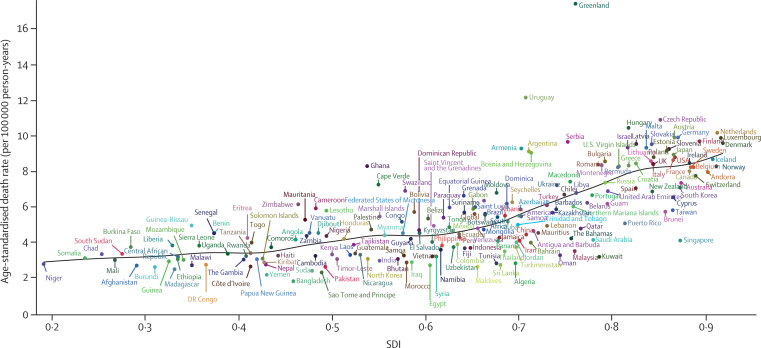
The age-standardised death rates of pancreatic cancer across 195 countries and territories by SDI in both sexes, 2017 SDI=Socio-demographic Index.

Figure 5 demonstrates the trend in age-standardised death rates across SDI by region, from 1990 to 2017. Regions generally followed the trend of increasing death, incidence, and DALY rates along with increases in SDI (figure 5; appendix pp 4–5). Several regions, including Andean Latin America and southern sub-Saharan Africa, showed a decrease in age-standardised death rate late in the study period, but not down to 1990 levels. Among high-income regions, Australasia had a rising age-standardised death rate but it was well below the expected levels in all years, while other high-income regions were either near or above the levels expected on the basis of SDI. Although south Asia, southeast Asia, and east Asia had rising age-standardised death rates, they were among the lowest in the world and were below the expected levels in all years from 1990 to 2017. Figure 6 demonstrates the association between age-standardised death rate and SDI across countries and territories in 2017. Similar to regional trends, there was a trend at the national level of increasing age-standardised death rates along with increases in SDI. As mentioned previously, the observed levels were much higher than expected in Greenland and Uruguay and much lower than expected in many countries, including Bangladesh, Kuwait, and Singapore based solely on SDI.

## Discussion

Our results showed that there were approximately 441 000 deaths due to pancreatic cancer worldwide in 2017. The incidence and death rates of pancreatic cancer for both sexes varied greatly across GBD regions. The 2017 age-standardised death rates for pancreatic cancer were highest in Greenland and Uruguay. The lowest were observed in Bangladesh and São Tomé and Príncipe. Although there was not a clear explanation for such great differences in pancreatic cancer mortality in different regions of the world, this national variation could be attributed to exposure to known or suspected lifestyle and environmental risk factors related to pancreatic cancer as well as scarcity of efficient diagnostic tools in low-income and middle-income countries.^14^, ^15^ The substantial increase in worldwide pancreatic cancer suggests that change in ageing populations, especially in low and middle SDI countries, and environmental and behavioural changes, more so than genetic factors, are related to its cause. Differences in pancreatic cancer death and incidence rates across countries could also reflect variation in quality of cancer registry data and tools for pancreatic cancer diagnosis.^16^, ^17^

Since 1990, regional age-standardised death and incidence rates of pancreatic cancer generally increased with increasing SDI. During the past three decades, these rates were consistently higher in high SDI regions and lower in low SDI regions. Higher incidence of pancreatic cancer in high SDI countries could be due to the ageing population and to lifestyle choices that increase exposure to risk factors; some of the risk factors for pancreatic cancer are more prevalent in high SDI countries than in low ones.^2^, ^18^

There was a 2·3 times increase in global number of incident cases and deaths of pancreatic cancer in both sexes from 1990 to 2017, reflecting both ageing and growth of the population, especially in low and middle SDI countries. The age-standardised death and incidence rates increased from 1990 to 2017 at the global level. This increase was probably related to an increase in the prevalence of obesity and diabetes, as reflected by the risk factors of high BMI and fasting plasma glucose, two of the known risk factors for pancreatic cancer.^15^, ^19^, ^20^ This increase occurred despite a mild-to-moderate reduction in smoking rates in high-income countries over the study period.^21^

The incidence and mortality of pancreatic cancer were somewhat higher in males than in females across age groups lower than 75 years in all years from 1990 to 2017; 51·9% of pancreatic cancer deaths occurred in males and 48·1% in females. This pattern is consistent with other worldwide studies.^3^, ^22^ The reasons for lower incidence of pancreatic cancer in females are not completely understood. Females are either less prone to pancreatic cancer or have less exposure to smoking as the main environmental risk factor of pancreatic cancer.^4^ Smoking in males (25·0%) is five times more frequent than in females (5·4%) worldwide.^21^

High-income regions had the highest death and incidence rates of pancreatic cancer for both sexes combined in all years from 1990 to 2017. Similar to other comparable studies, we found that pancreatic cancer is mainly a disease of high-income countries, where overall rates are nearly three times higher than in middle-income and low-income countries, but age-standardised rates are increasing at similar rates among countries with different SDI levels.^2^, ^22^ A forecasting study has predicted that pancreatic cancer will escalate from the fourth to the second leading cause of cancer deaths in the USA by 2030,^23^ and another study forecasted that 111 500 deaths from pancreatic cancer will occur in the European region by 2025, an almost 50% increase in the number of recorded deaths from pancreatic cancer in 2010.^24^, ^25^

Pancreatic cancer is typically a disease of older people, since 90% of newly diagnosed patients are aged older than 55 years, with most in their seventh and eighth decades of life.^3^, ^22^ Although pancreatic cancer rarely presents before the age of 45 years, the incidence rises sharply thereafter. As survival for chronic diseases improves, the number of older patients diagnosed with pancreatic cancer is increasing. Age is the most important risk factor in the development of pancreatic cancer. We found the number of both deaths and incident cases had a peak in the age group of 65–69 years in males, whereas the peak in females was observed in the age group of 75–79 years. Using a life table approach is necessary to assess differences in exposure and genetic susceptibility among males and females with pancreatic cancer. It has not been reported previously that females experience pancreatic cancer at older ages than males.

Pancreatic cancer remains one of the deadliest cancers, with a 5% 5-year survival rate.^22^ 5-year survival rates from pancreatic cancer have changed little over the past decades. The current rate of 5% is only slightly improved from 3% in 1970.^26^ Pancreatic cancer is often diagnosed at advanced stages and responds poorly to chemotherapy, leading to low treatment success rates.^22^

The estimated population attributable fraction of pancreatic cancer deaths to tobacco smoking is 11–32%.^27^ We found that the proportion of age-standardised deaths attributable to smoking decreased slightly from 1990 (26·6% [95% UI 23·8–29·5]) to 2017 (21·1% [18·8–23·7]) in both sexes combined, but it was still higher than the proportion of age-standardised deaths attributable to high fasting plasma glucose (8·9% [2·1–19·6]) and high BMI (6·2% [2·5–11·4]) in 2017. Reductions in the proportion of pancreatic cancer cases attributable to smoking are similar worldwide reductions in smoking rates. Globally, the age-standardised prevalence of daily smoking decreased by 28·4% for males and 34·4% for females between 1990 and 2015; however, four countries had significant annualised increases in smoking prevalence between 2005 and 2015 (Congo and Azerbaijan for males and Kuwait and Timor-Leste for females).^21^ On the basis of a 2012 study^28^ by the International Pancreatic Cancer Case-Control Consortium (PanC4; 6507 pancreatic cancer cases, 12 890 controls), former smokers, in comparison with never smokers, had an odds ratio (OR) of 1·2 (95% CI 1·0–1·3), and current smokers, in comparison with never smokers, had an OR of 2·2 (1·7–2·8), for risk of pancreatic cancer, with a trend of significantly increasing risk of pancreatic cancer with increasing number of cigarettes among current smokers (OR 3·4 for ≥35 cigarettes per day, p_trend_<0·0001). Risk increased in relation to duration of cigarette smoking up to 40 years of smoking (OR 2·4).^28^ No trend in risk was observed for age at starting cigarette smoking, whereas risk decreased with increasing time since cigarette cessation, with an OR of 0·98 after 20 years.^28^ We found that the highest proportion of pancreatic cancer deaths attributable to smoking for both sexes was observed in the 55–84 year age group, which is consistent with the findings from the PanC4.^28^ The International Agency for Research on Cancer confirmed that smoking is causally associated with pancreatic cancer.^29^

Type 2 diabetes has been linked with an excess risk of pancreatic cancer in several studies.^30^, ^31^, ^32^ We found that 8·8% of pancreatic cancer deaths were attributable to high fasting plasma glucose in both sexes in 2017. By comparison, a population study in Italy estimated that 9·7% of pancreatic cancer occurrence was attributable to diabetes.^33^ The US National Cancer Institute estimated that diabetes is associated with a 1·8 times increased risk of pancreatic cancer.^34^ From 1980 to 2014, in all countries, diabetes prevalence in adults either increased, especially in low and middle SDI locations, or at best remained unchanged; worldwide, the number of adults with diabetes has quadrupled,^19^ so it is expected that diabetes will have a greater contribution to pancreatic cancer occurrence in the future.

Large studies have indicated a positive association between increasing BMI and risk of pancreatic cancer.^18^, ^35^ A pooled study^35^ of seven prospective cohorts showed that compared with normal weight (BMI 18·5 to <25), the adjusted relative risk for pancreatic cancer was 1·13 for overweight (BMI 25 to <30 kg/m^2^) and 1·19 for obesity class I (BMI 30 to <35 kg/m^2^). A pooled analysis from the Pancreatic Cancer Cohort Consortium^18^ showed that in males, the adjusted OR for pancreatic cancer for the highest versus lowest quartile of BMI was 1·33, and in females it was 1·34. The prevalence of obesity (BMI ≥30 kg/m^2^) is increasing at an alarming rate in many parts of the world. The number of obese people has risen globally from 105 million in 1975 to 641 million in 2014. Since 1975, the prevalence of obese males has more than tripled, and that of obese females has more than doubled.^36^ More than 2 billion people are overweight, and a third of them are obese. By 2025, global obesity prevalence is projected to reach 18% in males and surpass 21% in females; severe obesity is likely to surpass 6% in males and 9% in females.^19^ We found that 6·2% of pancreatic cancer in both sexes (5·0% in males, 7·4% in females) was attributable to obesity, which is inconsistent with estimated population attributable fractions (3–16%).^27^ Although obesity carries a modest risk for pancreatic cancer, its rapid increase makes it a serious risk factor for pancreatic cancer, especially in females, who are more commonly obese than males.

Future strategies should include comprehensive policies to control tobacco use and reduce the burden of obesity and diabetes across the world. Additionally, efforts must be made to identify other modifiable risk factors for pancreatic cancer, such as opium use in North Africa and the Middle East.^37^, ^38^

This study has several limitations. Generally, as for estimation of all diseases and cancers in the GBD study, the major limitation of the current study is the lack of high-quality data in many regions and countries, particularly in low-income locations. Although we did a sensitive search to take advantage of all available data sources comprising cancer registries and vital registration systems, in many locations data were either sparse or entirely unavailable. For estimating pancreatic cancer burden in these locations, we had to base our estimations on covariates and spatiotemporal smoothing. Ascertainment bias, detection bias, and diagnostic inaccuracy were additional limitations in low-income locations. The reported high incidence and mortality of pancreatic cancer in high-income versus low-income locations might be partly related to availability of accurate diagnostic modalities for pancreatic cancer and richness of data in high-income regions. Additionally, all of the general limitations in estimating the burden attributable to risk factors through comparative risk assessment methods were challenging. Finally, because of lags in data availability, recent estimates of trends relied on covariates and past trends, leading to wider UIs.

Pancreatic cancer deaths and incidence more than doubled over the study period. Much of this increase was due to increases in population and longevity, but even after accounting for population changes, incidence and death rates increased from 1990 to 2017, probably due to changes in associated risk factors. Pancreatic cancer is an aggressive cancer, predicted to become the second leading cause of cancer deaths in some regions. It often presents in old age, at an advanced stage, and has a poor prognosis. Major risk factors associated with pancreatic cancer (smoking, diabetes, and obesity) are potentially modifiable, affording a unique opportunity for preventing one of the deadliest cancers. The results of our study can be used by policy makers to allocate resources efficiently for developing methods for early diagnosis of pancreatic cancer, reducing its modifiable risk factors, and evaluating novel treatment strategies to reduce its case-fatality rate by proper treatment strategies.

**This online publication has been corrected. The corrected version first appeared at thelancet.com/gastrohep on Feb 12, 2020**
