# Correction to *Lancet Gastroenterol Hepatol* 2019; 4: 934–47

**DOI:** 10.1016/S2468-1253(20)30018-2

**Published:** 2020-02-12

*GBD 2017 Pancreatic Cancer Collaborators. The global, regional, and national burden of pancreatic cancer and its attributable risk factors in 195 countries and territories, 1990–2017: a systematic analysis for the Global Burden of Disease Study 2017*. Lancet Gastroenterol Hepatol *2019; **4:** 934–47*—In this Article, the affiliation for C La Vecchia should read “Clinical Medicine and Community Health, University of Milan, Milano, Italy (Prof C La Vecchia MD)” and the affiliation for D B B Tadesse should read “Department of Nursing (D B B Tadesse), Aksum University, Aksum, Ethiopia (D B B Tadesse CAP)”. These changes have been made to the online version as of Feb 12, 2020.

