# Supplementary material for: The global, regional, and national burden of pancreatic cancer and its attributable risk factors in 195 countries and territories, 1990–2017: a systematic analysis for the Global Burden of Disease Study 2017
Source: Lancet Gastroenterol Hepatol. 2019 Oct 21;4(12):934–47. doi: 10.1016/S2468-1253(19)30347-4 (PMC7026711; doi:10.1016/S2468-1253(19)30347-4)
Supplement: Supplementary appendix [file mmc1.pdf]

THE LANCET
Gastroenterology & Hepatology

Supplementary appendix

This appendix formed part of the original submission and has been peer reviewed. We post it as supplied by the authors.

Supplement to: GBD 2017 Pancreatic Cancer Collaborators. The global, regional, and national burden of pancreatic cancer and its attributable risk factors in 195 countries and territories, 1990–2017: a systematic analysis for the Global Burden of Disease Study 2017. *Lancet Gastroenterol Hepatol* 2019; published online Oct 21. http://dx.doi.org/10.1016/S2468-1253(19)30347-4.

Supplementary appendix

Supplement to: The global, regional, and national burden of pancreatic cancer in 195 countries and territories, 1990–2017: a systematic analysis for the Global Burden of Disease Study 2017

Table of Contents:

Appendix Figures

Appendix Figure 1: The trend of age-standardised rates of deaths (a) and incidence (b) due to pancreatic cancer across Global Burden of Disease super-regions from 1990 to 2017 in both sexes.

Appendix Figure 2: The age-standardised rates of (a) deaths and (b) incidence due to pancreatic cancer across SDI quintiles from 1990 to 2017 in both sexes. SDI=Socio-demographic Index.

Appendix Figure 3: The age-standardised incidence rates of pancreatic cancer across 21 regions by SDI in both sexes in 2017. SDI=Socio-demographic Index.

Appendix Figure 4: The age-standardised DALY rates of pancreatic cancer across 21 regions by SDI in both sexes in 2017. DALYs=disability-adjusted life-years. SDI=Socio-demographic Index.

Appendix Figure 5: The age-standardised DALY rates of pancreatic cancer across 195 countries and territories by SDI in both sexes in 2017. DALYs=disability-adjusted life-years. SDI: Socio-Demographic Index.

Appendix Figure 6: Global number of YLLs and YLDs and age-standardised rate of YLLs and YLDs per 100 000 due to pancreatic cancer by age, 2017; Dotted and dashed lines indicate 95% upper and lower uncertainty intervals, respectively. YLDs=years lived with disability. YLLs=years of life lost.

Appendix Figure 7: Fraction and UIs of pancreatic cancer age-standardised deaths attributable to smoking, high fasting plasma glucose, and high body-mass index (a) by GBD region, and (b) for males and females, 2017. A supplement to figure 4 in the manuscript, which does not include UIs. GBD=Global Burden of Disease. UI=uncertainty interval.

Appendix Tables

Appendix Table 1: Sequelae for pancreatic cancer and associated disability weights from GBD 2017. GBD=Global Burden of Disease.

Appendix Table 2: Deaths from pancreatic cancer in 1990 and 2017 for both sexes and percentage change of age-standardised rates by location.

Appendix Table 3: Incident cases of pancreatic cancer in 1990 and 2017 for both sexes and percentage change of age-standardised rates by location.

Appendix Table 4: DALYs from pancreatic cancer in 1990 and 2017 for both sexes and percentage change of age-standardised rates by location. DALYs=disability-adjusted life-years.

Appendix Figure 1: The trend of age-standardised rates of deaths (a) and incidence (b) due to pancreatic cancer across Global Burden of Disease super-regions from 1990 to 2017 in both sexes.

Appendix Figure 1a

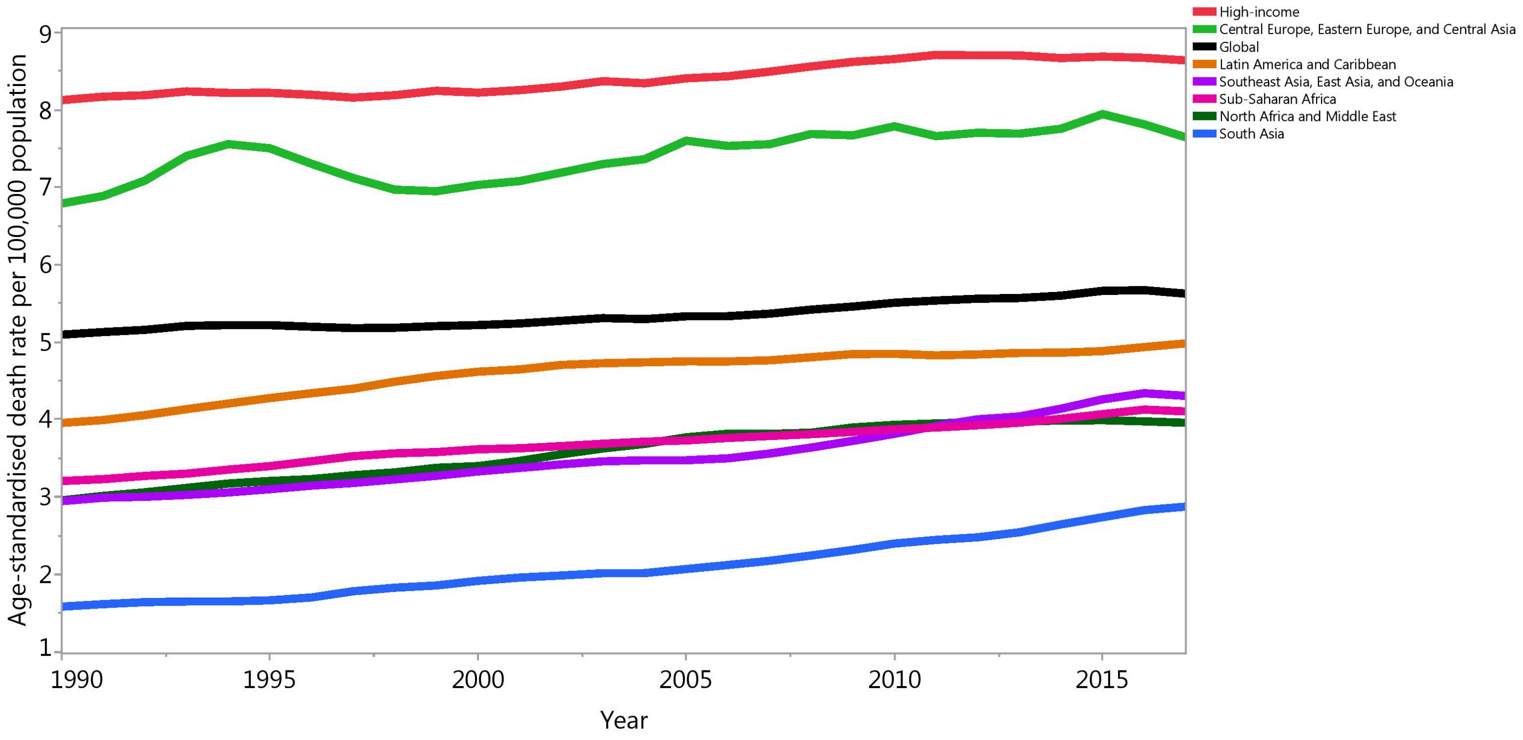

Appendix Figure 1b

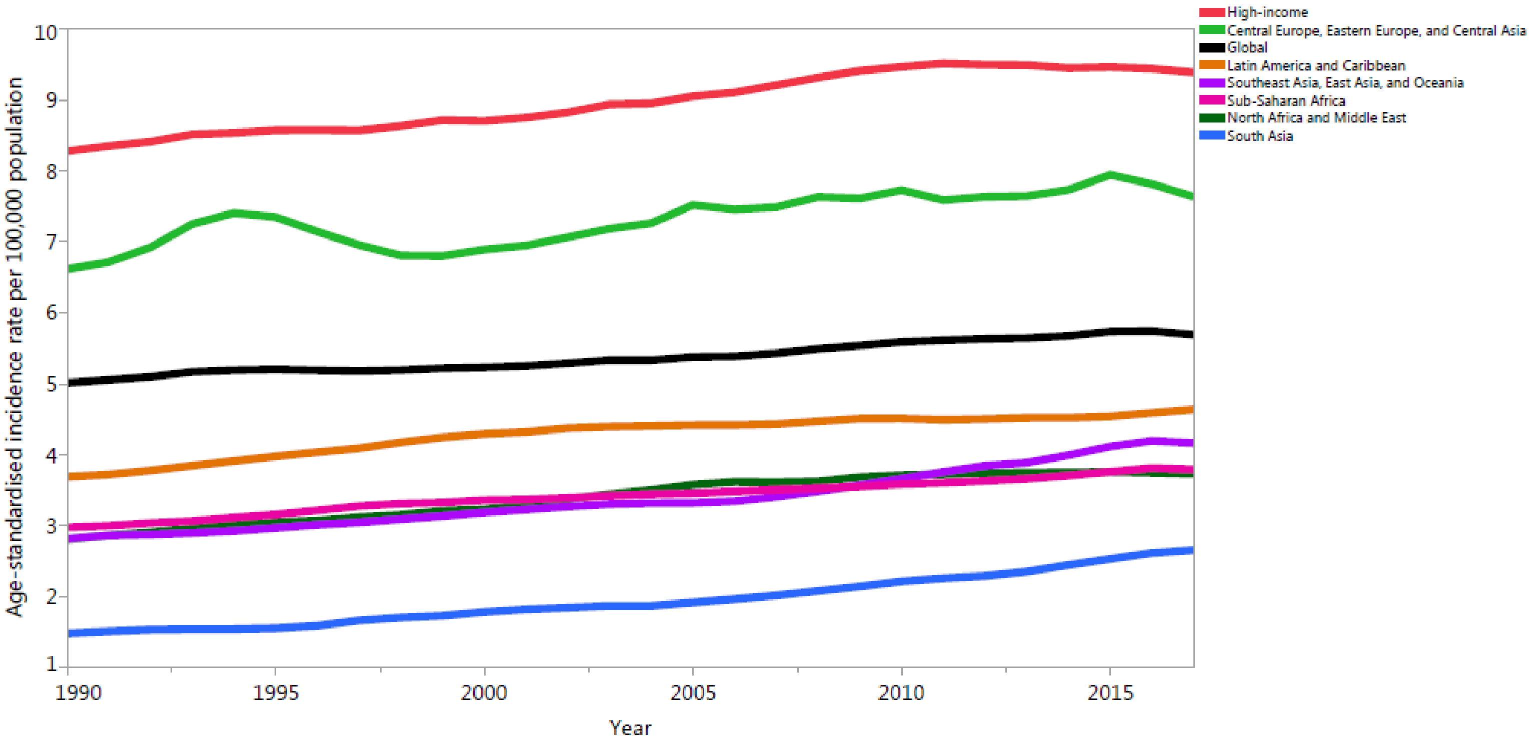

Appendix Figure 2: The age-standardised rates of a) deaths and b) incidence due to pancreatic cancer across SDI quintiles from 1990 to 2017 in both sexes. SDI=Socio-demographic Index.

Figure 2a

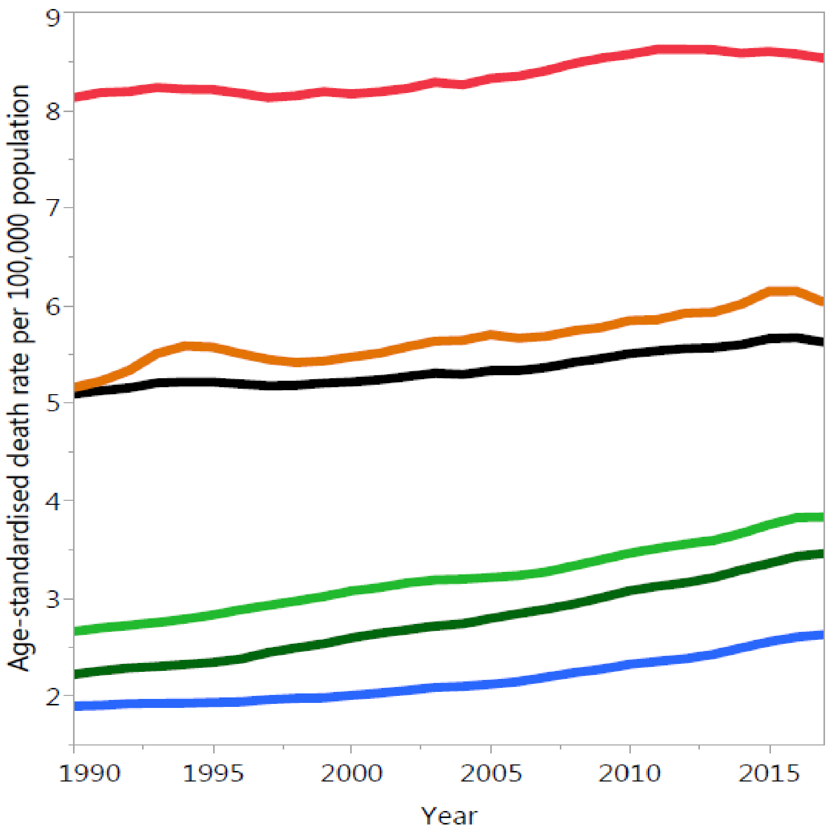

Figure 2b

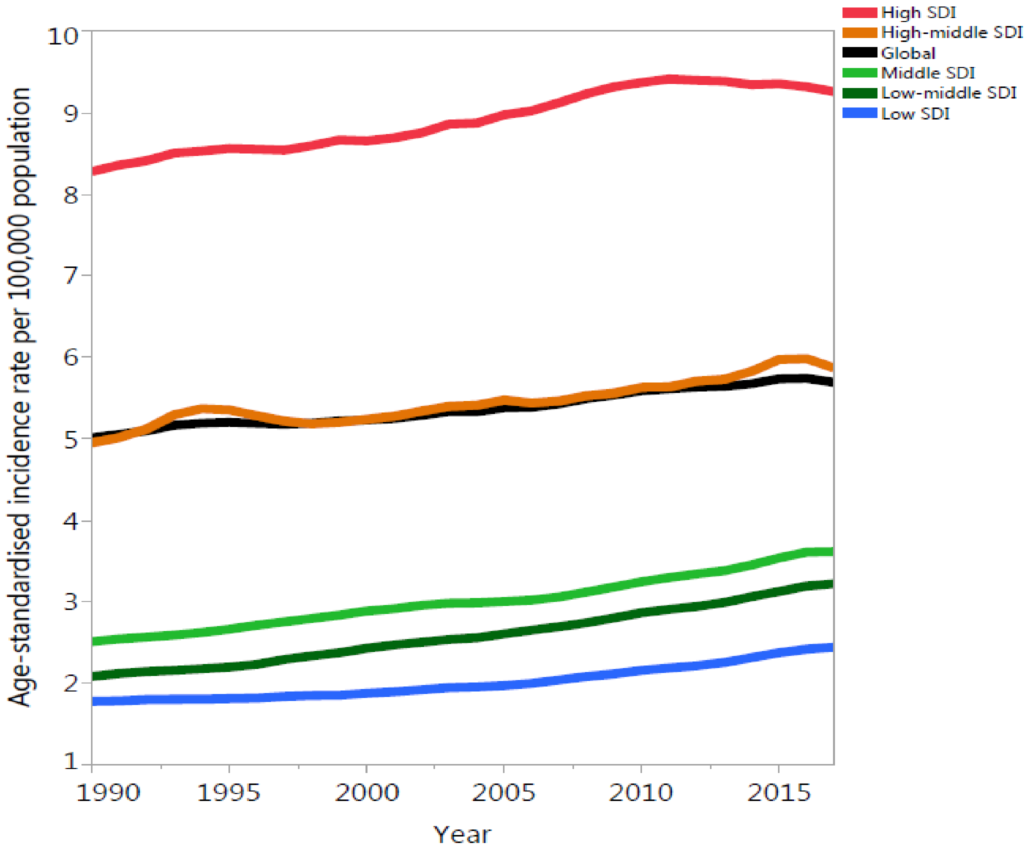

Appendix Figure 3: The age-standardised incidence rates of pancreatic cancer across 21 regions by SDI in both sexes in 2017. SDI=Socio-demographic Index.

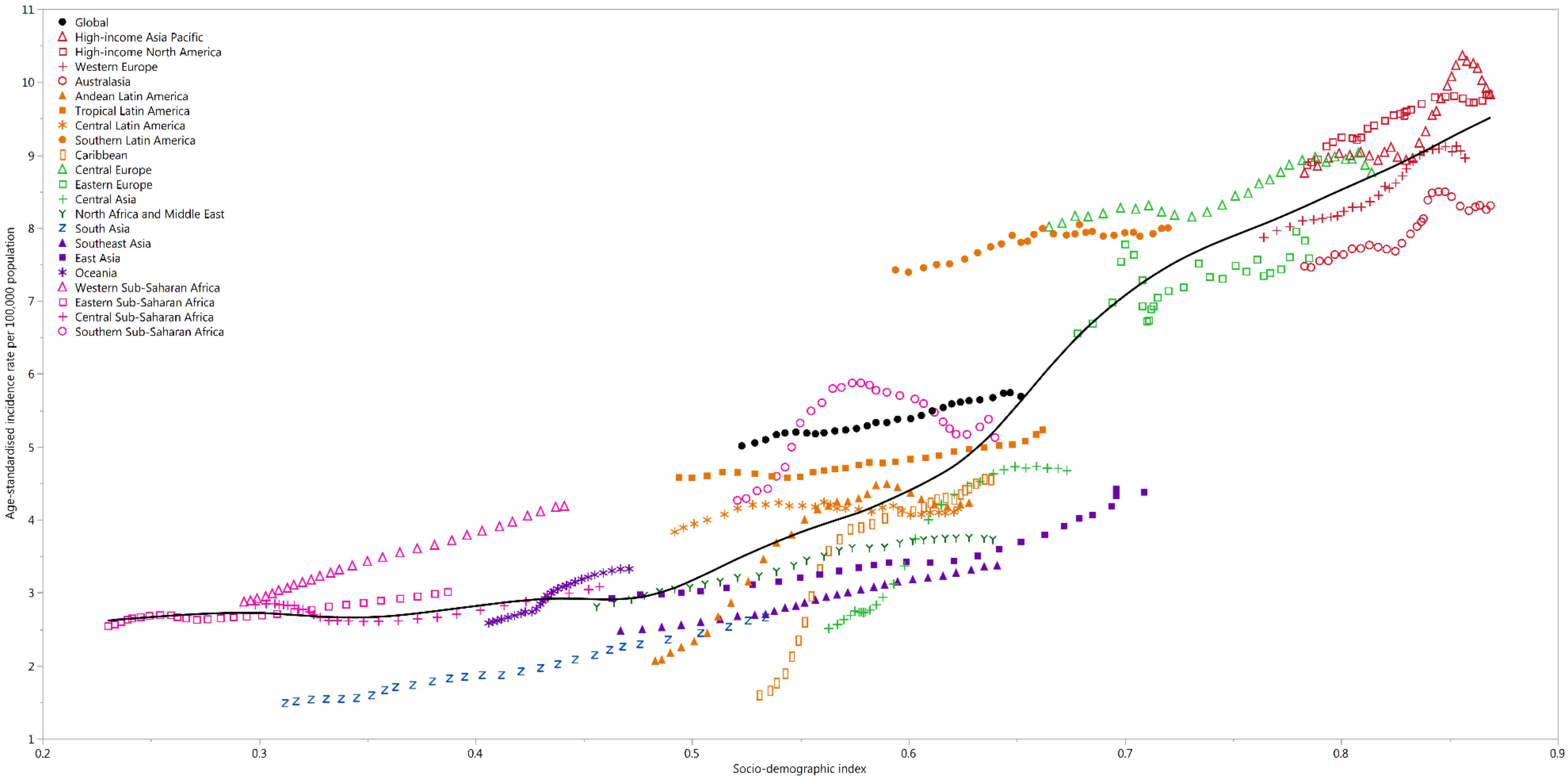

Appendix Figure 4: The age-standardised DALY rates of pancreatic cancer across 21 regions by SDI in both sexes in 2017. DALYs=disability-adjusted life-years. SDI=Socio-demographic Index.

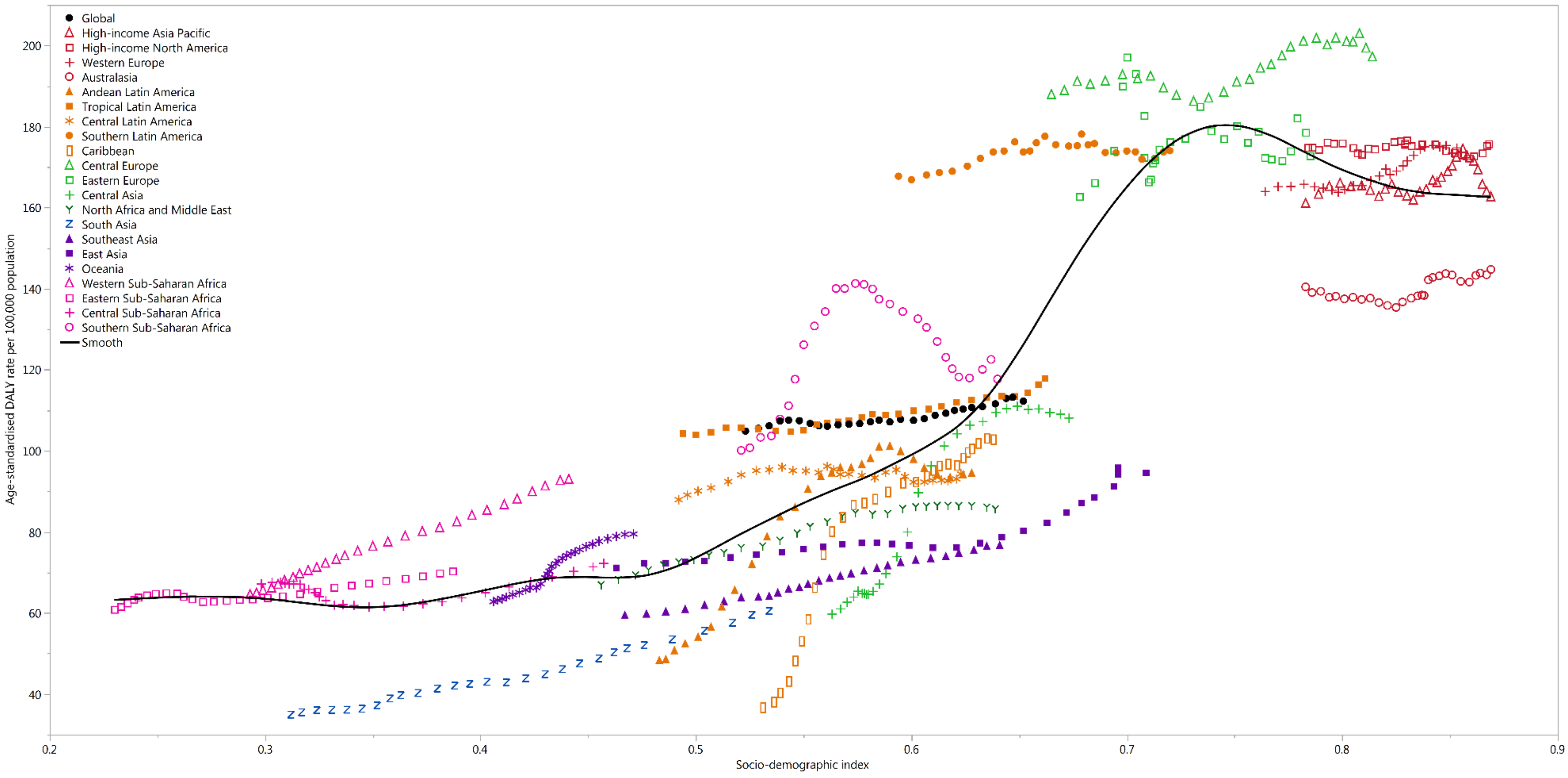

Appendix Figure 5: The age-standardised DALY rates of pancreatic cancer across 195 countries and territories by SDI in both sexes in 2017. DALYs=disability-adjusted life-years. SDI: Socio-demographic Index.

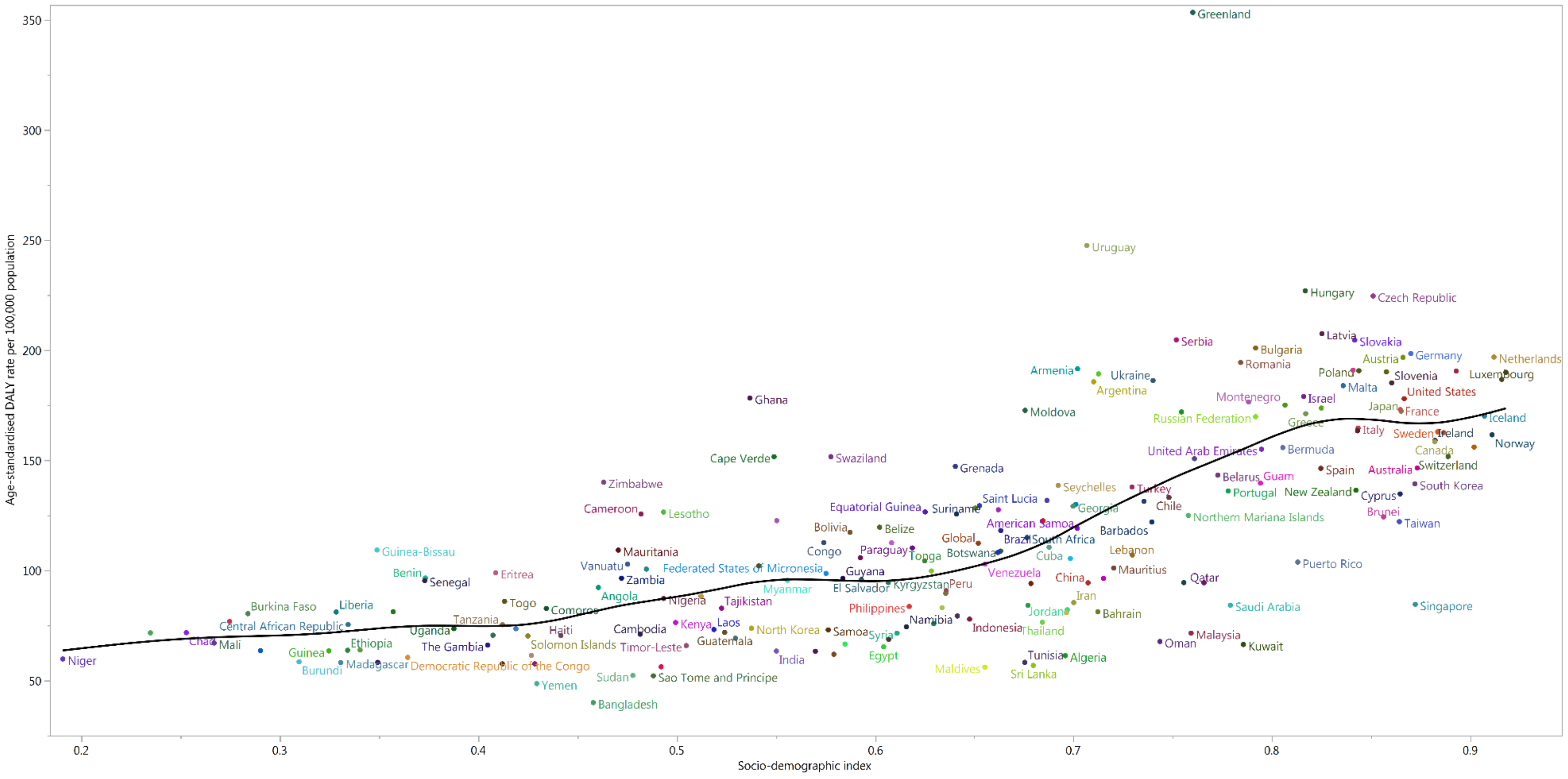

Appendix Figure 6: Global number of YLLs and YLDs and age-standardised rate of YLLs and YLDs per 100 000 due to pancreatic cancer by age, 2017; Dotted and dashed lines indicate 95% upper and lower uncertainty intervals, respectively. YLDs=years lived with disability. YLLs=years of life lost.

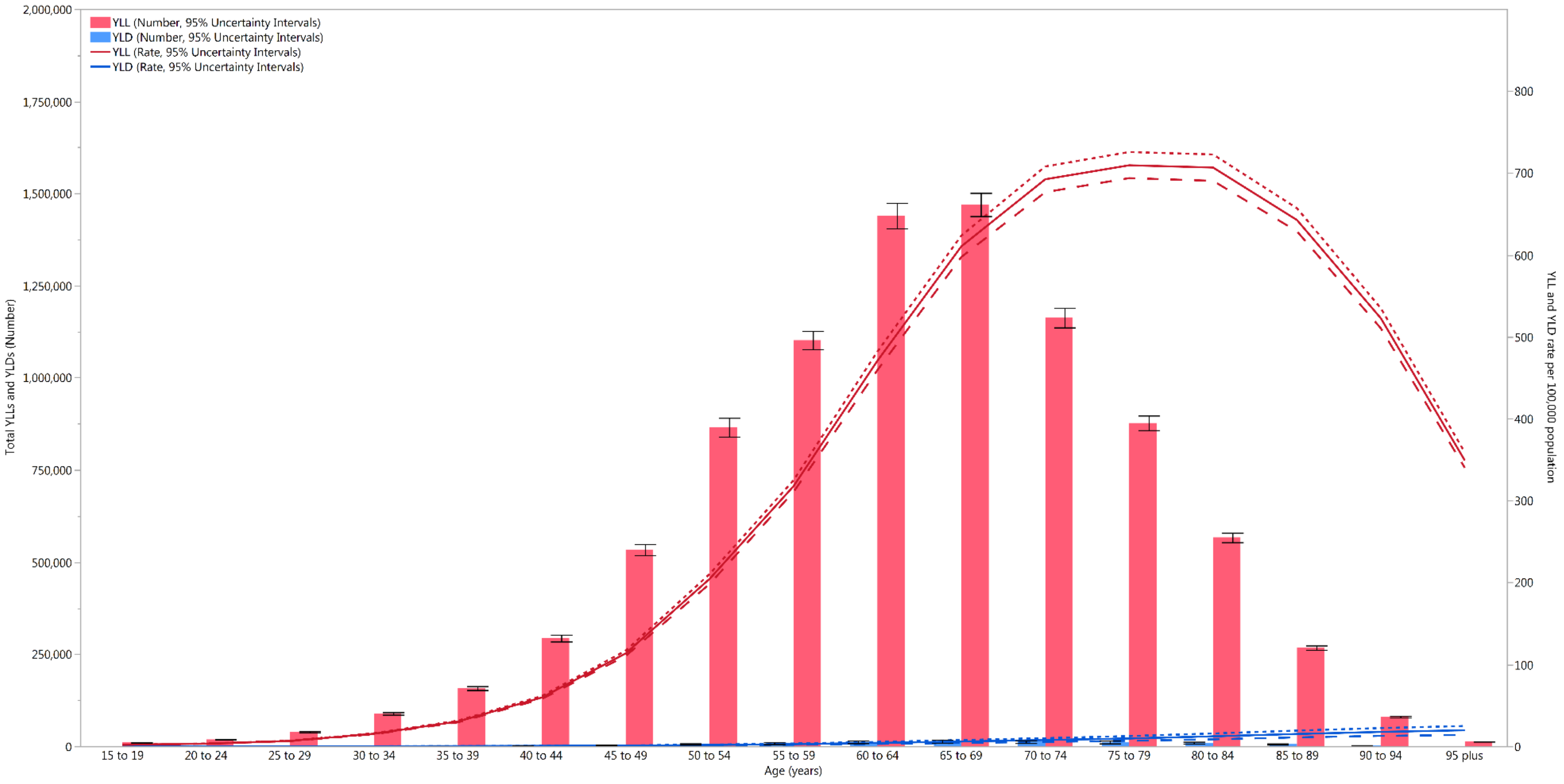

Appendix Figure 7a: Fraction and UIs of pancreatic cancer age-standardised deaths attributable to smoking, high fasting plasma glucose, and high body-mass index by GBD region, 2017. A supplement to figure 4a in the manuscript, which does not include UIs. GBD=Global Burden of Disease. UI=uncertainty interval.

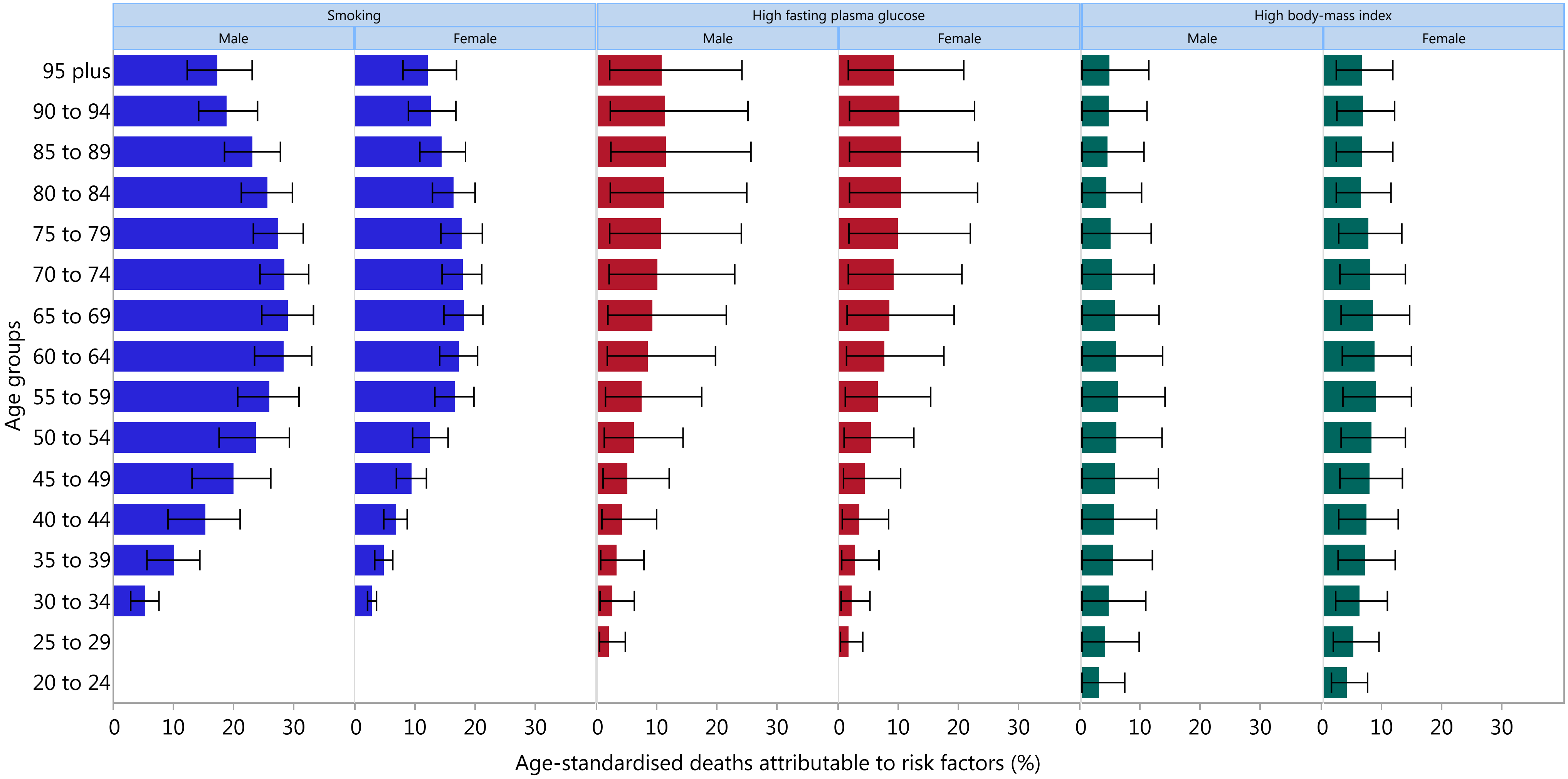

Appendix Figure 7b: Fraction and UIs of pancreatic cancer age-standardised deaths attributable to smoking, high fasting plasma glucose, and high body-mass index, for males and females, 2017. A supplement to figure 4b in the manuscript, which does not include UIs. UI=uncertainty interval.

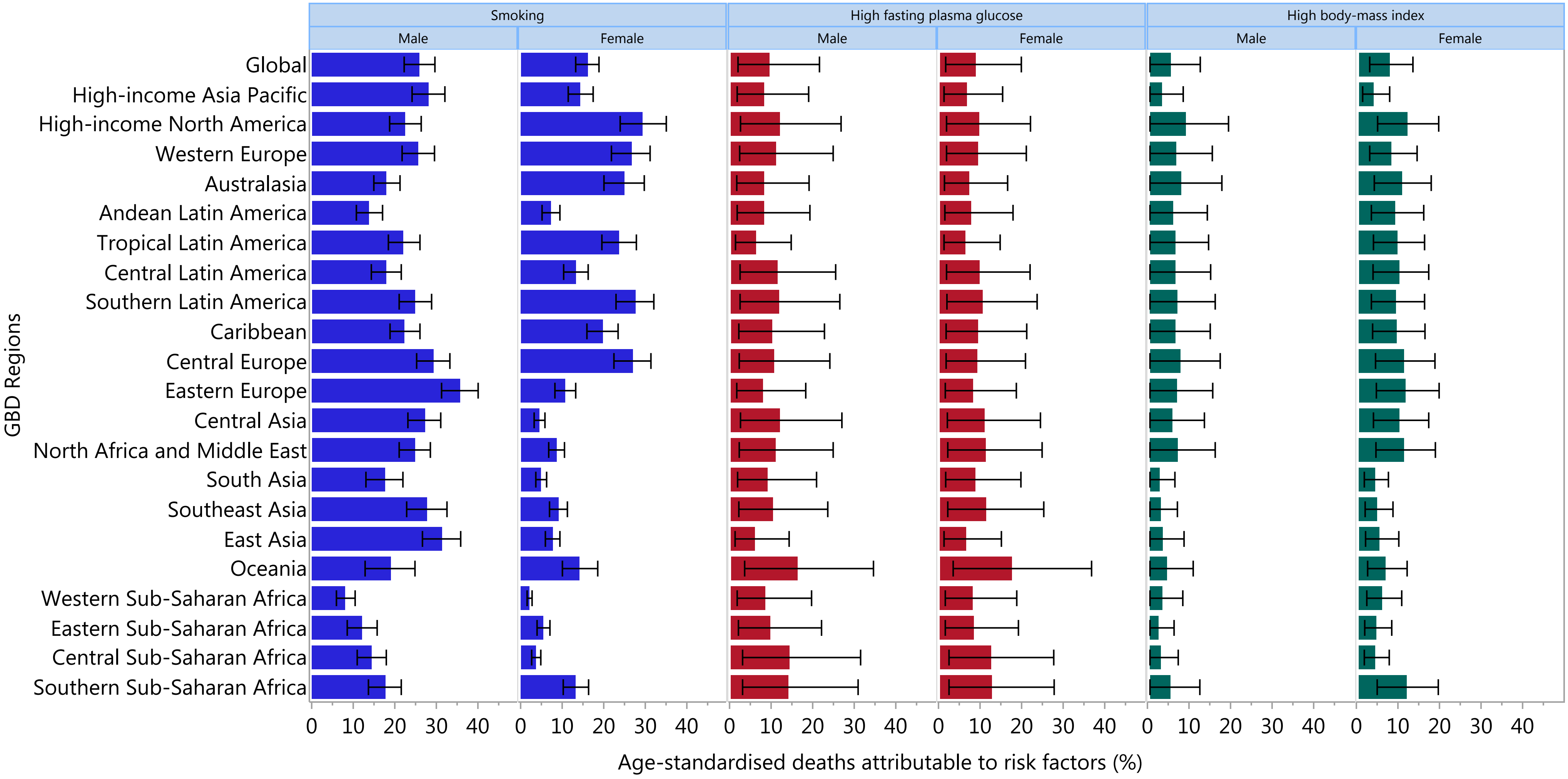

Appendix Table 1: Sequelae for pancreatic cancer and associated disability weights from GBD 2017

Sequela	Health state name	Lay description	Disability weight (95% UI)
Diagnosis and primary therapy phase	Cancer, diagnosis and primary therapy	Has pain, nausea, fatigue, weight loss, and high anxiety.	0.288 (0.193–0.399)
Controlled phase	Generic uncomplicated disease: worry and daily medication	Has a chronic disease that requires medication every day and causes some worry but minimal interference with daily activities.	0.049 (0.031–0.072)
Metastatic phase	Cancer, metastatic	Has severe pain, extreme fatigue, weight loss, and high anxiety.	0.451 (0.307–0.600)
Terminal phase	Terminal phase, with medication (for cancers, end-stage kidney/liver disease)	Has lost a lot of weight and regularly uses strong medication to avoid constant pain. The person has no appetite, feels nauseated, and needs to spend most of the day in bed.	0.540 (0.377–0.687)

GBD=Global Burden of Disease.

Appendix Table 2: Deaths from pancreatic cancer in 1990 and 2017 for both sexes and percentage change of age-standardised rates by location

	1990		2017		Percentage change in age-standardised rates between 1990 and 2017 (95% UI)
	Counts (95% UI)	Rate (95% UI)	Counts (95% UI)	Rate (95% UI)	
**Global**	195861 (192966, 199542)	5·1 (5, 5·2)	441083 (432833, 448960)	5·6 (5·5, 5·7)	10·4 (7·0, 13·0)
**High SDI**	106362 (105544, 107136)	8·1 (8·0, 8·2)	196275 (192396, 200659)	8·5 (8·4, 8·7)	5·0 (2·8, 7·4)
**High-middle SDI**	47566 (46099, 48993)	5·2 (5·1, 5·3)	106870 (103691, 110011)	6·0 (5·9, 6,2)	16·9 (11·8, 21·7)
**Middle SDI**	23995 (23326, 24700)	2·7 (2·6, 2·8)	81186 (77506, 84307)	3·8 (3·7, 4·0)	43·9 (36·0, 50·6)
**Low-middle SDI**	11651 (10829, 12592)	2·2 (2·1, 2·3)	38096 (36086, 40853)	3·5 (3·3, 3·7)	55·8 (41·8, 68·1)
**Low SDI**	5760 (5000, 7106)	1·9 (1·6, 2·3)	16979 (15706, 18236)	2·6 (2·4, 2·8)	38·7 (19·0, 57·5)
**High-income North America**	31020 (30681, 31305)	8·4 (8·3, 8·5)	54590 (53258, 56194)	8·7 (8·5, 9·0)	3·2 (0·3, 6·9)
**Canada**	3017 (2946, 3088)	9 (8·8, 9·2)	5542 (5152, 5950)	8 (7·5, 8·6)	-11·2 (-17·4, -4·5)
**Greenland**	6 (6, 7)	19·7 (17·8, 21·8)	11 (10, 12)	17·4 (15·8, 19)	-12·1 (-23·1, 1·5)
**USA**	27996 (27673, 28257)	8·4 (8·3, 8·5)	49036 (47847, 50521	8·8 (8·6, 9·1)	4·8 (1·7, 8·7)
**Australasia**	1678 (1641, 1716)	6·9 (6·8, 7·1)	3618 (3329, 3918)	7·3 (6·7, 7·9)	5·1 (-4, 14·4)
**Australia**	1400 (1365, 1434)	7 (6·8, 7·1)	3082 (2797, 3387)	7·4 (6·,7, 8·1)	5·8 (-4·5, 16·9)
**New Zealand**	278 (267, 291)	6·8 (6·6, 7·1)	536 (500, 577)	6·9 (6·4, 7·4)	0·7 (-6·7, 8·7)
**High-income Asia-Pacific**	16064 (15848, 16273)	8 (7·9, 8·1)	40359 (39113, 41673)	8·5 (8·2, 8·8)	6·6 (3·1, 10·4)
**Brunei**	4 (4, 5)	4·7 (4·1, 5·4)	16 (15, 18)	5·8 (5·2, 6·4)	22·7 (0·8, 44·8)
**Japan**	14001 (13822, 14185)	8·1 (8, 8·2)	34050 (33053, 35145)	8·9 (8·6, 9·3)	9·8 (6·4, 13·6)
**Singapore**	80 (76, 85)	3·6 (3·5, 3·8)	279 (257, 305)	4·1 (3·8, 4·5)	13·6 (3·2, 26)
**South Korea**	1978 (1911, 2046)	6·8 (6·5, 7)	6014 (5562, 6527)	7·1 (6·6, 7·7)	4·8 (-3·7, 14·1)
**Western Europe**	48775 (48267, 49311)	8·1 (8, 8·1)	81449 (78708, 84439)	8·7 (8·4, 9)	7·6 (3·8, 11·8)
**Andorra**	5 (4, 6)	8·1 (6·3, 9·6)	11 (8, 13)	8 (6·1, 9·7)	-1·4 (-20·7, 21·2)
**Austria**	1136 (1101, 1172)	9·2 (8·9, 9·5)	1809 (1695, 1941)	9·9 (9·3, 10·6)	7·8 (0·2, 16·3)
**Belgium**	1372 (1326, 1422)	8·5 (8·3, 8·8)	1951 (1812, 2102)	8·2 (7·6, 8·9)	-3·3 (-10·7, 4·6)
**Cyprus**	41 (36, 47)	4·8 (4·3, 5·5)	126 (113, 141)	6·6 (5·9, 7·4)	37·5 (11·4, 61·2)
**Denmark**	748 (722, 773)	8·8 (8·5, 9·1)	1117 (1033, 1199)	9·6 (8·9, 10·3)	8·3 (-0·3, 17·8)
**Finland**	721 (700, 744)	9·8 (9·5, 10·1)	1211 (1128, 1297)	9·7 (9, 10·4)	-1·1 (-8·6, 7·3)
**France**	6406 (6229, 6595)	7·4 (7·2, 7·6)	12271 (11419, 13173)	8·6 (8, 9·3)	16·7 (7·7, 26·8)
**Germany**	11561 (11224, 11902)	8·7 (8·5, 8·9)	19202 (17267, 21195)	9·9 (8·9, 10·9)	13·6 (1·8, 26·5)
**Greece**	1064 (1021, 1105)	6·8 (6·5, 7)	2049 (1912, 2187)	8·3 (7·8, 8·9)	23 (14·1, 32·1)

**Iceland**	26<br>(25, 28)	8·9<br>(8·4, 9·4)	47<br>(44, 51)	8·7<br>(8·1, 9·3)	-2·3<br>(-10·7, 6·3)
**Ireland**	401<br>(386, 416)	9·5<br>(9·2, 9·8)	627<br>(571, 684)	8·5<br>(7·7, 9·2)	-10·7<br>(-18·9, -1·9)
**Israel**	432<br>(411, 453)	8·7<br>(8·3, 9·1)	1074<br>(995, 1161)	9·3<br>(8·6, 10·1)	7·5<br>(-0·4, 16·8)
**Italy**	7658<br>(7461, 7873)	8·3<br>(8·1, 8·5)	12681<br>(11784, 13695)	8·4<br>(7·8, 9·1)	1·2<br>(-6·7, 9·6)
**Luxembourg**	50<br>(47, 53)	8·9<br>(8·4, 9·4)	98<br>(85, 110)	9·9<br>(8·5, 11·1)	11·5<br>(-4·5, 25·9)
**Malta**	35<br>(33, 37)	8·1<br>(7·6, 8·6)	85<br>(79, 92)	9·3<br>(8·6, 10)	14·8<br>(4·5, 25·9)
**Netherlands**	1791<br>(1742, 1843)	8·7<br>(8·4, 8·9)	3508<br>(3280, 3739)	10·2<br>(9·5, 10·8)	17·3<br>(9·3, 26)
**Norway**	598<br>(587, 609)	8·4<br>(8·3, 8·6)	801<br>(771, 836)	8·3<br>(8, 8·7)	-1·4<br>(-5·5, 3·7)
**Portugal**	951<br>(918, 985)	6·8<br>(6·6, 7·1)	1639<br>(1516, 1769)	6·7<br>(6·2, 7·2)	-2·4<br>(-10·3, 6·3)
**Spain**	3557<br>(3466, 3663)	6·3<br>(6·1, 6·4)	6930<br>(6480, 7402)	7·1<br>(6·6, 7·6)	12·9<br>(4·9, 21·5)
**Sweden**	1399<br>(1363, 1439)	8·8<br>(8·6, 9)	1770<br>(1674, 1874)	8·2<br>(7·8, 8·7)	-6<br>(-11·7, 0·1)
**Switzerland**	895<br>(860, 929)	8·2<br>(7·9, 8·5)	1363<br>(1268, 1469)	7·8<br>(7·2, 8·4)	-5·2<br>(-13·1, 3·5)
**United Kingdom**	7881<br>(7779, 7980)	8·3<br>(8·1, 8·4)	10991<br>(10771, 11274)	8·4<br>(8·3, 8·6)	2·1<br>(-0·3, 5·1)
**Southern Latin America**	3726<br>(3531, 3925)	8<br>(7·6, 8·4)	7185<br>(6658, 7754)	8·6<br>(8, 9·3)	7·9<br>(-1·6, 17·5)
**Argentina**	2721<br>(2534, 2918)	8·3<br>(7·8, 8·9)	4941<br>(4465, 5493)	9·1<br>(8·2, 10·1)	9·4<br>(-3·4, 23)
**Chile**	620<br>(588, 652)	6·3<br>(6, 6·6)	1565<br>(1405, 1746)	6·7<br>(6, 7·5)	6·9<br>(-5·1, 19·7)
**Uruguay**	385<br>(364, 410)	9·6<br>(9, 10·1)	679<br>(609, 754)	12·1<br>(10·9, 13·5)	26·8<br>(11·7, 43·2)
**Eastern Europe**	19172<br>(18023, 20470)	6·7<br>(6·3, 7·1)	25356<br>(24870, 25886)	7·3<br>(7·2, 7·5)	10<br>(3·9, 16·9)
**Belarus**	742<br>(700, 786)	5·6<br>(5·3, 5·9)	1001<br>(908, 1116)	6·2<br>(5·7, 7)	12·4<br>(1·9, 24·9)
**Estonia**	182<br>(174, 191)	8·6<br>(8·2, 9)	251<br>(220, 285)	9·2<br>(8, 10·4)	6<br>(-8·1, 21·1)
**Latvia**	334<br>(318, 351)	8·9<br>(8·5, 9·4)	393<br>(349, 440)	9·5<br>(8·5, 10·7)	6·6<br>(-5·6, 20·1)
**Lithuania**	382<br>(365, 398)	8·1<br>(7·8, 8·5)	502<br>(468, 540)	8·6<br>(8, 9·2)	5·3<br>(-3·2, 14·2)
**Moldova**	287<br>(268, 309)	6·3<br>(5·9, 6·7)	389<br>(364, 415)	6·8<br>(6·4, 7·3)	8·3<br>(-0·1, 17·9)
**Russia**	13591<br>(12938, 14180)	7·3<br>(7, 7·6)	17309<br>(17020, 17588)	7·4<br>(7·2, 7·5)	0·6<br>(-3·4, 6·5)
**Ukraine**	3653<br>(3159, 4399)	5<br>(4·3, 5·9)	5510<br>(5152, 5886)	7·2<br>(6·8, 7·7)	46·1<br>(22·2, 68·2)
**Central Europe**	12524<br>(12279, 12726)	8·3<br>(8·1, 8·4)	19596<br>(19036, 20168)	9·1<br>(8·8, 9·3)	9·1<br>(5·4, 12·8)
**Albania**	65<br>(60, 71)	3<br>(2·8, 3·3)	234<br>(193, 281)	5·5<br>(4·6, 6·6)	82·3<br>(45·4, 127)
**Bosnia and Herzegovina**	280<br>(265, 296)	7<br>(6·6, 7·4)	546<br>(498, 598)	9<br>(8·3, 9·9)	29<br>(16·7, 41·7)
**Bulgaria**	764<br>(737, 793)	5·9<br>(5·7, 6·1)	1262<br>(1176, 1355)	8·6<br>(8, 9·2)	44·3<br>(33·4, 55·2)
**Croatia**	524<br>(504, 543)	8·1<br>(7·8, 8·4)	762<br>(711, 818)	8·4<br>(7·9, 9·1)	4·2<br>(-3·5, 12·3)
**Czech Republic**	1650<br>(1599, 1695)	11·7<br>(11·4, 12)	2306<br>(2153, 2465)	10·9<br>(10·2, 11·6)	-7·2<br>(-13·9, 0)

**Hungary**	1496<br>(1450, 1541)	9·9<br>(9·6, 10·2)	2032<br>(1906, 2161)	10·4<br>(9·8, 11·1)	5·6<br>(-1·9, 12·9)
**Macedonia**	104<br>(97, 112)	5·6<br>(5·2, 6)	253<br>(226, 285)	7·4<br>(6·7, 8·4)	33·2<br>(18·4, 51·3)
**Montenegro**	44<br>(39, 49)	7<br>(6·2, 7·8)	84<br>(76, 94)	8·3<br>(7·5, 9·3)	19·1<br>(2·4, 37·1)
**Poland**	4289<br>(4173, 4408)	9·5<br>(9·3, 9·8)	6178<br>(5765, 6621)	8·8<br>(8·2, 9·4)	-7·4<br>(-13·8, -0·8)
**Romania**	1732<br>(1675, 1792)	6<br>(5·8, 6·2)	3107<br>(2926, 3298)	8·4<br>(7·9, 8·9)	39·2<br>(30·2, 48·3)
**Serbia**	875<br>(760, 982)	7·6<br>(6·6, 8·6)	1559<br>(1438, 1677)	9·7<br>(8·9, 10·4)	26·2<br>(10·1, 48·1)
**Slovakia**	485<br>(462, 509)	7·9<br>(7·6, 8·3)	868<br>(800, 941)	9·5<br>(8·8, 10·3)	19·9<br>(8·6, 31·6)
**Slovenia**	215<br>(206, 224)	8·7<br>(8·3, 9)	406<br>(376, 440)	9·3<br>(8·6, 10)	6·9<br>(-2·2, 16·7)
**Central Asia**	1234<br>(1125, 1324)	2·7<br>(2·4, 2·9)	3463<br>(3315, 3617)	5<br>(4·8, 5·2)	87·9<br>(75·9, 106·3)
**Armenia**	194<br>(177, 211)	7·4<br>(6·7, 8)	388<br>(367, 412)	9·3<br>(8·8, 9·8)	26<br>(13·6, 41·2)
**Azerbaijan**	183<br>(154, 216)	3·7<br>(3, 4·4)	505<br>(450, 563)	5·9<br>(5·2, 6·6)	58·8<br>(33·7, 91·9)
**Georgia**	192<br>(171, 226)	3·1<br>(2·7, 3·6)	329<br>(305, 354)	5·5<br>(5·2, 6)	81<br>(57·6, 103·7)
**Kazakhstan**	174<br>(152, 193)	1·4<br>(1·2, 1·6)	948<br>(878, 1032)	5·7<br>(5·3, 6·2)	302·8<br>(262·4, 355·6)
**Kyrgyzstan**	123<br>(113, 133)	4<br>(3·7, 4·4)	173<br>(159, 191)	4·1<br>(3·8, 4·5)	0·4<br>(-9·9, 11·4)
**Mongolia**	31<br>(28, 34)	3<br>(2·8, 3·3)	99<br>(87, 112)	4·8<br>(4·2, 5·4)	57<br>(35·8, 80)
**Tajikistan**	93<br>(80, 109)	3·4<br>(2·9, 4)	182<br>(164, 201)	3·9<br>(3·5, 4·3)	14·9<br>(-4·9, 36·9)
**Turkmenistan**	27<br>(25, 29)	1·5<br>(1·4, 1·6)	131<br>(120, 142)	3·8<br>(3·5, 4·1)	151<br>(124·1, 180·9)
**Uzbekistan**	218<br>(153, 259)	1·9<br>(1·3, 2·3)	707<br>(634, 790)	3·8<br>(3·4, 4·2)	94·1<br>(57·8, 184·6)
**Central Latin America**	3363<br>(3304, 3416)	4·1<br>(4, 4·2)	10118<br>(9742, 10467)	4·5<br>(4·3, 4·6)	8·2<br>(3·8, 12·8)
**Colombia**	850<br>(821, 880)	5<br>(4·8, 5·2)	2123<br>(1880, 2363)	3·9<br>(3·5, 4·4)	-21·3<br>(-30·3, -11·6)
**Costa Rica**	59<br>(56, 62)	3·4<br>(3·3, 3·6)	308<br>(282, 339)	6·4<br>(5·8, 7)	85·9<br>(67·2, 107)
**El Salvador**	50<br>(47, 53)	1·7<br>(1·6, 1·8)	260<br>(219, 306)	4·6<br>(3·8, 5·4)	166·5<br>(121·5, 218·7)
**Guatemala**	48<br>(46, 51)	1·4<br>(1·3, 1·5)	356<br>(320, 393)	3·4<br>(3·1, 3·8)	143·6<br>(116·2, 172·4)
**Honduras**	46<br>(41, 52)	2·3<br>(2, 2·6)	242<br>(195, 286)	4·2<br>(3·4, 5)	84·2<br>(44·9, 127·2)
**Mexico**	2099<br>(2060, 2138)	5<br>(4·9, 5·1)	5219<br>(5063, 5369)	4·7<br>(4·5, 4·8)	-5·5<br>(-9, -2)
**Nicaragua**	27<br>(24, 29)	1·8<br>(1·6, 1·9)	147<br>(131, 166)	3·4<br>(3, 3·8)	90·6<br>(62·1, 122·7)
**Panama**	40<br>(38, 42)	2·7<br>(2·6, 2·9)	162<br>(150, 175)	4·1<br>(3·8, 4·5)	50·5<br>(36·6, 66·7)
**Venezuela**	144<br>(136, 151)	1·5<br>(1·4, 1·6)	1301<br>(1121, 1498)	4·8<br>(4·1, 5·5)	216·4<br>(171, 269·2)
**Andean Latin America**	445<br>(418, 477)	2·2<br>(2·1, 2·4)	2437<br>(2212, 2655)	4·6<br>(4·2, 5)	107·3<br>(85·3, 132·8)
**Bolivia**	105<br>(83, 127)	3·4<br>(2·7, 4·1)	471<br>(382, 589)	5·7<br>(4·7, 7·1)	67·8<br>(28·5, 116·6)
**Ecuador**	90<br>(86, 95)	1·7<br>(1·6, 1·8)	647<br>(588, 714)	4·5<br>(4·1, 5)	162·3<br>(135·5, 194·1)

**Peru**	249 (220, 274)	2·1 (1·9, 2·3)	1320 (1135, 1511)	4·3 (3·7, 5)	104·5 (72·7, 142·6)
**Caribbean**	447 (429, 469)	1·7 (1·6, 1·8)	2467 (2296, 2654)	4·9 (4·5, 5·2)	184 (164·7, 202·6)
**Antigua and Barbuda**	1 (1, 1)	1·5 (1·4, 1·7)	5 (4, 5)	4·7 (4·3, 5·1)	205·1 (171·3, 242·3)
**The Bahamas**	3 (2, 3)	1·7 (1·6, 1·8)	15 (14, 17)	4·3 (3·9, 4·7)	146 (120·7, 175·9)
**Barbados**	4 (4, 4)	1·4 (1·3, 1·5)	29 (26, 32)	5·9 (5·3, 6·5)	331·6 (284·2, 381·1)
**Belize**	2 (1, 2)	1·7 (1·5, 1·8)	13 (12, 14)	5·4 (5, 5·8)	220·3 (187·7, 260·4)
**Bermuda**	1 (1, 1)	1·9 (1·7, 2)	11 (10, 12)	8·2 (7·5, 9)	336·7 (282·1, 394·7)
**Cuba**	180 (172, 189)	1·7 (1·6, 1·8)	984 (877, 1095)	5·2 (4·6, 5·8)	202·5 (167·2, 239·9)
**Dominica**	1 (1, 1)	1·5 (1·4, 1·6)	6 (5, 6)	6·2 (5·7, 6·8)	304·8 (262·3, 353)
**Dominican Republic**	54 (49, 59)	1·5 (1·3, 1·6)	429 (363, 502)	4·7 (4, 5·5)	222·3 (161·7, 292·6)
**Grenada**	1 (1, 1)	1·5 (1·4, 1·6)	11 (10, 11)	6·6 (6·1, 7·2)	337·5 (290·9, 384·8)
**Guyana**	5 (5, 6)	1·4 (1·3, 1·5)	25 (22, 28)	4·2 (3·8, 4·8)	195·5 (156·3, 237·9)
**Haiti**	56 (45, 71)	1·8 (1·5, 2·3)	201 (153, 253)	3·3 (2·6, 4·1)	79·5 (41·3, 128·3)
**Jamaica**	26 (24, 28)	1·4 (1·3, 1·5)	125 (106, 148)	4·3 (3·6, 5·1)	201·9 (147·9, 269·4)
**Puerto Rico**	76 (72, 80)	2 (1·9, 2·1)	370 (343, 399)	5·1 (4·7, 5·5)	154·6 (131·5, 179·5)
**Saint Lucia**	1 (1, 2)	1·6 (1·5, 1·7)	12 (11, 14)	6 (5·5, 6·6)	277·3 (239, 321·4)
**Saint Vincent and the Grenadines**	1 (1, 1)	1·5 (1·4, 1·7)	7 (6, 7)	5 (4·6, 5·4)	220·6 (187·7, 256·8)
**Suriname**	3 (3, 4)	1·3 (1·2, 1·4)	32 (29, 36)	5·7 (5·1, 6·3)	328·1 (271·3, 390·1)
**Trinidad and Tobago**	12 (11, 12)	1·4 (1·3, 1·5)	89 (74, 107)	5 (4·2, 6)	266 (203·1, 341·5)
**Virgin Islands**	3 (3, 3)	3·6 (3·3, 4)	15 (13, 17)	8·3 (7·1, 9·3)	128·1 (90·2, 164·9)
**Tropical Latin America**	4279 (4162, 4374)	4·9 (4·8, 5)	12830 (12509, 13198)	5·6 (5·5, 5·8)	14·1 (10·6, 19·3)
**Brazil**	4227 (4114, 4319)	5 (4·9, 5·1)	12555 (12233, 12912)	5·6 (5·5, 5·8)	12·7 (9·4, 18)
**Paraguay**	52 (47, 58)	2·5 (2·2, 2·7)	275 (232, 323)	5·4 (4·6, 6·4)	118·5 (78·5, 169·6)
**East Asia**	26786 (25672, 28087)	3 (2·9, 3·2)	89755 (85883, 93552)	4·5 (4·3, 4·7)	47·5 (37, 57·3)
**China**	25322 (24232, 26615)	3 (2·9, 3·2)	85101 (81202, 88782)	4·5 (4·3, 4·7)	47·5 (36·4, 57·8)
**North Korea**	479 (385, 586)	3 (2·4, 3·6)	970 (786, 1147)	3·1 (2·5, 3·7)	4·8 (-16·9, 31·3)
**Taiwan (Province of China)**	539 (522, 554)	3·4 (3·3, 3·5)	2238 (2106, 2385)	5·8 (5·5, 6·2)	69·8 (59·1, 82)
**Southeast Asia**	6432 (6043, 6918)	2·6 (2·5, 2·8)	19909 (18283, 21437)	3·6 (3·3, 3·9)	38·1 (27·1, 48·2)
**Cambodia**	120 (100, 149)	2·8 (2·3, 3·4)	345 (300, 402)	3·3 (2·9, 3·8)	19·2 (-6·7, 46·9)
**Indonesia**	2333 (2016, 2678)	2·5 (2·2, 2·9)	7174 (6046, 8001)	3·7 (3·2, 4·1)	49·3 (33·4, 67·6)
**Laos**	57 (44, 72)	2·8 (2·3, 3·6)	130 (109, 156)	3·3 (2·8, 4)	17·4 (-11·6, 56·4)

**Malaysia**	198<br>(181, 222)	2·4<br>(2·1, 2·7)	832<br>(732, 975)	3·6<br>(3·2, 4·1)	49·8<br>(23·3, 86·3)
**Maldives**	2<br>(1, 3)	2·6<br>(1·9, 3·2)	7<br>(7, 8)	2·7<br>(2·4, 3)	4·1<br>(-19·2, 49·5)
**Mauritius**	29<br>(27, 31)	4·1<br>(3·8, 4·3)	74<br>(68, 82)	4·6<br>(4·2, 5)	12·5<br>(0·6, 26·7)
**Myanmar**	802<br>(645, 987)	3·6<br>(3, 4·4)	1869<br>(1612, 2211)	4·4<br>(3·8, 5·2)	23·1<br>(-4·2, 52·4)
**Philippines**	750<br>(699, 800)	2·6<br>(2·4, 2·8)	2542<br>(2217, 2885)	3·9<br>(3·4, 4·4)	47·9<br>(27·5, 71·6)
**Sri Lanka**	205<br>(190, 220)	2<br>(1·9, 2·1)	658<br>(542, 770)	2·8<br>(2·3, 3·2)	39·3<br>(13, 64·6)
**Seychelles**	3<br>(3, 4)	6<br>(5·4, 6·5)	6<br>(6, 7)	6·3<br>(5·8, 6·8)	5·4<br>(-7·7, 19·1)
**Thailand**	1163<br>(1071, 1255)	3·4<br>(3·1, 3·7)	3411<br>(3051, 3823)	3·5<br>(3·2, 4)	4·2<br>(-8·4, 18·6)
**East Timor**	6<br>(4, 7)	2·1<br>(1·7, 2·6)	24<br>(18, 32)	3·1<br>(2·5, 4·1)	44·7<br>(8·2, 85·9)
**Vietnam**	756<br>(670, 842)	1·9<br>(1·7, 2·1)	2811<br>(2406, 3286)	3·3<br>(2·8, 3·8)	73·3<br>(43, 109·1)
**Oceania**	73<br>(65, 93)	2·7<br>(2·5, 3·4)	206<br>(181, 252)	3·5<br>(3·2, 4·2)	29·4<br>(15·8, 44·7)
**American Samoa**	1<br>(1, 1)	4<br>(3·5, 4·8)	2<br>(2, 2)	5·5<br>(4·8, 6·3)	36·9<br>(8·2, 68·9)
**Federated States of Micronesia**	2<br>(1, 2)	3·6<br>(3·1, 4·1)	3<br>(2, 3)	4·6<br>(4, 5·2)	28·4<br>(3·9, 54·5)
**Fiji**	10<br>(8, 11)	2·9<br>(2·5, 3·4)	25<br>(21, 28)	3·7<br>(3·2, 4·2)	28<br>(1·6, 64·2)
**Guam**	3<br>(3, 3)	4·1<br>(3·6, 4·6)	11<br>(10, 12)	6·2<br>(5·5, 6·9)	53<br>(25·8, 79·5)
**Kiribati**	1<br>(1, 1)	2·3<br>(2, 2·5)	2<br>(1, 2)	2·9<br>(2·4, 3·4)	29·9<br>(4·1, 59·1)
**Marshall Islands**	1<br>(0, 1)	3·7<br>(2·8, 4·6)	2<br>(1, 2)	5·5<br>(4·2, 6·7)	46·9<br>(21·8, 73·3)
**Northern Mariana Islands**	1<br>(1, 1)	4·6<br>(3·9, 5·5)	3<br>(2, 3)	6<br>(5·3, 6·8)	32·4<br>(7·1, 58·2)
**Papua New Guinea**	41<br>(34, 58)	2·4<br>(2, 3·3)	124<br>(100, 166)	3·1<br>(2·6, 4·1)	31·1<br>(10·2, 53·9)
**Samoa**	2<br>(2, 3)	3<br>(2·5, 3·5)	4<br>(4, 5)	3·3<br>(2·9, 3·8)	10·9<br>(-8·1, 34·2)
**Solomon Islands**	3<br>(3, 4)	2·5<br>(2, 3)	9<br>(8, 11)	3·1<br>(2·6, 3·7)	27·6<br>(4·2, 54·6)
**Tonga**	2<br>(2, 2)	4<br>(3·4, 4·5)	4<br>(3, 4)	4·9<br>(4·3, 5·7)	23·2<br>(2·5, 48·9)
**Vanuatu**	2<br>(1, 3)	3·3<br>(2·3, 4·7)	7<br>(5, 11)	4·6<br>(3·2, 6·7)	40·2<br>(12·7, 75·5)
**North Africa and Middle East**	4892<br>(4356, 6020)	3<br>(2·7, 3·6)	15636<br>(14791, 16414)	4<br>(3·7, 4·1)	33·9<br>(4·2, 49·2)
**Afghanistan**	156<br>(80, 293)	2·2<br>(1·2, 4·2)	268<br>(197, 382)	2·7<br>(2·1, 3·9)	22·6<br>(-13·7, 102·3)
**Algeria**	222<br>(197, 253)	1·9<br>(1·7, 2·1)	903<br>(812, 990)	2·9<br>(2·6, 3·2)	55·3<br>(30·4, 79·4)
**Bahrain**	9<br>(8, 10)	5·6<br>(5, 6·2)	32<br>(29, 37)	4<br>(3·6, 4·5)	-27·7<br>(-39·5, -14)
**Egypt**	491<br>(452, 532)	1·8<br>(1·7, 1·9)	1558<br>(1313, 1808)	2·8<br>(2·4, 3·2)	54·2<br>(31·3, 79·3)
**Iran**	545<br>(501, 623)	2·2<br>(2·1, 2·5)	2723<br>(2591, 2843)	4·1<br>(3·9, 4·3)	85·7<br>(57·6, 105·9)
**Iraq**	272<br>(225, 360)	3·6<br>(3, 4·8)	650<br>(599, 705)	2·9<br>(2·7, 3·2)	-19·3<br>(-39·2, 0·5)
**Jordan**	35<br>(29, 42)	2·6<br>(2·1, 3·1)	211<br>(183, 244)	4·1<br>(3·5, 4·6)	56·8<br>(22·8, 103·2)

**Kuwait**	19 (17, 20)	3·2 (2·9, 3·5)	74 (67, 82)	3·2 (2·9, 3·6)	1·5 (-12, 16·5)
**Lebanon**	79 (69, 91)	3·7 (3·3, 4·2)	285 (253, 320)	5 (4·4, 5·5)	34·3 (11·2, 59·7)
**Libya**	90 (70, 113)	4·9 (3·8, 6·1)	293 (243, 359)	6·8 (5·7, 8·3)	39·8 (-2·4, 85·9)
**Morocco**	269 (237, 303)	1·9 (1·7, 2·2)	884 (740, 1051)	2·9 (2·4, 3·5)	51·6 (23·3, 87·9)
**Palestine**	34 (27, 42)	3·9 (3·1, 4·9)	108 (98, 120)	4·8 (4·3, 5·3)	22·3 (-4·1, 55·4)
**Oman**	13 (11, 16)	2·1 (1·7, 2·5)	57 (47, 69)	3·3 (2·7, 3·9)	59·3 (16·9, 103·2)
**Qatar**	6 (5, 7)	6·6 (5·5, 8)	35 (28, 43)	4·8 (3·9, 5·8)	-27 (-46·8, -2·2)
**Saudi Arabia**	117 (95, 147)	2 (1·6, 2·5)	580 (490, 682)	4·2 (3·6, 4·9)	111·1 (50·2, 180·1)
**Sudan**	135 (109, 175)	1·5 (1·2, 1·9)	411 (324, 522)	2·5 (1·9, 3·1)	63·4 (24·6, 107·1)
**Syria**	104 (90, 123)	2 (1·8, 2·4)	399 (328, 469)	3·3 (2·7, 3·8)	59·2 (16·1, 103·5)
**Tunisia**	95 (81, 110)	2 (1·7, 2·3)	338 (270, 412)	2·9 (2·3, 3·5)	43·9 (5·9, 90·5)
**Turkey**	2114 (1705, 2803)	6 (4·9, 7·9)	5309 (4771, 5861)	6·1 (5·5, 6·7)	2·4 (-24·3, 24·3)
**United Arab Emirates**	18 (12, 29)	4·2 (2·6, 6·5)	241 (146, 357)	6·9 (3·9, 10·5)	65·8 (7·9, 125·5)
**Yemen**	65 (43, 100)	1·4 (0·9, 2)	264 (201, 335)	2·2 (1·7, 2·8)	63·8 (7, 152·6)
**South Asia**	8607 (7784, 9888)	1·6 (1·4, 1·8)	35820 (34115, 37929)	2·9 (2·7, 3)	81·5 (56·5, 103)
**Bangladesh**	731 (616, 911)	1·6 (1·4, 2)	2159 (1767, 2631)	1·9 (1·5, 2·3)	15·2 (-4·5, 37·7)
**Bhutan**	4 (4, 6)	1·9 (1·5, 2·7)	17 (14, 22)	3·1 (2·4, 4)	61·2 (22·9, 118·3)
**India**	6887 (6169, 7970)	1·6 (1·4, 1·8)	30426 (28762, 32699)	3 (2·8, 3·2)	89·7 (60·9, 113)
**Nepal**	136 (93, 191)	1·6 (1, 2·2)	563 (392, 785)	2·8 (2, 3·9)	80·4 (44·8, 120·4)
**Pakistan**	848 (706, 967)	1·5 (1·3, 1·8)	2653 (2194, 3159)	2·7 (2·2, 3·1)	73·5 (41·4, 115·6)
**Southern sub-Saharan Africa**	1211 (1056, 1379)	4·6 (3·9, 5·2)	2871 (2704, 3022)	5·5 (5·2, 5·8)	21·2 (8·7, 37·2)
**Botswana**	23 (19, 28)	4·1 (3·5, 5)	67 (54, 80)	5·6 (4·5, 6·6)	35 (4·3, 74·5)
**Lesotho**	33 (28, 41)	3·5 (3, 4·3)	64 (49, 79)	5·8 (4·5, 7·2)	64·5 (25·2, 118·5)
**Namibia**	19 (16, 21)	2·7 (2·4, 3·1)	48 (41, 57)	3·6 (3·1, 4·3)	35·6 (11·3, 65·9)
**South Africa**	954 (812, 1122)	4·7 (4, 5·5)	2265 (2107, 2423)	5·5 (5·1, 5·8)	16·1 (2·5, 32·4)
**Swaziland**	14 (12, 19)	5·2 (4·4, 6·9)	35 (26, 46)	6·9 (5·2, 8·8)	32 (0·4, 76·2)
**Zimbabwe**	169 (149, 190)	4·3 (3·9, 4·9)	392 (330, 456)	6·2 (5·2, 7·2)	42·1 (17·6, 72·9)
**Western sub-Saharan Africa**	2574 (2156, 3128)	3·1 (2·6, 3·8)	7283 (6271, 8582)	4·5 (3·9, 5·3)	46·1 (23·7, 75)
**Benin**	50 (43, 58)	2·6 (2·2, 3)	194 (153, 246)	4·6 (3·6, 5·7)	78·1 (38·8, 125·5)
**Burkina Faso**	95 (76, 114)	2·3 (1·8, 2·7)	302 (252, 370)	3·8 (3·1, 4·6)	66·2 (34·9, 112·6)
**Cameroon**	147 (123, 171)	3·5 (3, 4·1)	611 (466, 784)	5·9 (4·6, 7·5)	68·9 (33·8, 111·3)

**Cape Verde**	9<br>(8, 10)	3·8<br>(3·3, 4·3)	31<br>(28, 35)	7·3<br>(6·5, 8)	91·2<br>(58·6, 133·6)
**Chad**	52<br>(41, 65)	1·9<br>(1·5, 2·3)	167<br>(140, 200)	3·4<br>(2·8, 4)	81·5<br>(46·2, 130·5)
**Côte d'Ivoire**	73<br>(62, 84)	2<br>(1·7, 2·3)	242<br>(196, 293)	2·7<br>(2·2, 3·2)	34·4<br>(2·4, 68·2)
**The Gambia**	7<br>(5, 8)	1·9<br>(1·7, 2·3)	27<br>(22, 33)	3·1<br>(2·5, 3·7)	58·4<br>(23·3, 98·2)
**Ghana**	316<br>(263, 372)	5·3<br>(4·5, 6·2)	1176<br>(994, 1385)	8·3<br>(7·1, 9·7)	55·9<br>(25·5, 94·2)
**Guinea**	68<br>(61, 76)	2·1<br>(1·9, 2·3)	150<br>(118, 195)	3<br>(2·4, 3·9)	43<br>(10·3, 89)
**Guinea-Bissau**	12<br>(8, 15)	3·1<br>(2·2, 3·8)	31<br>(22, 40)	5<br>(3·6, 6·3)	62·9<br>(29·3, 97)
**Liberia**	28<br>(22, 36)	2·5<br>(2, 3·2)	67<br>(50, 86)	3·9<br>(2·9, 5)	55·6<br>(24·8, 92·6)
**Mali**	93<br>(82, 105)	2·4<br>(2·1, 2·7)	235<br>(190, 290)	3·1<br>(2·5, 3·7)	26·9<br>(0·8, 64·3)
**Mauritania**	32<br>(27, 38)	3·2<br>(2·7, 3·7)	98<br>(79, 118)	5·3<br>(4·3, 6·4)	67·1<br>(30·8, 115·1)
**Niger**	51<br>(41, 64)	1·9<br>(1·6, 2·4)	185<br>(145, 236)	2·8<br>(2·2, 3·6)	47·1<br>(19·7, 85·8)
**Nigeria**	1385<br>(995, 1958)	3·3<br>(2·4, 4·6)	3214<br>(2297, 4473)	4·4<br>(3·2, 6)	33·1<br>(-2·2, 88·6)
**São Tomé and Príncipe**	1<br>(1, 1)	1·3<br>(1·1, 1·5)	2<br>(2, 3)	2·4<br>(1·7, 3·3)	79·9<br>(36·5, 137·8)
**Senegal**	85<br>(74, 97)	2·7<br>(2·4, 3·1)	304<br>(259, 352)	4·5<br>(3·9, 5·3)	66·8<br>(36·2, 102·9)
**Sierra Leone**	42<br>(34, 51)	2·2<br>(1·8, 2·6)	121<br>(98, 147)	3·9<br>(3·2, 4·7)	74·8<br>(31·3, 132·3)
**Togo**	28<br>(24, 32)	2·4<br>(2·1, 2·8)	124<br>(98, 155)	4<br>(3·2, 5)	68·3<br>(35·3, 108·5)
**Eastern sub-Saharan Africa**	1928<br>(1656, 2377)	2·7<br>(2·4, 3·3)	4638<br>(4104, 5153)	3·3<br>(2·9, 3·6)	19·6<br>(-1·6, 46·1)
**Burundi**	63<br>(46, 80)	3<br>(2·3, 3·8)	98<br>(80, 117)	2·7<br>(2·2, 3·1)	-12·5<br>(-32·3, 18·1)
**Comoros**	7<br>(6, 8)	3·4<br>(2·9, 4)	16<br>(13, 20)	3·8<br>(3·1, 4·6)	11·5<br>(-15·1, 42·5)
**Djibouti**	5<br>(3, 6)	3·5<br>(2·5, 4·6)	23<br>(16, 32)	4·6<br>(3·3, 6·2)	30·5<br>(-6, 82·7)
**Eritrea**	30<br>(23, 37)	3·3<br>(2·7, 4)	88<br>(63, 112)	4·3<br>(3·2, 5·3)	30·9<br>(-0·7, 78·5)
**Ethiopia**	542<br>(343, 900)	3·1<br>(2·1, 5)	1098<br>(852, 1341)	3·1<br>(2·4, 3·8)	0·7<br>(-30·5, 60·4)
**Kenya**	188<br>(145, 222)	2·3<br>(1·8, 2·7)	667<br>(539, 797)	3·4<br>(2·7, 4)	44·2<br>(26·9, 60·6)
**Madagascar**	122<br>(107, 141)	2·4<br>(2·1, 2·8)	244<br>(197, 298)	2·5<br>(2·1, 3·1)	4·9<br>(-14·7, 31·2)
**Malawi**	88<br>(61, 107)	2·3<br>(1·7, 2·8)	193<br>(165, 225)	2·8<br>(2·4, 3·2)	19·3<br>(-3·9, 69·3)
**Mozambique**	121<br>(105, 138)	2·2<br>(2, 2·5)	289<br>(229, 361)	3·1<br>(2·5, 3·8)	37·6<br>(4·6, 83·6)
**Rwanda**	89<br>(69, 113)	3·2<br>(2·5, 4)	170<br>(141, 212)	3·3<br>(2·7, 4)	3·1<br>(-23·2, 48·3)
**Somalia**	65<br>(40, 95)	2·7<br>(1·9, 3·9)	184<br>(131, 247)	3·2<br>(2·3, 4·2)	15·1<br>(-18·4, 72·7)
**South Sudan**	71<br>(46, 108)	3·1<br>(2·1, 4·6)	116<br>(82, 165)	3·4<br>(2·5, 4·7)	9·3<br>(-21·7, 56·9)
**Tanzania**	299<br>(239, 353)	2·8<br>(2·3, 3·3)	774<br>(640, 969)	3·4<br>(2·9, 4·3)	22·2<br>(-2, 55·7)
**Uganda**	125<br>(107, 147)	2<br>(1·7, 2·3)	419<br>(354, 494)	3·4<br>(2·9, 4)	69·5<br>(37·6, 105·3)

**Zambia**	112<br>(91, 131)	4·1<br>(3·4, 4·7)	256<br>(205, 302)	4·4<br>(3·6, 5·1)	8·1<br>(-10·6, 29·8)
**Central sub-Saharan Africa**	633<br>(543, 743)	3<br>(2·6, 3·6)	1496<br>(1295, 1700)	3·3<br>(2·9, 3·8)	9·1<br>(-9·4, 29·4)
**Angola**	118<br>(88, 161)	3·3<br>(2·5, 4·3)	397<br>(326, 484)	4·2<br>(3·4, 5·1)	29<br>(-0·3, 72)
**Central African Republic**	35<br>(24, 50)	3·2<br>(2·4, 4·5)	63<br>(43, 82)	3·2<br>(2·4, 4·1)	1·2<br>(-20·4, 28·5)
**Congo**	44<br>(34, 54)	4·3<br>(3·5, 5·1)	114<br>(83, 148)	5·2<br>(3·9, 6·4)	20·9<br>(-12·6, 58·1)
**DR Congo**	406<br>(335, 485)	2·8<br>(2·3, 3·4)	841<br>(674, 1033)	2·8<br>(2·2, 3·5)	-1·1<br>(-22·1, 24·6)
**Equatorial Guinea**	6<br>(4, 9)	3·2<br>(2·3, 4·8)	25<br>(17, 35)	6<br>(4·2, 8·4)	84·7<br>(20·6, 178·3)
**Gabon**	25<br>(20, 32)	4·5<br>(3·8, 5·8)	57<br>(46, 67)	5·9<br>(4·8, 6·9)	30·3<br>(-2·2, 64·4)

Appendix Table 3: Incident cases of pancreatic cancer in 1990 and 2017 for both sexes and percentage change of age-standardised rates by location

	1990		2017		Percentage change in age-standardised rates between 1990 and 2017
	Counts (95% UI)	Rate (95% UI)	Counts (95% UI)	Rate (95% UI)	
**Global**	195413 (192388, 199204)	5 (4.9, 5.1)	447665 (438598, 456296)	5.7 (5.6, 5.8)	13.5 (9.9, 16.5)
**High SDI**	107563 (106576, 108561)	8.3 (8.2, 8.4)	210439 (205078, 215723)	9.3 (9.1, 9.5)	11.8 (8.8, 14.6)
**High-middle SDI**	46618 (45139, 48077)	5.0 (4.8, 5.1)	104712 (101608, 107873)	5.9 (5.7, 6.0)	18.6 (13.2, 23.6)
**Middle SDI**	23620 (22956, 24349)	2.5 (2.4, 2.6)	77874 (74175, 80973)	3.6 (3.5, 3.8)	44.0 (35.5, 50.8)
**Low-middle SDI**	11416 (10594, 12328)	2.1 (1.9, 2.3)	36617 (34650, 39375)	3.2 (3.1, 3.5)	54.6 (40.3, 66.7)
**Low SDI**	5673 (4889, 7029)	1.8 (1.6, 2.2)	16379 (15174, 17600)	2.4 (2.3, 2.6)	37.3 (17.4, 56.6)
**High-income North America**	32338 (31862, 32823)	8.9 (8.7, 9)	61111 (59322, 63147)	9.8 (9.6, 10.2)	11 (7.4, 15.4)
**Canada**	2874 (2770, 3055)	8.6 (8.3, 9.2)	5451 (4970, 6033)	7.9 (7.3, 8.7)	-7.9 (-17.4, 1.9)
**Greenland**	6 (5, 7)	18.4 (16.5, 20.2)	11 (10, 12)	16.1 (14.6, 17.5)	-12.5 (-23.3, 0.7)
**USA**	29458 (29011, 29914)	8.9 (8.8, 9)	55649 (54001, 57477)	10.1 (9.8, 10.4)	13.3 (9.5, 17.8)
**Australasia**	1804 (1756, 1854)	7.5 (7.3, 7.7)	4129 (3748, 4521)	8.3 (7.6, 9.1)	11.1 (0.7, 22.2)
**Australia**	1527 (1482, 1568)	7.6 (7.4, 7.8)	3548 (3171, 3943)	8.5 (7.6, 9.4)	11.3 (-0.6, 24.3)
**New Zealand**	277 (259, 298)	6.8 (6.4, 7.4)	581 (526, 637)	7.5 (6.8, 8.2)	9.6 (-2.9, 23.1)
**High-income Asia-Pacific**	17700 (17430, 17968)	8.8 (8.6, 8.9)	46240 (43540, 48931)	9.8 (9.3, 10.3)	12.3 (6.5, 18.1)
**Brunei**	4 (4, 5)	4.5 (3.9, 5.2)	17 (15, 19)	5.8 (5.1, 6.5)	28.4 (6, 53)
**Japan**	15650 (15409, 15896)	9.1 (8.9, 9.2)	39281 (36775, 41775)	10.5 (9.9, 11.1)	15.3 (9.2, 21.4)
**Singapore**	80 (76, 85)	3.6 (3.3, 3.8)	309 (280, 342)	4.6 (4.1, 5.1)	28.9 (13.8, 46.5)
**South Korea**	1966 (1900, 2039)	6.5 (6.3, 6.8)	6633 (5984, 7316)	7.9 (7.1, 8.7)	20.7 (7.9, 34.1)
**Western Europe**	47053 (46405, 47849)	7.9 (7.8, 8)	82839 (79435, 86522)	9 (8.6, 9.4)	13.9 (9.1, 19)
**Andorra**	4 (3, 5)	7.6 (6, 9.1)	11 (8, 13)	7.8 (5.9, 9.5)	1.6 (-18.2, 27.8)
**Austria**	1083 (1036, 1149)	8.9 (8.5, 9.4)	1926 (1755, 2112)	10.7 (9.8, 11.7)	20.6 (8.6, 32.2)
**Belgium**	1263 (1211, 1341)	7.9 (7.6, 8.4)	1855 (1679, 2072)	8 (7.3, 8.7)	0.4 (-9, 10.9)
**Cyprus**	38 (34, 44)	4.4 (4, 5.2)	120 (107, 136)	6.3 (5.6, 7.1)	42.3 (15, 67.8)
**Denmark**	740 (713, 767)	8.9 (8.6, 9.2)	1118 (1032, 1210)	9.8 (9, 10.6)	9.6 (0.7, 19.2)
**Finland**	801 (771, 834)	10.9 (10.5, 11.4)	1331 (1214, 1446)	10.8 (9.9, 11.7)	-1.7 (-10.9, 8)
**France**	5870 (5677, 6141)	6.9 (6.7, 7.2)	11553 (10512, 12941)	8.3 (7.6, 9.2)	20.4 (9.8, 33.6)
**Germany**	11613 (11136, 12178)	8.9 (8.5, 9.3)	20690 (18198, 23238)	10.8 (9.5, 12.1)	21.9 (8.1, 37.7)
**Greece**	990 (949, 1034)	6.3 (6.1, 6.6)	1914 (1755, 2123)	8 (7.4, 8.7)	26.3 (15.7, 38.2)

**Iceland**	25<br>(23, 27)	8.5<br>(7.9, 9.1)	47<br>(42, 51)	8.6<br>(7.9, 9.4)	1.3<br>(-9.2, 12.8)
**Ireland**	368<br>(355, 385)	8.8<br>(8.4, 9.1)	604<br>(544, 676)	8.2<br>(7.4, 9.2)	-6.4<br>(-15.8, 4.5)
**Israel**	395<br>(375, 415)	8<br>(7.6, 8.3)	989<br>(913, 1075)	8.7<br>(8, 9.4)	9.2<br>(0.5, 18.6)
**Italy**	7236<br>(6978, 7657)	7.9<br>(7.6, 8.4)	13475<br>(12189, 14925)	9.1<br>(8.3, 9.9)	14.7<br>(3, 26.9)
**Luxembourg**	47<br>(44, 50)	8.3<br>(7.9, 8.9)	95<br>(81, 110)	9.6<br>(8.2, 11.1)	15.6<br>(-2, 33.7)
**Malta**	33<br>(31, 35)	7.5<br>(7.1, 8)	80<br>(73, 88)	8.9<br>(8.2, 9.7)	18.2<br>(6.7, 31)
**Netherlands**	1675<br>(1614, 1761)	8.2<br>(7.9, 8.6)	3394<br>(3095, 3736)	9.9<br>(9.1, 10.9)	21.6<br>(10.5, 34.5)
**Norway**	639<br>(623, 654)	9.1<br>(8.8, 9.3)	868<br>(819, 919)	9.1<br>(8.6, 9.6)	0.1<br>(-5.6, 7)
**Portugal**	871<br>(841, 901)	6.3<br>(6, 6.5)	1458<br>(1347, 1578)	6.2<br>(5.7, 6.7)	-1.5<br>(-9.7, 7.6)
**Spain**	3626<br>(3501, 3766)	6.4<br>(6.2, 6.7)	7149<br>(6482, 7942)	7.3<br>(6.7, 8)	14.3<br>(4, 25.8)
**Sweden**	1337<br>(1301, 1373)	8.6<br>(8.4, 8.9)	1583<br>(1493, 1675)	7.6<br>(7.2, 8)	-12<br>(-17.6, -6.1)
**Switzerland**	891<br>(845, 947)	8.2<br>(7.8, 8.6)	1377<br>(1236, 1527)	7.9<br>(7.1, 8.7)	-3.9<br>(-14.5, 7.7)
**United Kingdom**	7464<br>(7343, 7598)	7.9<br>(7.8, 8.1)	11116<br>(10754, 11512)	8.6<br>(8.3, 8.9)	8.5<br>(4.8, 12.6)
**Southern Latin America**	3484<br>(3302, 3667)	7.4<br>(7, 7.8)	6613<br>(6140, 7157)	8<br>(7.4, 8.7)	7.8<br>(-1.7, 17.6)
**Argentina**	2545<br>(2369, 2723)	7.7<br>(7.2, 8.3)	4536<br>(4100, 5056)	8.4<br>(7.6, 9.4)	9<br>(-3.8, 22.9)
**Chile**	582<br>(552, 612)	5.8<br>(5.5, 6.1)	1460<br>(1303, 1628)	6.3<br>(5.6, 7)	7.9<br>(-4.6, 21.2)
**Uruguay**	356<br>(337, 379)	8.9<br>(8.4, 9.4)	615<br>(550, 689)	11.3<br>(10.1, 12.6)	26.5<br>(11.5, 43.2)
**Eastern Europe**	18912<br>(17717, 20274)	6.6<br>(6.1, 7)	25865<br>(25301, 26509)	7.6<br>(7.4, 7.8)	15.7<br>(8.8, 23.2)
**Belarus**	709<br>(668, 752)	5.3<br>(5, 5.6)	968<br>(876, 1085)	6.1<br>(5.5, 6.9)	15.3<br>(4, 27.4)
**Estonia**	174<br>(166, 184)	8.3<br>(7.9, 8.7)	246<br>(211, 287)	9.2<br>(7.9, 10.6)	11.1<br>(-4.8, 29.1)
**Latvia**	314<br>(299, 330)	8.5<br>(8, 8.9)	365<br>(322, 408)	9.1<br>(8.1, 10.3)	7.8<br>(-4.4, 21.3)
**Lithuania**	365<br>(348, 384)	7.8<br>(7.5, 8.2)	494<br>(452, 547)	8.6<br>(7.9, 9.4)	9.9<br>(-0.2, 21.4)
**Moldova**	285<br>(266, 307)	6.2<br>(5.8, 6.6)	383<br>(358, 410)	6.8<br>(6.3, 7.2)	9.7<br>(0.8, 20.1)
**Russia**	13484<br>(12774, 14151)	7.2<br>(6.8, 7.6)	17845<br>(17493, 18224)	7.7<br>(7.5, 7.9)	6.4<br>(1.4, 13.4)
**Ukraine**	3580<br>(3092, 4320)	4.9<br>(4.2, 5.9)	5564<br>(5187, 5967)	7.5<br>(7, 8)	53.3<br>(27.4, 76.4)
**Central Europe**	12132<br>(11907, 12327)	8<br>(7.9, 8.1)	18689<br>(18148, 19249)	8.8<br>(8.5, 9)	9.3<br>(5.6, 13.3)
**Albania**	64<br>(59, 70)	2.9<br>(2.7, 3.2)	220<br>(181, 264)	5.2<br>(4.3, 6.2)	79.4<br>(42.9, 123.7)
**Bosnia and Herzegovina**	273<br>(258, 289)	6.6<br>(6.3, 7)	505<br>(461, 552)	8.4<br>(7.7, 9.1)	26.9<br>(14.9, 39.7)
**Bulgaria**	755<br>(727, 784)	5.9<br>(5.7, 6.1)	1211<br>(1122, 1307)	8.4<br>(7.8, 9)	42.6<br>(31.2, 54.3)
**Croatia**	538<br>(508, 568)	8.3<br>(7.8, 8.9)	823<br>(745, 905)	9.1<br>(8.3, 10)	9.8<br>(-1.7, 21.8)
**Czech Republic**	1573<br>(1517, 1636)	11.2<br>(10.8, 11.7)	2339<br>(2136, 2547)	11.1<br>(10.2, 12.1)	-1<br>(-9.8, 9.1)

**Hungary**	1417<br>(1372, 1463)	9.4<br>(9.1, 9.7)	1877<br>(1759, 1995)	9.8<br>(9.2, 10.4)	4.5<br>(-3, 12)
**Macedonia**	102<br>(95, 109)	5.3<br>(5, 5.7)	241<br>(216, 271)	7.1<br>(6.4, 8)	33<br>(18.5, 51)
**Montenegro**	42<br>(37, 47)	6.7<br>(5.9, 7.4)	78<br>(70, 88)	7.8<br>(7, 8.7)	17.1<br>(0.3, 34.2)
**Poland**	4054<br>(3944, 4164)	9<br>(8.7, 9.2)	5682<br>(5303, 6089)	8.2<br>(7.7, 8.8)	-8.2<br>(-14.8, -1.6)
**Romania**	1690<br>(1636, 1748)	5.8<br>(5.7, 6)	2907<br>(2738, 3090)	8<br>(7.6, 8.5)	37.1<br>(28.4, 46.1)
**Serbia**	844<br>(736, 948)	7.3<br>(6.3, 8.1)	1437<br>(1326, 1549)	9<br>(8.3, 9.7)	24.2<br>(8.3, 45.4)
**Slovakia**	577<br>(549, 607)	9.5<br>(9, 9.9)	983<br>(891, 1076)	10.8<br>(9.8, 11.8)	14.4<br>(2.6, 26.7)
**Slovenia**	204<br>(195, 214)	8.2<br>(7.8, 8.6)	386<br>(352, 423)	8.9<br>(8.2, 9.8)	9<br>(-1.3, 20.1)
**Central Asia**	1192<br>(1095, 1274)	2.5<br>(2.3, 2.7)	3342<br>(3200, 3493)	4.7<br>(4.5, 4.9)	85.8<br>(74.6, 102.4)
**Armenia**	187<br>(171, 203)	6.9<br>(6.3, 7.5)	359<br>(339, 381)	8.6<br>(8.1, 9.1)	24.2<br>(12.2, 38.7)
**Azerbaijan**	178<br>(152, 208)	3.5<br>(2.9, 4.2)	493<br>(440, 550)	5.5<br>(4.9, 6.2)	56.8<br>(33.2, 86.9)
**Georgia**	187<br>(167, 221)	3<br>(2.6, 3.5)	309<br>(287, 333)	5.3<br>(4.9, 5.7)	78.5<br>(54.9, 100.9)
**Kazakhstan**	165<br>(144, 184)	1.3<br>(1.1, 1.5)	924<br>(854, 1007)	5.4<br>(5, 5.9)	313.9<br>(273.3, 368.7)
**Kyrgyzstan**	120<br>(111, 130)	3.9<br>(3.6, 4.2)	169<br>(156, 188)	3.9<br>(3.6, 4.3)	-0.9<br>(-11.1, 10.2)
**Mongolia**	30<br>(27, 33)	2.9<br>(2.6, 3.1)	99<br>(87, 112)	4.5<br>(4, 5.1)	57.3<br>(36.1, 80.5)
**Tajikistan**	88<br>(76, 103)	3.2<br>(2.7, 3.7)	177<br>(160, 196)	3.6<br>(3.3, 4)	14.7<br>(-4.8, 35.4)
**Turkmenistan**	26<br>(24, 28)	1.4<br>(1.3, 1.5)	127<br>(117, 138)	3.5<br>(3.2, 3.8)	152<br>(125.3, 181)
**Uzbekistan**	210<br>(150, 248)	1.8<br>(1.3, 2.2)	684<br>(612, 765)	3.5<br>(3.1, 3.8)	88.5<br>(54.6, 171)
**Central Latin America**	3220<br>(3167, 3273)	3.8<br>(3.8, 3.9)	9543<br>(9196, 9873)	4.2<br>(4, 4.3)	8.4<br>(4.1, 12.7)
**Colombia**	818<br>(790, 847)	4.7<br>(4.5, 4.8)	1970<br>(1737, 2202)	3.7<br>(3.2, 4.1)	-21.6<br>(-30.7, -12.1)
**Costa Rica**	55<br>(53, 58)	3.2<br>(3, 3.3)	286<br>(261, 315)	5.9<br>(5.4, 6.5)	85.7<br>(67.1, 106.8)
**El Salvador**	47<br>(44, 51)	1.6<br>(1.5, 1.7)	242<br>(202, 284)	4.3<br>(3.5, 5)	165.9<br>(121.1, 218.1)
**Guatemala**	47<br>(44, 50)	1.3<br>(1.2, 1.4)	336<br>(302, 370)	3.2<br>(2.8, 3.5)	143.7<br>(116.7, 172.3)
**Honduras**	45<br>(40, 50)	2.2<br>(1.9, 2.4)	235<br>(190, 278)	4<br>(3.2, 4.7)	84.9<br>(45.4, 126.5)
**Mexico**	2007<br>(1969, 2043)	4.6<br>(4.5, 4.7)	4943<br>(4795, 5088)	4.4<br>(4.2, 4.5)	-5.4<br>(-8.7, -2)
**Nicaragua**	25<br>(23, 28)	1.6<br>(1.5, 1.8)	138<br>(123, 155)	3.1<br>(2.8, 3.5)	89.5<br>(61.6, 122)
**Panama**	38<br>(36, 40)	2.5<br>(2.4, 2.7)	150<br>(138, 162)	3.8<br>(3.5, 4.1)	49.7<br>(35.7, 65.5)
**Venezuela**	138<br>(131, 145)	1.4<br>(1.3, 1.5)	1242<br>(1068, 1437)	4.5<br>(3.9, 5.2)	216.8<br>(170.2, 269.8)
**Andean Latin America**	426<br>(400, 457)	2.1<br>(1.9, 2.2)	2263<br>(2051, 2466)	4.2<br>(3.8, 4.6)	104.9<br>(82.8, 130.4)
**Bolivia**	101<br>(79, 122)	3.2<br>(2.5, 3.8)	440<br>(354, 547)	5.2<br>(4.3, 6.5)	64.9<br>(25.8, 114.2)
**Ecuador**	86<br>(82, 91)	1.6<br>(1.5, 1.7)	598<br>(543, 660)	4.1<br>(3.7, 4.5)	157.4<br>(130.9, 188.5)

**Peru**	238<br>(211, 262)	2<br>(1.7, 2.2)	1226<br>(1054, 1405)	4<br>(3.5, 4.6)	103.2<br>(71.8, 141.4)
**Caribbean**	422<br>(405, 445)	1.6<br>(1.5, 1.7)	2317<br>(2150, 2501)	4.6<br>(4.2, 4.9)	184.5<br>(165, 203.8)
**Antigua and Barbuda**	1<br>(1, 1)	1.4<br>(1.3, 1.5)	4<br>(4, 5)	4.3<br>(4, 4.7)	201.4<br>(167.9, 237)
**The Bahamas**	3<br>(2, 3)	1.6<br>(1.5, 1.7)	15<br>(14, 16)	4<br>(3.6, 4.4)	146.3<br>(120.2, 175.6)
**Barbados**	4<br>(4, 4)	1.3<br>(1.2, 1.4)	26<br>(24, 29)	5.5<br>(4.9, 6)	326.4<br>(279.4, 376.7)
**Belize**	1<br>(1, 2)	1.6<br>(1.4, 1.7)	13<br>(12, 14)	5.1<br>(4.7, 5.5)	226.1<br>(192.7, 265.3)
**Bermuda**	1<br>(1, 1)	1.8<br>(1.6, 1.9)	10<br>(9, 11)	7.6<br>(6.9, 8.3)	334.8<br>(278.1, 395.7)
**Cuba**	168<br>(160, 177)	1.6<br>(1.5, 1.7)	916<br>(815, 1027)	4.9<br>(4.3, 5.5)	204.4<br>(168.2, 241)
**Dominica**	1<br>(1, 1)	1.4<br>(1.3, 1.5)	5<br>(5, 6)	5.8<br>(5.3, 6.3)	302.8<br>(260.5, 350.7)
**Dominican Republic**	52<br>(47, 57)	1.4<br>(1.3, 1.5)	409<br>(345, 480)	4.5<br>(3.8, 5.2)	223.6<br>(162.2, 293.9)
**Grenada**	1<br>(1, 1)	1.4<br>(1.3, 1.5)	10<br>(9, 10)	6.3<br>(5.8, 6.8)	339.7<br>(293.7, 387.8)
**Guyana**	5<br>(5, 5)	1.3<br>(1.3, 1.4)	24<br>(22, 28)	4<br>(3.6, 4.5)	198.3<br>(157.7, 241.4)
**Haiti**	55<br>(44, 70)	1.7<br>(1.4, 2.1)	195<br>(148, 248)	3.1<br>(2.4, 3.8)	76.7<br>(38.4, 127.4)
**Jamaica**	24<br>(23, 26)	1.3<br>(1.3, 1.4)	119<br>(100, 142)	4.1<br>(3.4, 4.9)	204.7<br>(150, 275.4)
**Puerto Rico**	71<br>(67, 74)	1.9<br>(1.8, 2)	340<br>(314, 369)	4.8<br>(4.4, 5.2)	156<br>(132.8, 181.1)
**Saint Lucia**	1<br>(1, 1)	1.5<br>(1.4, 1.6)	12<br>(11, 13)	5.6<br>(5.1, 6.2)	278.5<br>(240.2, 323.4)
**Saint Vincent and the Grenadines**	1<br>(1, 1)	1.4<br>(1.4, 1.6)	6<br>(6, 7)	4.7<br>(4.3, 5.1)	224.6<br>(190.6, 260.9)
**Suriname**	3<br>(3, 3)	1.3<br>(1.2, 1.3)	31<br>(27, 34)	5.4<br>(4.8, 5.9)	327.8<br>(270.4, 389)
**Trinidad and Tobago**	11<br>(10, 12)	1.3<br>(1.2, 1.4)	83<br>(69, 100)	4.7<br>(3.9, 5.6)	262.1<br>(199.1, 337.4)
**Virgin Islands**	3<br>(3, 3)	3.4<br>(3, 3.7)	14<br>(12, 16)	7.8<br>(6.7, 8.8)	130.3<br>(91.7, 169.1)
**Tropical Latin America**	4114<br>(3992, 4205)	4.6<br>(4.5, 4.7)	12047<br>(11750, 12405)	5.2<br>(5.1, 5.4)	14.3<br>(10.7, 19.8)
**Brazil**	4064<br>(3947, 4154)	4.6<br>(4.5, 4.7)	11790<br>(11488, 12133)	5.2<br>(5.1, 5.4)	12.9<br>(9.5, 18.3)
**Paraguay**	49<br>(44, 55)	2.3<br>(2, 2.5)	257<br>(217, 302)	5<br>(4.2, 5.8)	119.3<br>(78.6, 169.8)
**East Asia**	26712<br>(25576, 28066)	2.9<br>(2.8, 3.1)	88159<br>(84111, 91988)	4.4<br>(4.2, 4.6)	49.9<br>(38.9, 60.3)
**China**	25250<br>(24125, 26606)	2.9<br>(2.8, 3.1)	83562<br>(79583, 87352)	4.4<br>(4.2, 4.6)	49.9<br>(38.5, 60.7)
**North Korea**	478<br>(381, 583)	2.9<br>(2.3, 3.5)	940<br>(760, 1124)	3<br>(2.4, 3.6)	4.8<br>(-18.2, 31.9)
**Taiwan (Province of China)**	540<br>(523, 556)	3.3<br>(3.2, 3.4)	2236<br>(2071, 2424)	5.8<br>(5.4, 6.3)	75.4<br>(62.1, 90.8)
**Southeast Asia**	6357<br>(5958, 6855)	2.5<br>(2.3, 2.7)	19111<br>(17530, 20603)	3.4<br>(3.1, 3.6)	36.1<br>(25.2, 46.3)
**Cambodia**	119<br>(99, 149)	2.6<br>(2.2, 3.2)	335<br>(290, 392)	3.1<br>(2.7, 3.6)	17.2<br>(-9.5, 44.2)
**Indonesia**	2333<br>(2012, 2689)	2.4<br>(2.1, 2.7)	6919<br>(5821, 7735)	3.4<br>(2.9, 3.8)	45.6<br>(29.6, 63.6)
**Laos**	57<br>(43, 72)	2.7<br>(2.1, 3.4)	127<br>(106, 153)	3.1<br>(2.7, 3.7)	15.6<br>(-13.4, 55.1)

**Malaysia**	189<br>(173, 210)	2.2<br>(2, 2.4)	791<br>(693, 927)	3.3<br>(2.9, 3.8)	50.9<br>(24, 86.1)
**Maldives**	2<br>(1, 3)	2.4<br>(1.8, 3)	7<br>(6, 8)	2.5<br>(2.2, 2.8)	3.2<br>(-20.4, 51.3)
**Mauritius**	28<br>(27, 30)	3.8<br>(3.6, 4.1)	72<br>(65, 79)	4.3<br>(3.9, 4.7)	12.8<br>(1.1, 26.9)
**Myanmar**	795<br>(633, 985)	3.4<br>(2.8, 4.2)	1793<br>(1539, 2135)	4.1<br>(3.6, 4.9)	20.7<br>(-6.7, 50.5)
**Philippines**	751<br>(699, 802)	2.5<br>(2.3, 2.6)	2490<br>(2165, 2831)	3.6<br>(3.2, 4.1)	46.1<br>(25.9, 70.4)
**Sri Lanka**	199<br>(184, 213)	1.9<br>(1.7, 2)	623<br>(512, 731)	2.6<br>(2.1, 3)	38.8<br>(11.7, 64.6)
**Seychelles**	3<br>(3, 4)	5.7<br>(5.1, 6.2)	6<br>(6, 7)	5.9<br>(5.5, 6.4)	4.4<br>(-8.7, 18.3)
**Thailand**	1142<br>(1053, 1230)	3.2<br>(2.9, 3.4)	3232<br>(2891, 3625)	3.3<br>(3, 3.7)	4.6<br>(-8.2, 19.4)
**East Timor**	6<br>(5, 7)	2<br>(1.6, 2.4)	22<br>(17, 30)	2.9<br>(2.3, 3.8)	42.7<br>(6.1, 84.3)
**Vietnam**	725<br>(643, 809)	1.8<br>(1.6, 2)	2671<br>(2287, 3112)	3<br>(2.6, 3.5)	71.1<br>(41, 107)
**Oceania**	74<br>(66, 95)	2.6<br>(2.3, 3.2)	209<br>(182, 254)	3.3<br>(3, 4)	28.7<br>(14.5, 44.7)
**American Samoa**	1<br>(1, 1)	3.7<br>(3.2, 4.4)	2<br>(2, 2)	5.1<br>(4.5, 5.8)	37.9<br>(9, 69.9)
**Federated States of Micronesia**	2<br>(1, 2)	3.3<br>(2.8, 3.9)	3<br>(2, 3)	4.2<br>(3.7, 4.9)	27.9<br>(3.2, 54.9)
**Fiji**	10<br>(8, 11)	2.7<br>(2.3, 3.2)	24<br>(21, 28)	3.4<br>(3, 3.9)	27<br>(1.2, 63)
**Guam**	3<br>(3, 3)	3.8<br>(3.4, 4.2)	10<br>(9, 12)	5.9<br>(5.3, 6.5)	55.9<br>(28.3, 82.1)
**Kiribati**	1<br>(1, 1)	2.1<br>(1.9, 2.3)	2<br>(1, 2)	2.7<br>(2.3, 3.2)	28.5<br>(3.3, 56.5)
**Marshall Islands**	1<br>(0, 1)	3.5<br>(2.6, 4.4)	2<br>(1, 2)	5.1<br>(3.9, 6.4)	46.4<br>(19.8, 73.6)
**Northern Mariana Islands**	1<br>(1, 1)	4.2<br>(3.6, 5.1)	3<br>(2, 3)	5.6<br>(5, 6.3)	33.8<br>(7.7, 59.8)
**Papua New Guinea**	43<br>(35, 61)	2.3<br>(1.9, 3.2)	128<br>(103, 172)	3<br>(2.5, 3.9)	29.5<br>(9, 52.9)
**Samoa**	2<br>(2, 3)	2.8<br>(2.4, 3.3)	4<br>(4, 5)	3.1<br>(2.7, 3.5)	9.3<br>(-9.6, 33)
**Solomon Islands**	3<br>(3, 4)	2.3<br>(1.9, 2.8)	9<br>(8, 11)	2.9<br>(2.5, 3.5)	26.2<br>(3.2, 53.4)
**Tonga**	2<br>(2, 2)	3.7<br>(3.1, 4.2)	4<br>(3, 4)	4.5<br>(4, 5.3)	23.8<br>(2.7, 49.9)
**Vanuatu**	2<br>(1, 3)	3.1<br>(2.2, 4.4)	7<br>(5, 10)	4.3<br>(3, 6.3)	38.7<br>(10.8, 75.6)
**North Africa and Middle East**	4834<br>(4271, 5963)	2.8<br>(2.5, 3.5)	15214<br>(14377, 16007)	3.7<br>(3.5, 3.9)	32.9<br>(3.1, 48.5)
**Afghanistan**	154<br>(76, 292)	2.2<br>(1.1, 4)	270<br>(193, 386)	2.6<br>(2, 3.7)	22.3<br>(-14.7, 107.3)
**Algeria**	214<br>(190, 244)	1.7<br>(1.5, 1.9)	861<br>(777, 942)	2.7<br>(2.4, 2.9)	55.6<br>(31.4, 79)
**Bahrain**	9<br>(8, 10)	5.1<br>(4.6, 5.7)	33<br>(29, 37)	3.7<br>(3.3, 4.2)	-27.4<br>(-39.3, -13.2)
**Egypt**	501<br>(460, 542)	1.7<br>(1.6, 1.9)	1584<br>(1334, 1832)	2.7<br>(2.3, 3)	53.4<br>(30.2, 78.4)
**Iran**	540<br>(494, 618)	2.1<br>(1.9, 2.4)	2593<br>(2468, 2712)	3.8<br>(3.7, 4)	84.3<br>(56.2, 104.9)
**Iraq**	264<br>(217, 348)	3.4<br>(2.8, 4.5)	641<br>(589, 698)	2.8<br>(2.6, 3)	-18.7<br>(-38.9, 1.4)
**Jordan**	35<br>(28, 42)	2.5<br>(2, 2.9)	206<br>(177, 237)	3.7<br>(3.2, 4.3)	52.3<br>(18.9, 98)

**Kuwait**	19<br>(17, 20)	3<br>(2.7, 3.2)	73<br>(66, 81)	3<br>(2.8, 3.4)	1.7<br>(-11.7, 17)
**Lebanon**	78<br>(68, 89)	3.5<br>(3.1, 4)	279<br>(247, 313)	4.8<br>(4.2, 5.4)	35.6<br>(11, 62.2)
**Libya**	88<br>(69, 111)	4.6<br>(3.6, 5.8)	289<br>(239, 354)	6.4<br>(5.4, 7.8)	39.6<br>(-2.7, 86.2)
**Morocco**	258<br>(229, 290)	1.8<br>(1.6, 2)	840<br>(706, 996)	2.7<br>(2.3, 3.2)	50.5<br>(22, 86.2)
**Palestine**	32<br>(26, 40)	3.6<br>(2.9, 4.5)	105<br>(95, 116)	4.4<br>(4, 4.9)	21.9<br>(-4.1, 54)
**Oman**	13<br>(11, 16)	2<br>(1.6, 2.4)	57<br>(47, 69)	3.1<br>(2.5, 3.7)	58.3<br>(16, 102.3)
**Qatar**	6<br>(5, 8)	6.1<br>(5.1, 7.4)	36<br>(29, 45)	4.5<br>(3.7, 5.4)	-26.1<br>(-46.1, -0.7)
**Saudi Arabia**	113<br>(93, 142)	1.8<br>(1.5, 2.3)	588<br>(495, 697)	3.9<br>(3.4, 4.5)	109.7<br>(49.1, 178.6)
**Sudan**	131<br>(105, 169)	1.4<br>(1.1, 1.8)	396<br>(311, 503)	2.3<br>(1.8, 2.9)	62.1<br>(23, 107)
**Syria**	102<br>(89, 121)	1.9<br>(1.7, 2.3)	388<br>(319, 459)	3.1<br>(2.5, 3.6)	58.6<br>(15.3, 103.1)
**Tunisia**	95<br>(81, 110)	1.9<br>(1.6, 2.2)	331<br>(262, 402)	2.8<br>(2.2, 3.3)	44.5<br>(7, 90.2)
**Turkey**	2096<br>(1679, 2789)	5.8<br>(4.7, 7.6)	5111<br>(4606, 5634)	5.8<br>(5.3, 6.4)	1.5<br>(-25, 24.8)
**United Arab Emirates**	19<br>(13, 30)	3.9<br>(2.5, 6.2)	261<br>(160, 386)	6.6<br>(3.7, 10)	67.1<br>(8.1, 128.9)
**Yemen**	65<br>(42, 99)	1.3<br>(0.9, 2)	259<br>(195, 329)	2.1<br>(1.6, 2.6)	61.9<br>(5.1, 154.1)
**South Asia**	8488<br>(7680, 9753)	1.5<br>(1.3, 1.7)	34252<br>(32651, 36377)	2.7<br>(2.5, 2.8)	79.1<br>(54.3, 100.5)
**Bangladesh**	709<br>(599, 881)	1.5<br>(1.3, 1.9)	2051<br>(1678, 2488)	1.7<br>(1.4, 2.1)	14.1<br>(-5.2, 36.8)
**Bhutan**	4<br>(3, 6)	1.8<br>(1.4, 2.5)	17<br>(13, 21)	2.9<br>(2.2, 3.7)	58.4<br>(20.1, 116.8)
**India**	6824<br>(6096, 7898)	1.5<br>(1.3, 1.7)	29059<br>(27506, 31343)	2.8<br>(2.6, 3)	87.1<br>(58.3, 110.5)
**Nepal**	134<br>(92, 187)	1.5<br>(1, 2)	531<br>(369, 743)	2.6<br>(1.8, 3.6)	76.8<br>(41.8, 115.8)
**Pakistan**	817<br>(682, 933)	1.4<br>(1.2, 1.6)	2593<br>(2140, 3094)	2.5<br>(2.1, 2.9)	71.1<br>(39, 113.3)
**Southern sub-Saharan Africa**	1168<br>(1025, 1324)	4.3<br>(3.7, 4.9)	2740<br>(2582, 2892)	5.1<br>(4.8, 5.4)	20.2<br>(8.3, 35.6)
**Botswana**	22<br>(18, 27)	3.8<br>(3.2, 4.6)	63<br>(51, 77)	5.1<br>(4.1, 6)	32.4<br>(1.8, 71)
**Lesotho**	32<br>(27, 40)	3.3<br>(2.8, 4.1)	61<br>(47, 76)	5.4<br>(4.1, 6.7)	63.7<br>(24.1, 118.1)
**Namibia**	18<br>(15, 20)	2.5<br>(2.2, 2.8)	45<br>(38, 53)	3.3<br>(2.8, 3.9)	33.6<br>(9.4, 63.9)
**South Africa**	919<br>(789, 1076)	4.4<br>(3.7, 5.2)	2150<br>(2001, 2301)	5<br>(4.7, 5.4)	14.7<br>(1.6, 30)
**Swaziland**	14<br>(11, 18)	4.9<br>(4, 6.4)	34<br>(25, 44)	6.4<br>(4.7, 8.2)	31.1<br>(-0.9, 74.1)
**Zimbabwe**	163<br>(144, 184)	4<br>(3.6, 4.5)	387<br>(325, 450)	5.8<br>(4.9, 6.7)	43.7<br>(19.2, 75.3)
**Western sub-Saharan Africa**	2468<br>(2070, 3011)	2.9<br>(2.4, 3.5)	7021<br>(6035, 8278)	4.2<br>(3.6, 4.9)	45.7<br>(23.2, 75.3)
**Benin**	49<br>(42, 56)	2.4<br>(2.1, 2.8)	188<br>(148, 239)	4.3<br>(3.4, 5.3)	76.7<br>(37.2, 124.2)
**Burkina Faso**	94<br>(75, 112)	2.2<br>(1.7, 2.6)	299<br>(248, 369)	3.6<br>(3, 4.4)	65.5<br>(34.2, 112.3)
**Cameroon**	145<br>(120, 169)	3.3<br>(2.8, 3.9)	594<br>(452, 762)	5.5<br>(4.3, 7)	66.9<br>(31.7, 109.6)

**Cape Verde**	8<br>(7, 9)	3.6<br>(3.1, 4.1)	29<br>(26, 32)	6.7<br>(6, 7.4)	88.4<br>(56.8, 128.8)
**Chad**	50<br>(40, 63)	1.8<br>(1.4, 2.2)	162<br>(136, 195)	3.2<br>(2.7, 3.8)	80.4<br>(45.3, 130.3)
**Côte d'Ivoire**	74<br>(63, 85)	1.9<br>(1.6, 2.2)	241<br>(195, 291)	2.5<br>(2.1, 3)	33.3<br>(0.6, 67.4)
**The Gambia**	6<br>(5, 8)	1.8<br>(1.6, 2.2)	26<br>(21, 31)	2.9<br>(2.3, 3.5)	56.2<br>(21.3, 96.2)
**Ghana**	311<br>(259, 368)	5<br>(4.2, 5.9)	1152<br>(973, 1356)	7.8<br>(6.6, 9.2)	55.8<br>(24.5, 94.5)
**Guinea**	65<br>(58, 73)	1.9<br>(1.8, 2.2)	144<br>(113, 187)	2.8<br>(2.2, 3.6)	43<br>(9.6, 89.9)
**Guinea-Bissau**	12<br>(8, 15)	2.9<br>(2.1, 3.7)	31<br>(22, 40)	4.7<br>(3.4, 6)	61.1<br>(26.9, 95.8)
**Liberia**	27<br>(21, 34)	2.3<br>(1.9, 3)	64<br>(48, 83)	3.6<br>(2.7, 4.6)	52.8<br>(21.8, 90.5)
**Mali**	92<br>(81, 104)	2.3<br>(2, 2.6)	230<br>(185, 283)	2.9<br>(2.3, 3.5)	25.6<br>(-0.8, 63.9)
**Mauritania**	31<br>(27, 37)	3<br>(2.6, 3.6)	95<br>(76, 114)	5<br>(4, 6)	63.5<br>(27.9, 112)
**Niger**	51<br>(41, 64)	1.8<br>(1.5, 2.3)	182<br>(142, 231)	2.6<br>(2.1, 3.3)	45.5<br>(18.1, 84.1)
**Nigeria**	1303<br>(931, 1843)	3<br>(2.2, 4.2)	3052<br>(2173, 4293)	4<br>(2.9, 5.5)	32.7<br>(-2.7, 88.1)
**São Tomé and Príncipe**	1<br>(1, 1)	1.2<br>(1.1, 1.4)	2<br>(2, 3)	2.2<br>(1.6, 3.1)	78.7<br>(35.7, 136.8)
**Senegal**	82<br>(71, 94)	2.6<br>(2.2, 2.9)	291<br>(248, 338)	4.2<br>(3.6, 4.9)	64.9<br>(34.8, 100.8)
**Sierra Leone**	40<br>(32, 48)	2.1<br>(1.7, 2.5)	116<br>(94, 142)	3.6<br>(2.9, 4.3)	73<br>(29.5, 130.2)
**Togo**	27<br>(23, 32)	2.3<br>(1.9, 2.6)	122<br>(96, 153)	3.8<br>(3, 4.7)	66.6<br>(33.9, 107.1)
**Eastern sub-Saharan Africa**	1891<br>(1616, 2346)	2.5<br>(2.2, 3.1)	4495<br>(3976, 4996)	3<br>(2.7, 3.3)	18.3<br>(-3, 45.5)
**Burundi**	62<br>(45, 80)	2.9<br>(2.1, 3.6)	96<br>(78, 115)	2.5<br>(2, 2.9)	-13.9<br>(-33.7, 17.9)
**Comoros**	7<br>(5, 8)	3.2<br>(2.7, 3.7)	15<br>(12, 19)	3.5<br>(2.8, 4.3)	9.9<br>(-16.4, 41.8)
**Djibouti**	5<br>(3, 6)	3.3<br>(2.4, 4.3)	23<br>(16, 32)	4.2<br>(3, 5.8)	29.5<br>(-7.7, 83.2)
**Eritrea**	30<br>(23, 38)	3.1<br>(2.5, 3.8)	88<br>(63, 112)	4<br>(3, 5)	28.9<br>(-3, 78.2)
**Ethiopia**	536<br>(335, 894)	2.9<br>(1.9, 4.7)	1048<br>(811, 1284)	2.8<br>(2.2, 3.5)	-0.9<br>(-31.9, 60.9)
**Kenya**	186<br>(143, 219)	2.2<br>(1.7, 2.6)	659<br>(534, 787)	3.2<br>(2.6, 3.8)	43.4<br>(26.3, 59.4)
**Madagascar**	121<br>(106, 139)	2.3<br>(2, 2.6)	241<br>(195, 295)	2.4<br>(1.9, 2.9)	3.6<br>(-16.2, 30.1)
**Malawi**	83<br>(56, 102)	2.1<br>(1.5, 2.6)	182<br>(155, 212)	2.5<br>(2.2, 3)	19.8<br>(-4.3, 74)
**Mozambique**	116<br>(101, 132)	2<br>(1.8, 2.3)	278<br>(219, 348)	2.8<br>(2.2, 3.5)	37.9<br>(4.4, 84.6)
**Rwanda**	87<br>(67, 110)	3<br>(2.4, 3.7)	164<br>(136, 204)	3<br>(2.5, 3.7)	1.1<br>(-25.3, 47.6)
**Somalia**	65<br>(39, 96)	2.6<br>(1.7, 3.7)	181<br>(129, 246)	2.9<br>(2.1, 4)	14.3<br>(-19.9, 74.6)
**South Sudan**	69<br>(44, 106)	2.9<br>(1.9, 4.4)	115<br>(81, 164)	3.2<br>(2.3, 4.5)	9.4<br>(-22.5, 60.5)
**Tanzania**	292<br>(229, 346)	2.6<br>(2.2, 3.1)	747<br>(615, 939)	3.2<br>(2.7, 4)	21.3<br>(-3, 56.3)
**Uganda**	122<br>(104, 143)	1.9<br>(1.6, 2.2)	405<br>(341, 475)	3.1<br>(2.7, 3.7)	67.6<br>(35.8, 104.9)

**Zambia**	110<br>(89, 129)	3.8<br>(3.1, 4.4)	250<br>(199, 295)	4.1<br>(3.3, 4.8)	7.2<br>(-11.9, 29.6)
**Central sub-Saharan Africa**	625<br>(534, 736)	2.8<br>(2.4, 3.3)	1468<br>(1268, 1662)	3.1<br>(2.7, 3.6)	8.7<br>(-10.5, 29.7)
**Angola**	118<br>(87, 162)	3.1<br>(2.3, 4.1)	393<br>(323, 482)	3.9<br>(3.2, 4.7)	27.7<br>(-1.8, 71.7)
**Central African Republic**	35<br>(24, 50)	3<br>(2.2, 4.3)	62<br>(43, 82)	3<br>(2.3, 3.9)	0.8<br>(-20.8, 27.8)
**Congo**	43<br>(33, 54)	4<br>(3.2, 4.9)	112<br>(80, 147)	4.8<br>(3.6, 6.1)	19.3<br>(-14.7, 58.2)
**DR Congo**	400<br>(331, 479)	2.6<br>(2.1, 3.1)	823<br>(659, 1009)	2.6<br>(2.1, 3.2)	-1.3<br>(-22.9, 25.4)
**Equatorial Guinea**	6<br>(4, 9)	3.1<br>(2.1, 4.6)	24<br>(16, 34)	5.5<br>(3.8, 7.8)	80.9<br>(16.7, 177.8)
**Gabon**	24<br>(19, 31)	4.2<br>(3.5, 5.5)	55<br>(44, 65)	5.5<br>(4.5, 6.4)	29.7<br>(-3.4, 64.4)

Appendix Table 4: DALYs of pancreatic cancer in 1990 and 2017 for both sexes and percentage change of age-standardised rates by location

	1990		2017		Percentage change in age-standardised rates between 1990 and 2017
	Counts (95% UI)	Rate (95% UI)	Counts (95% UI)	Rate (95% UI)	
**Global**	4380941 (4309037, 4475167)	104.9 (103.2, 107)	9080004 (8894128, 9256346)	112.2 (110, 114.4)	7 (3.3, 9.8)
**High SDI**	2122475 (2104398, 2139751	167.4 (166.0, 168.8)	3508115 (3431262, 3588612)	169.6 (165.9, 173.6)	1.3 (-1.0, 3.7)
**High-middle SDI**	1176316 (1138844, 1213206)	118.9 (115.1, 122.6)	2358514 (2285397, 2431056)	128.6 (124.6, 132.5)	8.2 (3.2, 13.0)
**Middle SDI**	620124 (601955, 640019)	59.7 (58.0, 61.5)	1847413 (1756278, 1922329)	81.1 (77.2, 84.3)	36.0 (27.8, 42.3)
**Low-middle SDI**	297782 (274949, 322416)	49.1 (45.5, 53.1)	912709 (862808, 981014)	74.0 (70.1, 79.5)	50.9 (36.6, 63.7)
**Low SDI**	150650 (127390, 187712)	42.2 (36.4, 52.2)	416395 (387858, 446127)	56.5 (52.5, 60.5)	33.9 (13.3, 55.4)
**High-income North America**	608519 (601456, 614961)	174.9 (172.8, 176.7)	1039446 (1012933, 1073143)	175.8 (171.2, 181.6)	0.5 (-2.5, 4.5)
**Canada**	59814 (58426, 61232)	181.5 (177.5, 185.7)	101312 (94227, 108834)	158.3 (147.1, 170)	-12.8 (-19, -5.9)
**Greenland**	156 (141, 170)	418.1 (378.3, 459.4)	255 (231, 278)	353.2 (322.6, 384.8)	-15.5 (-25.5, -2.3)
**USA**	548536 (541980, 554289)	174.2 (172, 176.2)	937862 (912374, 968458)	177.9 (172.9, 183.9)	2.1 (-1.1, 6.3)
**Australasia**	33658 (32909, 34465)	140.5 (137.5, 143.8)	66154 (60448, 72235)	144.8 (132.2, 158.3)	3.1 (-6.2, 13)
**Australia**	28098 (27372, 28805)	140.7 (137.3, 144.3)	56279 (50819, 62396)	146.4 (131.9, 162.6)	4 (-6.6, 15.9)
**New Zealand**	5560 (5319, 5832)	139.2 (133.2, 146)	9875 (9211, 10637)	136.3 (127.1, 146.9)	-2 (-9.4, 6.1)
**High-income Asia-Pacific**	338678 (334193, 343623)	161.1 (159, 163.4)	654958 (632650, 681450)	162.7 (156.8, 169.9)	1 (-2.8, 5.2)
**Brunei**	111 (97, 126)	100 (86.8, 114)	422 (380, 468)	124.2 (111.8, 137.9)	24.2 (3.2, 46.1)
**Japan**	285850 (282017, 289930)	162.4 (160.2, 164.7)	528734 (511265, 549622)	172.2 (166.2, 179.9)	6 (2.4, 10.4)
**Singapore**	1911 (1816, 2010)	77.4 (73.6, 81.4)	5956 (5449, 6523)	84.5 (77.5, 92.6)	9.1 (-1.2, 21.7)
**South Korea**	50806 (49109, 52572)	151.9 (146.8, 157.3)	119846 (110061, 131500)	139.2 (127.9, 152.5)	-8.4 (-16.3, 0.6)
**Western Europe**	936850 (925996, 947001)	164.1 (162.3, 165.8)	1415235 (1364591, 1471686)	172.4 (166.2, 179.5)	5.1 (1.3, 9.5)
**Andorra**	98 (76, 117)	162.4 (125.5, 194.7)	205 (157, 250)	155.9 (119.6, 191)	-4 (-23.5, 19.9)
**Austria**	21816 (21169, 22471)	189.2 (183.5, 195.1)	32338 (30250, 34760)	196.7 (184.4, 211.9)	3.9 (-3.5, 12.5)
**Belgium**	25461 (24602, 26312)	166.6 (161.2, 172.3)	33819 (31359, 36521)	162.5 (150.4, 175.8)	-2.5 (-10.2, 5.7)
**Cyprus**	837 (739, 986)	97.9 (86.5, 115.9)	2509 (2227, 2824)	134.6 (119.6, 151.8)	37.6 (10.1, 65.1)
**Denmark**	14512 (14015, 15023)	186.3 (179.9, 193.1)	20458 (18985, 22018)	189.8 (176.4, 203.9)	1.9 (-6.3, 10.9)
**Finland**	14110 (13688, 14550)	197.5 (191.6, 203.6)	21363 (19768, 23029)	190.4 (176.3, 205.3)	-3.6 (-11.3, 5.1)
**France**	120583 (117574, 124081)	150.6 (146.9, 154.6)	208680 (193467, 225809)	172.9 (160.1, 188.4)	14.8 (5.8, 25.9)
**Germany**	222391 (216078, 228308)	178 (173.2, 182.7)	337219 (301517, 374333)	198.4 (176.6, 220.8)	11.4 (-1, 24.6)

**Greece**	21223<br>(20356, 22030)	138.1<br>(132.6, 143.2)	35468<br>(33151, 37891)	171<br>(159.3, 183.1)	23.8<br>(14.6, 33.6)
**Iceland**	513<br>(483, 544)	181.4<br>(171.1, 192.6)	864<br>(806, 925)	170<br>(158.5, 182)	-6.3<br>(-14.5, 1.8)
**Ireland**	7726<br>(7452, 8020)	186.7<br>(180, 194)	11310<br>(10316, 12403)	159<br>(144.8, 174.6)	-14.9<br>(-22.6, -6.1)
**Israel**	8320<br>(7909, 8747)	168.4<br>(160.2, 177)	19344<br>(17919, 20929)	178.8<br>(165.8, 193.6)	6.2<br>(-2.3, 15.3)
**Italy**	151015<br>(147114, 155088)	170.8<br>(166.5, 175.3)	210986<br>(195925, 229251)	164.5<br>(152.5, 178.9)	-3.7<br>(-11.1, 4.7)
**Luxembourg**	997<br>(941, 1057)	181.1<br>(171.1, 191.8)	1734<br>(1507, 1954)	186.6<br>(162.1, 210.4)	3<br>(-11.3, 17.8)
**Malta**	713<br>(670, 757)	162.1<br>(152.6, 171.8)	1562<br>(1441, 1684)	183.8<br>(170, 197.7)	13.4<br>(3.3, 24.2)
**Netherlands**	34740<br>(33776, 35729)	175<br>(170.1, 179.8)	63054<br>(58973, 67419)	196.8<br>(184.1, 210.7)	12.4<br>(4.1, 21.1)
**Norway**	11027<br>(10830, 11220)	170.5<br>(167.4, 173.6)	14313<br>(13763, 15000)	161.5<br>(155.2, 169.4)	-5.3<br>(-9.4, 0)
**Portugal**	19073<br>(18391, 19750)	136.9<br>(132.2, 141.6)	28872<br>(26614, 31390)	136<br>(125.5, 148.3)	-0.7<br>(-9.3, 9)
**Spain**	71468<br>(69612, 73586)	130.7<br>(127.3, 134.6)	124312<br>(115592, 133269)	146.2<br>(135.7, 156.9)	11.9<br>(3.9, 20.7)
**Sweden**	25353<br>(24734, 26023)	176.2<br>(171.7, 180.8)	31083<br>(29278, 32934)	162.8<br>(153.4, 172.7)	-7.6<br>(-13.4, -1.3)
**Switzerland**	17101<br>(16442, 17746)	168.3<br>(161.8, 174.8)	23940<br>(22085, 25977)	151.6<br>(139.5, 164.9)	-9.9<br>(-18.2, -1.1)
**United Kingdom**	146871<br>(145152, 148660)	165.3<br>(163.4, 167.2)	190334<br>(186409, 194978)	163.3<br>(159.9, 167.2)	-1.2<br>(-3.6, 1.4)
**Southern Latin America**	80271<br>(75978, 84619)	167.7<br>(158.9, 176.7)	140589<br>(130052, 152476)	174<br>(160.9, 188.8)	3.8<br>(-6, 13.8)
**Argentina**	58774<br>(54818, 62835)	175.8<br>(164.2, 187.8)	97125<br>(87369, 108550)	185.5<br>(167.5, 207.1)	5.5<br>(-7.1, 18.8)
**Chile**	13561<br>(12792, 14332)	129.7<br>(122.5, 136.9)	30974<br>(27703, 34779)	133.1<br>(118.9, 149.2)	2.6<br>(-9.9, 16.6)
**Uruguay**	7932<br>(7471, 8440)	202.4<br>(190.7, 215.2)	12483<br>(11165, 13981)	247.4<br>(220.6, 277.4)	22.2<br>(7.3, 38.4)
**Eastern Europe**	471057<br>(440220, 508500)	162.8<br>(152, 176)	574773<br>(562165, 589416)	172.8<br>(168.9, 177.4)	6.2<br>(-0.8, 13.2)
**Belarus**	17151<br>(16103, 18239)	128.8<br>(121, 136.7)	22137<br>(19904, 24950)	143.1<br>(128.8, 162)	11.2<br>(0.2, 24.1)
**Estonia**	4048<br>(3860, 4252)	194.2<br>(185, 203.9)	4647<br>(4061, 5323)	190.1<br>(165.7, 218.8)	-2.1<br>(-15.2, 12.1)
**Latvia**	7348<br>(6945, 7730)	199.8<br>(188.8, 210.1)	7628<br>(6730, 8607)	207.4<br>(182.6, 234.8)	3.8<br>(-8.3, 17.8)
**Lithuania**	8608<br>(8213, 9016)	185.7<br>(177.4, 194.5)	9976<br>(9289, 10730)	190.8<br>(176.9, 205.8)	2.8<br>(-5.7, 11.2)
**Moldova**	7510<br>(6998, 8145)	158.7<br>(147.9, 171.7)	9674<br>(9015, 10391)	172.6<br>(161.2, 185.6)	8.7<br>(-0.2, 18.9)
**Russia**	336108<br>(318196, 354399)	178.7<br>(169, 188.7)	387360<br>(380193, 394135)	169.7<br>(166.6, 172.8)	-5<br>(-9.7, 1.1)
**Ukraine**	90285<br>(77470, 110854)	124.2<br>(107.2, 152.5)	133350<br>(124352, 142789)	186.1<br>(173, 199.9)	49.9<br>(23.4, 73.5)
**Central Europe**	287969<br>(282535, 292904)	187.9<br>(184.5, 191.1)	400461<br>(388559, 412967)	197.2<br>(191.3, 203.5)	4.9<br>(1.3, 8.7)
**Albania**	1642<br>(1511, 1786)	71.1<br>(65.4, 77.5)	5056<br>(4141, 6120)	122.4<br>(100.6, 148.2)	72.1<br>(36.7, 114.9)
**Bosnia and Herzegovina**	6933<br>(6557, 7354)	155.7<br>(147.2, 164.8)	11268<br>(10275, 12326)	189.1<br>(173.1, 206.5)	21.4<br>(10, 33.5)
**Bulgaria**	18511<br>(17836, 19227)	143.5<br>(138.3, 149)	26858<br>(24917, 28723)	200.9<br>(186.3, 216.4)	40<br>(28.7, 51.1)
**Croatia**	11728<br>(11274, 12173)	176.7<br>(170.1, 183)	14425<br>(13468, 15549)	173.6<br>(161.9, 187)	-1.7<br>(-9.2, 6.6)

**Czech Republic**	35502 (34347, 36539)	256.1 (248, 263.6)	44666 (41559, 47747)	224.5 (209.2, 239.7)	-12.3 (-18.9, -4.9)
**Hungary**	32991 (32007, 34015)	221.9 (215.4, 228.7)	40834 (38253, 43458)	226.8 (212.4, 241.5)	2.2 (-5, 9.7)
**Macedonia**	2591 (2423, 2774)	129 (120.7, 137.7)	5759 (5164, 6503)	171.9 (154.6, 193.7)	33.3 (18.4, 51.6)
**Montenegro**	1010 (891, 1124)	154.2 (136, 172)	1751 (1556, 1975)	176.4 (157.3, 199)	14.4 (-2.4, 31.3)
**Poland**	97089 (94340, 99761)	213 (207.4, 218.7)	126150 (117742, 135448)	190.6 (177.9, 204.5)	-10.5 (-17, -3.3)
**Romania**	42983 (41560, 44548)	147.1 (142.3, 152.1)	66491 (62571, 70635)	194.3 (182.5, 206.7)	32.1 (23.4, 41.2)
**Serbia**	21234 (18426, 23951)	175.6 (152.8, 197.9)	31450 (28882, 34038)	204.6 (187.9, 222.2)	16.5 (1.7, 36.2)
**Slovakia**	11180 (10610, 11749)	184 (174.4, 193.3)	18353 (16818, 19937)	204.5 (187.7, 222.2)	11.1 (-0.1, 23.6)
**Slovenia**	4575 (4381, 4774)	181.2 (173.9, 189)	7400 (6819, 8042)	185.1 (170.6, 200.5)	2.1 (-6.8, 11.8)
**Central Asia**	29831 (27781, 31731)	59.8 (55.3, 63.7)	84147 (80529, 88316)	108.2 (103.6, 113.4)	81 (70.6, 94.3)
**Armenia**	4668 (4243, 5078)	160.5 (146.8, 174)	8027 (7588, 8486)	191.5 (180.7, 202.2)	19.3 (8.2, 33.3)
**Azerbaijan**	4585 (4044, 5300)	85.4 (73.9, 99.8)	12801 (11330, 14327)	129.9 (115.4, 144.9)	52.1 (31.3, 76.6)
**Georgia**	4749 (4195, 5651)	73.6 (65.4, 87.2)	7268 (6742, 7811)	129.1 (120.1, 138.9)	75.3 (51.8, 98.7)
**Kazakhstan**	3976 (3457, 4461)	29.7 (25.8, 33.1)	23610 (21737, 25865)	131.3 (121.2, 143.4)	342.3 (299.3, 403.4)
**Kyrgyzstan**	3108 (2870, 3378)	97.8 (90.5, 106.1)	4460 (4071, 4969)	94.4 (86.8, 104.8)	-3.5 (-14.4, 8.7)
**Mongolia**	757 (692, 833)	68 (62.4, 74.6)	2746 (2412, 3142)	108.1 (95, 122.5)	58.9 (37.1, 82.6)
**Tajikistan**	2105 (1850, 2462)	71.9 (63, 83.8)	4608 (4135, 5116)	82.8 (74.6, 91.9)	15.1 (-3.7, 35)
**Turkmenistan**	641 (599, 685)	31.8 (29.7, 34)	3253 (2969, 3544)	80.7 (74, 87.8)	154 (126.7, 183.3)
**Uzbekistan**	5242 (3913, 6090)	43.1 (31.6, 50.5)	17374 (15473, 19491)	75.8 (67.7, 84.7)	75.7 (45.6, 142.4)
**Central Latin America**	79398 (77981, 80676)	87.9 (86.4, 89.3)	223279 (215098, 230887)	94.3 (90.9, 97.5)	7.2 (2.8, 11.5)
**Colombia**	20470 (19765, 21206)	107.4 (103.6, 111.3)	44758 (39437, 50426)	83 (73.1, 93.6)	-22.7 (-31.7, -12.8)
**Costa Rica**	1281 (1221, 1345)	70.2 (67, 73.8)	6301 (5753, 6925)	127.5 (116.3, 140.1)	81.4 (63.1, 102.1)
**El Salvador**	1134 (1057, 1214)	36.5 (34, 39.1)	5410 (4490, 6388)	95.8 (79.4, 113.3)	162.3 (116.1, 216.6)
**Guatemala**	1183 (1116, 1253)	29.8 (28.1, 31.5)	8061 (7239, 8878)	71.8 (64.5, 79.1)	141 (113.8, 169.7)
**Honduras**	1131 (1000, 1267)	49.5 (43.6, 55.3)	5465 (4431, 6607)	88.1 (71.2, 105.1)	77.8 (40.4, 119.7)
**Mexico**	49246 (48286, 50144)	106.2 (104, 108.1)	117011 (113224, 120451)	99.7 (96.5, 102.6)	-6.1 (-9.7, -2.6)
**Nicaragua**	628 (569, 686)	37.6 (34, 41.3)	3196 (2820, 3614)	69.1 (61.2, 78.5)	83.7 (56.4, 116.8)
**Panama**	900 (854, 953)	57.5 (54.5, 60.8)	3323 (3060, 3612)	84 (77.5, 91.4)	46.1 (32.5, 61)
**Venezuela**	3425 (3256, 3608)	33 (31.4, 34.8)	29755 (25486, 34583)	102.8 (88, 119.1)	211.2 (165.1, 263.9)
**Andean Latin America**	10605 (9945, 11390)	48.4 (45.4, 52)	51413 (46385, 56234)	94.6 (85.5, 103.5)	95.5 (73.7, 121.3)
**Bolivia**	2575 (1981, 3135)	74.5 (57.9, 90.3)	10369 (8240, 12914)	117.2 (93.6, 146.1)	57.3 (17.9, 110.7)

**Ecuador**	2116<br>(2013, 2225)	37.2<br>(35.4, 39.1)	13346<br>(12053, 14814)	89.5<br>(80.9, 99.3)	140.8<br>(115.6, 170)
**Peru**	5914<br>(5281, 6498)	46.3<br>(41.3, 50.9)	27698<br>(23600, 31919)	90.7<br>(77.3, 104.4)	95.9<br>(65.3, 132.4)
**Caribbean**	9922<br>(9474, 10523)	36.7<br>(35.1, 38.9)	52432<br>(48549, 56876)	102.9<br>(95.3, 111.6)	180<br>(161.3, 198.9)
**Antigua and Barbuda**	17<br>(16, 18)	33.5<br>(31.1, 36.2)	99<br>(91, 108)	96.3<br>(88.4, 105.4)	187<br>(156.1, 223.1)
**The Bahamas**	63<br>(59, 68)	38.2<br>(35.6, 40.9)	375<br>(339, 413)	94.4<br>(85.6, 103.7)	146.9<br>(119.3, 177.2)
**Barbados**	83<br>(78, 88)	29.8<br>(28.1, 31.8)	572<br>(513, 631)	121.9<br>(109.5, 134.1)	308.5<br>(262.6, 357.5)
**Belize**	34<br>(31, 37)	35.1<br>(32.1, 38.2)	334<br>(309, 360)	119.5<br>(110.6, 129)	241<br>(205.7, 285.1)
**Bermuda**	26<br>(24, 28)	39.4<br>(36.5, 42.5)	189<br>(172, 207)	155.7<br>(140.5, 169.8)	294.7<br>(243.7, 349.2)
**Cuba**	3831<br>(3647, 4031)	36.5<br>(34.7, 38.4)	20125<br>(17811, 22501)	110.4<br>(97.6, 123.5)	202.9<br>(167.2, 241.6)
**Dominica**	23<br>(22, 25)	33.1<br>(31, 35.4)	118<br>(107, 128)	131.7<br>(119.7, 143.8)	297.6<br>(255.3, 344.4)
**Dominican Republic**	1304<br>(1180, 1433)	32.3<br>(29.3, 35.4)	9955<br>(8287, 11732)	105.7<br>(88.1, 124.6)	227.2<br>(165, 298.2)
**Grenada**	23<br>(21, 24)	33.2<br>(31, 35.4)	209<br>(193, 225)	147.1<br>(136.1, 159.5)	343.2<br>(295.9, 393.2)
**Guyana**	130<br>(122, 139)	31.6<br>(29.4, 33.7)	633<br>(559, 713)	96.3<br>(85.4, 108.8)	205<br>(166, 250.6)
**Haiti**	1459<br>(1157, 1875)	41.2<br>(33, 52.3)	5030<br>(3776, 6501)	70.4<br>(53.4, 89.5)	70.8<br>(32.6, 125)
**Jamaica**	539<br>(499, 580)	30.4<br>(28.1, 32.7)	2706<br>(2252, 3238)	94<br>(78.1, 112.4)	209.5<br>(150.5, 281.8)
**Puerto Rico**	1578<br>(1497, 1667)	42.2<br>(40, 44.5)	6777<br>(6286, 7360)	103.7<br>(96.1, 112.5)	145.6<br>(123.2, 171.2)
**Saint Lucia**	31<br>(29, 33)	34.3<br>(32.1, 36.5)	273<br>(250, 298)	129.3<br>(118.3, 141)	276.5<br>(238.4, 321)
**Saint Vincent and the Grenadines**	25<br>(23, 27)	33.6<br>(31.4, 36)	153<br>(140, 166)	112.5<br>(102.9, 122.1)	234.4<br>(199.2, 271.1)
**Suriname**	79<br>(73, 86)	29.3<br>(26.8, 31.5)	755<br>(665, 844)	125.6<br>(111, 140.2)	328.9<br>(271.1, 393.1)
**Trinidad and Tobago**	266<br>(250, 283)	30.1<br>(28.3, 32)	1920<br>(1592, 2308)	105.3<br>(87.1, 126.8)	249.8<br>(187.4, 326.9)
**Virgin Islands**	70<br>(62, 77)	76.1<br>(68.2, 84.6)	323<br>(275, 369)	174.9<br>(150.2, 199.3)	130<br>(89.5, 169.7)
**Tropical Latin America**	102470<br>(99495, 104899)	104.2<br>(101.3, 106.7)	279240<br>(271910, 287254)	117.8<br>(114.8, 121.2)	13<br>(9.3, 18.5)
**Brazil**	101303<br>(98440, 103690)	105.6<br>(102.6, 108)	273285<br>(265783, 281266)	118<br>(114.9, 121.5)	11.8<br>(8.1, 17.2)
**Paraguay**	1167<br>(1027, 1307)	50.5<br>(44.4, 56.5)	5955<br>(4995, 7040)	110.1<br>(92.6, 130.1)	118<br>(76.4, 171.9)
**East Asia**	712220<br>(681192, 748298)	71.1<br>(68.1, 74.7)	1993169<br>(1900840, 2078351)	94.5<br>(90.3, 98.6)	32.9<br>(23.2, 42.4)
**China**	674107<br>(644044, 709655)	71<br>(67.9, 74.7)	1890853<br>(1801159, 1974469)	94.3<br>(89.9, 98.5)	32.8<br>(22.6, 42.7)
**North Korea**	12968<br>(10173, 16032)	71.1<br>(56.4, 86.8)	23860<br>(18882, 29054)	73.7<br>(58.4, 89.2)	3.7<br>(-21.8, 32.8)
**Taiwan (Province of China)**	13279<br>(12869, 13695)	76.5<br>(74.3, 78.8)	46348<br>(43371, 49608)	122.1<br>(114.4, 130.5)	59.5<br>(48.4, 72)
**Southeast Asia**	169086<br>(157634, 183284)	59.5<br>(55.7, 64.1)	471502<br>(430721, 509771)	76.7<br>(70.2, 82.9)	29<br>(18.5, 39.1)
**Cambodia**	3188<br>(2593, 3985)	63.4<br>(52.5, 78.9)	8489<br>(7234, 10084)	71<br>(61.1, 83.6)	11.9<br>(-15.1, 39.4)
**Indonesia**	63463<br>(54226, 73313)	57.1<br>(49.3, 65.7)	174149<br>(145289, 195681)	77.8<br>(65.4, 87.1)	36.3<br>(21.1, 54.2)

**Laos**	1532<br>(1138, 1949)	66.2<br>(50.3, 83.5)	3308<br>(2697, 4001)	73.1<br>(60.7, 88)	10.4<br>(-18.7, 52.1)
**Malaysia**	4733<br>(4320, 5273)	48.6<br>(44.2, 54.2)	18842<br>(16228, 22408)	71.5<br>(62.2, 84.2)	47<br>(20.2, 82)
**Maldives**	60<br>(39, 77)	58.5<br>(39.6, 73.5)	174<br>(156, 195)	56<br>(50.1, 62.5)	-4.3<br>(-28.1, 49.8)
**Mauritius**	739<br>(694, 784)	91.7<br>(86.4, 97.2)	1739<br>(1576, 1926)	101<br>(91.8, 111.5)	10.1<br>(-1.9, 23.8)
**Myanmar**	21244<br>(16511, 26768)	83.4<br>(66, 103.3)	44776<br>(37732, 54328)	95.4<br>(81.1, 114.6)	14.4<br>(-12.3, 46.2)
**Philippines**	20534<br>(19114, 21986)	59.5<br>(55.4, 63.5)	64514<br>(55630, 73518)	83.6<br>(72.7, 95.1)	40.5<br>(20, 64.5)
**Sri Lanka**	5096<br>(4735, 5473)	43.5<br>(40.3, 46.7)	14437<br>(11758, 17173)	56.8<br>(46.6, 67)	30.7<br>(5, 57.2)
**Seychelles**	80<br>(72, 88)	139.1<br>(125, 152.8)	155<br>(142, 169)	138.5<br>(127.7, 150.3)	-0.4<br>(-13.6, 14.8)
**Thailand**	30035<br>(27712, 32494)	75.1<br>(69.3, 81.1)	75517<br>(67304, 85361)	76.4<br>(68.1, 86.1)	1.6<br>(-11.2, 16.2)
**East Timor**	161<br>(129, 191)	48<br>(38.7, 57.6)	548<br>(412, 753)	65.8<br>(50, 89.9)	37.1<br>(-1.2, 81.3)
**Vietnam**	17997<br>(15877, 20157)	42.3<br>(37.5, 47.3)	64234<br>(54507, 74906)	68.6<br>(58.4, 80.2)	62.1<br>(32.3, 97.3)
**Oceania**	2080<br>(1825, 2717)	62.8<br>(56, 80.2)	5819<br>(5009, 7232)	79.5<br>(69.6, 96.8)	26.6<br>(11.6, 43.6)
**American Samoa**	22<br>(19, 25)	86<br>(75.4, 101)	55<br>(47, 63)	119<br>(103, 135.2)	38.3<br>(9.9, 69.3)
**Federated States of Micronesia**	40<br>(34, 47)	78.1<br>(66.2, 91)	74<br>(60, 90)	98.6<br>(82.7, 116.3)	26.3<br>(0.2, 54.3)
**Fiji**	259<br>(223, 298)	64<br>(54.9, 74.2)	616<br>(531, 713)	79.2<br>(68.9, 91.4)	23.9<br>(-1.8, 57.1)
**Guam**	74<br>(66, 84)	87.2<br>(77.6, 98.5)	260<br>(231, 289)	139.5<br>(124.4, 154.6)	60.1<br>(31, 87.7)
**Kiribati**	20<br>(18, 22)	49.1<br>(44.3, 54.6)	43<br>(36, 51)	61.4<br>(51.8, 71.7)	25.1<br>(0.3, 53.4)
**Marshall Islands**	15<br>(11, 19)	83.9<br>(62, 104.9)	44<br>(32, 56)	122.5<br>(90, 153.5)	45.9<br>(17.6, 74.1)
**Northern Mariana Islands**	20<br>(16, 25)	95.1<br>(80.3, 116.6)	70<br>(61, 79)	124.9<br>(110, 139.7)	31.4<br>(5.1, 59.6)
**Papua New Guinea**	1236<br>(1005, 1780)	58.1<br>(48.4, 82.1)	3720<br>(2950, 5076)	73.5<br>(60.1, 98.9)	26.4<br>(4.6, 50.8)
**Samoa**	63<br>(53, 75)	69.7<br>(58.6, 82.4)	103<br>(88, 122)	72.9<br>(62.6, 84.5)	4.6<br>(-14.7, 29.9)
**Solomon Islands**	89<br>(74, 110)	57.1<br>(48.1, 69.6)	244<br>(206, 298)	70.2<br>(59.9, 84.2)	22.9<br>(-0.4, 49.6)
**Tonga**	47<br>(40, 54)	82.2<br>(70, 93.1)	85<br>(74, 100)	104.3<br>(90.6, 121.8)	26.9<br>(4.6, 54.2)
**Vanuatu**	56<br>(38, 82)	75.3<br>(51.6, 111.4)	183<br>(124, 276)	102.8<br>(70.2, 154)	36.5<br>(6.8, 79.2)
**North Africa and Middle East**	125844<br>(109700, 154853)	67<br>(59, 82.4)	380420<br>(358287, 400988)	85.7<br>(80.8, 90)	27.9<br>(-0.8, 44.7)
**Afghanistan**	3925<br>(1846, 7429)	51.8<br>(24.6, 97)	7452<br>(5066, 10729)	63.5<br>(45.7, 90.7)	22.7<br>(-16.1, 130.2)
**Algeria**	5310<br>(4744, 5992)	39.6<br>(35.4, 44.7)	20793<br>(18686, 22721)	61.2<br>(55.1, 67.1)	54.7<br>(30.9, 78)
**Bahrain**	231<br>(209, 255)	113.7<br>(102.4, 126.4)	895<br>(780, 1022)	81.1<br>(71.9, 91.9)	-28.7<br>(-40.2, -14.4)
**Egypt**	14065<br>(12826, 15270)	43.1<br>(39.8, 46.6)	44228<br>(37162, 51333)	65.2<br>(55.2, 75.4)	51.3<br>(26.3, 77.8)
**Iran**	14154<br>(12882, 16201)	48.5<br>(44.5, 55.2)	61847<br>(58750, 64959)	85.3<br>(81.1, 89.5)	76.1<br>(49.2, 96.4)
**Iraq**	6961<br>(5649, 9170)	82.9<br>(67.5, 109.6)	17057<br>(15633, 18718)	66.5<br>(61.2, 72.4)	-19.8<br>(-40.4, 1.8)

**Jordan**	949<br>(764, 1152)	59.6<br>(48.7, 71.4)	5224<br>(4499, 6007)	82.1<br>(70.9, 94.5)	37.7<br>(7.1, 81.5)
**Kuwait**	517<br>(480, 556)	67.4<br>(62.1, 73.2)	1884<br>(1693, 2101)	66.3<br>(59.5, 73.6)	-1.6<br>(-15.7, 13.7)
**Lebanon**	2009<br>(1741, 2300)	83.5<br>(72.7, 95.4)	6602<br>(5792, 7556)	106.9<br>(93.7, 122.1)	28<br>(4.2, 54.7)
**Libya**	2275<br>(1763, 2887)	109.7<br>(85.1, 139)	7637<br>(6208, 9379)	150.6<br>(124.4, 184.3)	37.3<br>(-4.4, 85.9)
**Morocco**	6384<br>(5671, 7153)	42.1<br>(37.3, 47.3)	20328<br>(17005, 24349)	61.9<br>(51.8, 73.9)	47<br>(18.7, 84)
**Palestine**	803<br>(648, 1012)	86<br>(69.2, 108.7)	2706<br>(2462, 3002)	102<br>(92.6, 113)	18.6<br>(-7.4, 51.6)
**Oman**	349<br>(283, 433)	45.5<br>(37.1, 56)	1530<br>(1239, 1872)	67.6<br>(55.1, 82)	48.7<br>(7.7, 92.8)
**Qatar**	175<br>(142, 217)	133.6<br>(111.1, 163.8)	1031<br>(835, 1280)	94.3<br>(76.9, 115)	-29.4<br>(-48.7, -3.2)
**Saudi Arabia**	2890<br>(2347, 3670)	42.5<br>(34.5, 53.8)	16276<br>(13452, 19581)	84.1<br>(72, 98.1)	98.1<br>(39, 167.1)
**Sudan**	3309<br>(2573, 4300)	33<br>(26.2, 42.9)	9944<br>(7662, 12741)	52.3<br>(41, 66.7)	58.3<br>(18.8, 103.8)
**Syria**	2682<br>(2294, 3176)	46.2<br>(40, 54.6)	9880<br>(8037, 11811)	71.4<br>(58.4, 84.8)	54.7<br>(13, 98.6)
**Tunisia**	2167<br>(1843, 2519)	40.4<br>(34.5, 46.7)	7239<br>(5720, 8934)	58.2<br>(46, 71.8)	44<br>(5.5, 90.4)
**Turkey**	54333<br>(42791, 72640)	140.9<br>(111.9, 188.1)	122526<br>(110531, 135189)	137.8<br>(124.5, 152.1)	-2.2<br>(-28.5, 22.9)
**United Arab Emirates**	570<br>(402, 925)	93.5<br>(59.4, 151.1)	8307<br>(5191, 12177)	154.9<br>(86.7, 238.3)	65.7<br>(4.9, 131.6)
**Yemen**	1705<br>(1078, 2657)	30.8<br>(19.9, 47.5)	6676<br>(4995, 8676)	48.6<br>(36.8, 62.1)	57.6<br>(-1, 154.2)
**South Asia**	225283<br>(203304, 259760)	34.9<br>(31.6, 40.1)	847963<br>(809838, 897741)	60.5<br>(57.7, 64)	73.2<br>(48.7, 92.3)
**Bangladesh**	18534<br>(15729, 22955)	36<br>(30.5, 44.9)	50908<br>(41097, 61599)	39.9<br>(32.4, 48.3)	10.8<br>(-9.3, 35.2)
**Bhutan**	117<br>(89, 164)	42<br>(32.7, 58.3)	400<br>(307, 516)	63.2<br>(48.9, 81.5)	50.4<br>(12.5, 111)
**India**	182677<br>(162190, 212227)	35<br>(31.3, 40.6)	717037<br>(679172, 772713)	63.3<br>(59.9, 68.2)	81.1<br>(52.3, 102.7)
**Nepal**	3593<br>(2500, 5052)	34.4<br>(23.5, 48.1)	12828<br>(8888, 18122)	57.5<br>(40, 81)	67.4<br>(32.3, 106.5)
**Pakistan**	20363<br>(17087, 23332)	34<br>(28.5, 38.9)	66790<br>(54460, 80095)	56.2<br>(46.2, 66.8)	65.3<br>(33.5, 106.5)
**Southern sub-Saharan Africa**	29664<br>(26631, 33340)	100.1<br>(88.6, 113.6)	67915<br>(63876, 71947)	117.7<br>(110.6, 124.5)	17.6<br>(6.1, 30.4)
**Botswana**	562<br>(453, 704)	89.7<br>(72.9, 110.7)	1525<br>(1211, 1887)	108.7<br>(87.1, 131.7)	21.2<br>(-7.3, 58.8)
**Lesotho**	824<br>(681, 1033)	78.8<br>(65.6, 98.5)	1577<br>(1177, 1981)	126.4<br>(95.3, 158.1)	60.4<br>(19.5, 118)
**Namibia**	445<br>(386, 513)	58.2<br>(50.7, 66.6)	1066<br>(893, 1270)	74.3<br>(62.8, 88.1)	27.6<br>(3.1, 58.1)
**South Africa**	23268<br>(20511, 26883)	103.7<br>(89.9, 121.2)	52322<br>(48692, 56382)	114.8<br>(106.8, 123.4)	10.7<br>(-1, 22.7)
**Swaziland**	369<br>(303, 494)	115<br>(94.7, 153.1)	915<br>(652, 1199)	151.6<br>(109.5, 196.9)	31.8<br>(-1.2, 76.2)
**Zimbabwe**	4196<br>(3681, 4762)	93.7<br>(82.7, 106)	10509<br>(8819, 12290)	140<br>(117.5, 162.5)	49.4<br>(22, 83.6)
**Western sub-Saharan Africa**	60235<br>(50369, 73860)	64.6<br>(54, 78.8)	173726<br>(148875, 206472)	93<br>(79.8, 109.8)	44<br>(20.8, 73.9)
**Benin**	1186<br>(1018, 1371)	56<br>(48.2, 64.8)	4694<br>(3669, 6059)	96.5<br>(75.8, 123.9)	72.2<br>(33.6, 121.2)
**Burkina Faso**	2320<br>(1868, 2783)	49.4<br>(39.9, 59.2)	7439<br>(6173, 9185)	80.3<br>(66.9, 98.8)	62.6<br>(30.4, 111.1)

**Cameroon**	3744<br>(3096, 4391)	77.3<br>(64.2, 90.1)	15179<br>(11518, 19516)	125.6<br>(95.7, 161.4)	62.5<br>(27.8, 104.9)
**Cape Verde**	191<br>(164, 218)	84.1<br>(72.6, 96)	658<br>(591, 727)	151.5<br>(135.6, 167.1)	80.2<br>(49.7, 115.2)
**Chad**	1214<br>(967, 1523)	40.6<br>(32.3, 50.9)	4059<br>(3390, 4911)	71.7<br>(60.1, 86.5)	76.4<br>(41.1, 128.6)
**Côte d'Ivoire**	1972<br>(1658, 2288)	43.3<br>(36.8, 50)	6333<br>(5053, 7719)	57.5<br>(46.2, 69.5)	32.7<br>(0.7, 67.7)
**The Gambia**	169<br>(139, 204)	43.3<br>(36, 51.9)	635<br>(507, 772)	66<br>(52.5, 80.2)	52.3<br>(16.9, 92.9)
**Ghana**	7911<br>(6546, 9427)	115.4<br>(96, 136.1)	28852<br>(24346, 34101)	178.2<br>(150.3, 210.4)	54.4<br>(22.7, 94.1)
**Guinea**	1558<br>(1394, 1747)	44.4<br>(39.8, 49.7)	3493<br>(2743, 4539)	63.4<br>(49.7, 82.6)	42.9<br>(9, 90.3)
**Guinea-Bissau**	306<br>(207, 393)	69.3<br>(47.9, 88.4)	825<br>(564, 1084)	109.2<br>(76.4, 141.3)	57.6<br>(21.7, 94.9)
**Liberia**	650<br>(520, 836)	54.2<br>(43.4, 69.6)	1642<br>(1225, 2137)	81<br>(60.6, 104.5)	49.5<br>(17.7, 89.7)
**Mali**	2433<br>(2131, 2777)	54.9<br>(48.4, 62.1)	5929<br>(4714, 7400)	67<br>(53.6, 83.1)	22.1<br>(-4.5, 61.7)
**Mauritania**	757<br>(649, 893)	70.2<br>(60.4, 83.3)	2239<br>(1796, 2719)	109.1<br>(87.7, 131.8)	55.3<br>(20.4, 103.3)
**Niger**	1333<br>(1069, 1692)	42.5<br>(34.2, 53.7)	4663<br>(3616, 5951)	59.7<br>(46.6, 75.8)	40.5<br>(12.7, 80.5)
**Nigeria**	30710<br>(21500, 44290)	66.4<br>(47, 95)	73806<br>(51728, 106808)	87.2<br>(61.8, 123.2)	31.3<br>(-6.6, 91.1)
**São Tomé and Príncipe**	21<br>(18, 24)	29.7<br>(25.6, 34.6)	56<br>(41, 79)	52.1<br>(37.7, 72.6)	75.5<br>(31.9, 132)
**Senegal**	2053<br>(1780, 2348)	59.2<br>(51.4, 67.7)	7131<br>(6047, 8330)	95.3<br>(80.9, 110.9)	60.8<br>(29.8, 96.6)
**Sierra Leone**	987<br>(783, 1193)	48.3<br>(38.4, 58.2)	2907<br>(2341, 3578)	81.1<br>(65.5, 99.4)	68.1<br>(24, 126.3)
**Togo**	720<br>(613, 840)	52.6<br>(44.9, 61.1)	3181<br>(2485, 4005)	85.9<br>(67.7, 107.3)	63.3<br>(29.2, 101.6)
**Eastern sub-Saharan Africa**	50450<br>(42593, 63075)	60.9<br>(52.1, 75.4)	117880<br>(104145, 131536)	70.3<br>(62.2, 78.4)	15.5<br>(-6.6, 45.9)
**Burundi**	1734<br>(1224, 2243)	71.1<br>(51.2, 90.8)	2649<br>(2159, 3207)	58.4<br>(47.8, 69.5)	-17.8<br>(-37.8, 14.8)
**Comoros**	179<br>(150, 212)	79<br>(66.6, 93.3)	394<br>(315, 490)	82.7<br>(66.4, 102.4)	4.6<br>(-21.3, 37.9)
**Djibouti**	142<br>(99, 190)	80<br>(57.1, 105.7)	627<br>(423, 895)	100.5<br>(69.9, 140.9)	25.7<br>(-12.5, 82)
**Eritrea**	904<br>(671, 1156)	80.4<br>(63.1, 100.2)	2563<br>(1794, 3390)	98.9<br>(71.6, 125.8)	23<br>(-9.6, 78.8)
**Ethiopia**	14254<br>(8707, 24096)	66.7<br>(42.5, 110.6)	26039<br>(20108, 31972)	63.7<br>(49.3, 78.1)	-4.5<br>(-35.4, 62.4)
**Kenya**	4922<br>(3781, 5831)	53.4<br>(41.1, 63.1)	17853<br>(14439, 21357)	76.3<br>(61.7, 91.3)	42.9<br>(24.8, 60.5)
**Madagascar**	3359<br>(2915, 3882)	57.8<br>(50.6, 66.5)	6826<br>(5502, 8416)	58.1<br>(47.1, 70.9)	0.5<br>(-19.6, 28.7)
**Malawi**	2046<br>(1303, 2538)	47.8<br>(31.4, 58.6)	4436<br>(3768, 5222)	58.2<br>(49.4, 68.5)	21.7<br>(-4.3, 90.1)
**Mozambique**	3003<br>(2611, 3411)	46.3<br>(40.4, 52.2)	7268<br>(5668, 9132)	63.8<br>(50.3, 79.9)	37.9<br>(3.4, 87.8)
**Rwanda**	2357<br>(1800, 3021)	72.8<br>(56.5, 92.5)	4329<br>(3545, 5391)	70.5<br>(58.2, 87.7)	-3.2<br>(-30.9, 47.1)
**Somalia**	1864<br>(1041, 2825)	63.1<br>(38.9, 91.7)	5024<br>(3551, 6852)	71.6<br>(51, 96.5)	13.5<br>(-22.9, 85.5)
**South Sudan**	1843<br>(1100, 2935)	70.3<br>(44.2, 108.9)	3178<br>(2235, 4557)	76.7<br>(54.3, 108.7)	9.1<br>(-25.4, 69.7)
**Tanzania**	7629<br>(5804, 9130)	63.2<br>(49.1, 75.3)	19211<br>(15557, 24494)	75.3<br>(61.5, 95.5)	19.2<br>(-5.8, 59.7)

**Uganda**	3190<br>(2682, 3820)	44.6<br>(37.9, 52.9)	10723<br>(8956, 12764)	73.5<br>(61.7, 86.8)	64.7<br>(30.1, 102.3)
**Zambia**	2999<br>(2355, 3561)	92.7<br>(74.6, 108.5)	6687<br>(5320, 7934)	96.4<br>(76.9, 113.9)	3.9<br>(-14.8, 28.6)
**Central sub-Saharan Africa**	16851<br>(14138, 19979)	67.3<br>(57.9, 79)	39484<br>(34052, 44641)	72.3<br>(62.4, 82.1)	7.5<br>(-12.9, 30.1)
**Angola**	3284<br>(2345, 4559)	74.1<br>(55.5, 101.1)	10736<br>(8785, 13238)	92.2<br>(75.8, 112.3)	24.3<br>(-6.4, 70.1)
**Central African Republic**	974<br>(648, 1432)	74.7<br>(52.2, 108)	1788<br>(1174, 2387)	75.4<br>(52.7, 98.3)	0.9<br>(-21.3, 30.2)
**Congo**	1156<br>(853, 1479)	97.9<br>(74.8, 122.1)	2993<br>(2103, 4091)	112.5<br>(81.2, 146.5)	15<br>(-19.8, 57.2)
**DR Congo**	10676<br>(8791, 12836)	61.5<br>(50.9, 73.6)	21939<br>(17473, 26767)	60.4<br>(48.4, 74.4)	-1.8<br>(-24.7, 26)
**Equatorial Guinea**	161<br>(98, 254)	74.8<br>(48.4, 116.5)	620<br>(410, 910)	126.5<br>(85.5, 180.1)	69.2<br>(5.1, 175.4)
**Gabon**	600<br>(477, 798)	100.5<br>(81.4, 132.8)	1407<br>(1101, 1697)	128.2<br>(101.5, 153.2)	27.6<br>(-6.1, 64.2)
